# Chromatin remodeling coordinates chromosome compaction, synaptonemal complex architecture and meiotic crossing-over

**DOI:** 10.21203/rs.3.rs-10849354/v1

**Published:** 2026-09-10

**Authors:** Nathan Palmer, Uladzislava Khauratovich, Laurine Dal Toe, Felix Hartmann, Anzhela Pavlova, Sushil Khanal, Rajvi Patel, Vasiliki Petroulaki, Viktoria Bláhová, Neil Hunter, Thomas Robert, Anton Goloborodko, Joao Matos

**Affiliations:** 1 Max Perutz Labs, Vienna BioCenter, 1030 Vienna, Austria; 2 University of Vienna, Vienna, Austria; 3 Institute of Molecular Biotechnology, Vienna, Austria; 4 Vienna Biocenter PhD Program, a Doctoral School of the University of Vienna and the Medical University of Vienna, 1030 Vienna, Austria; 5 CNRS, CBS, Montpellier, France; 6 HHMI, University of California, Davis, CA 95616.; 7 Department of Microbiology & Molecular Genetics, University of California, Davis, CA 95616.

**Keywords:** Meiosis, cell cycle, recombination, crossover, genetic diversity, genome stability

## Abstract

Crossing-over between maternal and paternal chromosomes during meiosis relies on the maturation of recombination intermediates within the synaptonemal complex (SC). These events unfold within a hierarchically organized environment, in which linear arrays of chromatin loops are anchored to the axes of meiotic chromosomes. Yet, how chromatin organization is integrated with crossover maturation remains poorly understood. Here, we identify the nucleosome remodeler CHD1 as a previously unrecognized component of the mammalian SC. CHD1 localizes at the interface of synapsed homologs through direct interaction with the SC central-element protein SYCE1 and regulates meiotic chromosome organization. Conditional depletion of CHD1 in meiocytes increases chromatin accessibility at recombination hotspots, disrupts chromosome compaction, and reduces the spatial separation between homolog axes. These meiosis-specific structural defects are accompanied by impaired stabilization of crossover-promoting factors at incipient crossover sites. Together, our findings demonstrate that CHD1-mediated nucleosome remodeling shapes meiotic chromosome organization across scales, creating a chromatin environment that supports crossover maturation and fertility.

## INTRODUCTION

Faithful chromosome segregation during meiosis requires the establishment of physical connections between homologous chromosomes ^1,2^. These connections are generated by homologous recombination, which produces reciprocal exchanges between chromosomes known as crossovers. In addition to promoting genetic diversity, crossovers form the basis of chiasmata, the physical linkages that allow homologs to bi-orient and segregate during the first meiotic division ^3^. Failure to form or correctly position crossovers leads to chromosome mis-segregation and aneuploidy, a major cause of infertility and developmental disorders in humans ^4,5^.

Meiotic recombination is initiated by programmed DNA double-strand breaks (DSBs) catalyzed by the SPO11 transesterase ^6,7^. These breaks are repaired by homologous recombination, during which a subset of strand-invasion intermediates is stabilized and ultimately resolved as crossovers ^8-11^. In most sexually reproducing organisms, these events unfold in close spatial and temporal association with the synaptonemal complex (SC), a conserved supramolecular structure that assembles between pre-aligned homologous chromosomes during meiotic prophase I ^2,12,13^. The SC consists of lateral elements associated with homolog axes and a central region that spans the space between the two axes, creating a specialized scaffold within which recombination intermediates are stabilized and matured ^2,12^.

The SC is built upon a pre-existing loop-axis organization of meiotic chromosomes established in pre-meiotic cells. This is reorganized into arrays of chromatin loops anchored to chromosome axes ^14-16^. Although DSB hotspots are generally located within chromatin loops, they are thought to be transiently recruited to the axis before break formation, where SPO11-associated factors initiate recombination ^17-25^. Subsequent repair of these breaks promotes homolog alignment and synapsis, culminating in assembly of the SC between axial/lateral element structures associated with each homolog. Thus, crossover maturation occurs in a chromosomal environment in which chromatin loops, chromosome axes, and the SC central region are physically and functionally integrated.

At the foundation of this architecture lies chromatin. DNA is packaged into nucleosomes, which regulate DNA accessibility, shape the mechanical properties of chromatin, and provide a substrate for higher-order chromosome organization ^26,27^. Nucleosome positioning and composition are dynamically controlled by ATP-dependent chromatin remodelers and histone-modifying enzymes ^28^. These activities regulate transcription, replication, and DNA repair by modulating nucleosome occupancy, spacing and accessibility ^29-31^. Local chromatin states are also central to meiotic recombination. In mice and humans, DSB hotspots are specified in large part by PRDM9, which binds DNA and promotes an accessible chromatin environment enriched for characteristic histone modifications ^32-37^. These chromatin features help recruit downstream recombination factors, and targeted nucleosome remodeling is required to facilitate efficient DSB formation ^38^.

While the chromatin dynamics governing DSB formation and processing are increasingly well defined, much less is known about how chromatin organization contributes to the later stages of meiotic recombination. Following initial DNA strand invasion, local DNA synthesis is required for recombinational repair. Most events are repaired as non-crossovers by synthesis-dependent annealing of the two DSB ends. At designated crossover sites, both DSB ends engage the homolog template to form double-Holliday junctions, that must be stabilized and ultimately resolved to generate crossovers ^39-41^. These events unfold within the context of the SC. Thus, meiotic recombination is likely shaped not only by local nucleosome organization but also by the larger-scale architecture of meiotic chromosomes. How nucleosome remodeling is integrated with chromosome-axis and SC organization during this process remains a central unresolved question.

Here, we investigate the role of the conserved ATP-dependent nucleosome remodeler CHD1 in the organization of meiotic chromosomes in mice. While CHD1 is known to regulate nucleosome dynamics and maintain chromatin accessibility in transcriptional contexts ^42-44^, its role in mammalian meiosis has remained undefined. We find that CHD1 localizes as discrete puncta at the interface of synapsed homologs through direct interaction with the SC central-element protein SYCE1. Conditional depletion of CHD1 in meiocytes causes a multi-scale disruption of chromosome organization: chromatin accessibility increases at active promoters and recombination hotspots, chromatin loops expand, chromosomes become decompacted, and the separation between homolog axes is narrowed. Notably, this reduction in the width of the SC occurs despite preservation of overall SC length and assembly of the tripartite SC scaffold. CHD1-deficient meiocytes initiate recombination efficiently but fail to stabilize crossover-promoting factors at incipient crossover sites, leading to defective crossover maturation and impaired meiotic progression. Together, our findings identify CHD1 as an SC-associated regulator of meiotic chromosome architecture that connects nucleosome remodeling with higher-order chromosome organization to support crossover maturation, meiotic progression, and fertility.

## RESULTS

### CHD1 expression is temporally regulated during meiotic prophase I, peaking in pachytene

To determine whether CHD1 plays a role in mammalian meiosis, we first examined its expression across mouse tissues by Western blotting. CHD1 was detected in all tissues tested, including adult testis and both adult and embryonic ovary (E16.5), which contain meiotic spermatocytes and oocytes, respectively (Fig. 1a). CHD1 was also broadly expressed in somatic tissues, with particularly high levels observed in thymus (Supplementary Fig. 1a). To assess whether CHD1 is specifically expressed in meiocytes within the adult testis, we enriched spermatogenic cell populations by STA-PUT sedimentation ^45^. CHD1 was readily detected in primary spermatocytes (tetraploid F2 fraction) but was markedly reduced in post-meiotic spermatids (F5 and F7 fractions) (Fig. 1b, Supplementary Fig. 1b). Immunofluorescence analysis of testis sections confirmed this pattern. In meiocytes, CHD1 signal was brightest in mid-late pachytene spermatocytes marked by SYCP3 ^46^ and the histone variant H1t ^47^ (Fig. 1c). In contrast, early prophase-I spermatocytes, identified by H3K9me2 staining, and undifferentiated spermatogonial stem cells, identified by SALL4 staining ^48-50^, showed low CHD1 levels (Supplementary Figs. 1c, and 1d). Together, these results indicate that CHD1 is broadly expressed in somatic tissues but exhibits stage-specific enrichment during spermatogenesis, peaking at mid-late stages of meiotic prophase I.

### Conditional depletion of CHD1 reveals a requirement for meiotic progression at the prophase I-metaphase I transition

As constitutive loss of CHD1 results in embryonic lethality ^44^, we generated an inducible, germline-specific knockout model to examine its function during meiosis. To this end, we combined a floxed *Chd1* allele ^42^ with an Mvh-CreERT2 transgene. As depicted in Supplementary Figs. 1e and 1f, in this system, tamoxifen administration induces Cre recombination specifically in germ cells expressing Mouse Vasa Homolog (Mvh/DDX4). Mvh is expressed in both spermatogonia and meiotic spermatocytes ^51-53^. Efficient depletion of CHD1 in spermatocytes was confirmed 15 days after tamoxifen treatment by immunofluorescence, Western blotting, and mRNA sequencing (Supplementary Figs. 1g and 1h, see also Fig. 3g and associated Supplementary Table 2 for mRNA levels). Throughout this study, these mice are referred to as *Chd1* inducible meiotic-null (*Chd1^iMN^*), with tamoxifen-treated Mvh-CreERT2-only mice used as “controls”.

Histological analysis of testes from adult *Chd1^iMN^* mice revealed largely normal testis architecture 15 days after tamoxifen treatment, although TUNEL staining detected a small but significant increase in apoptotic cells relative to controls. By 30 days post-treatment, apoptosis was markedly increased, accompanied by a reduction in testis weight and an increased number of seminiferous tubules lacking post-meiotic cells such as spermatids (Figs. 1d-g).

To determine which cell populations were affected, we co-stained for TUNEL, SYCP3, and the G2/M marker phospho-H3S10 ^54^. The majority of SYCP3-positive spermatocytes were TUNEL-negative, whereas a large fraction of SYCP3, phospho-H3S10 double-positive cells (corresponding to spermatocytes in either metaphase I or II) were TUNEL-positive, accounting for 48% of apoptotic cells in *Chd1^iMN^* testes. In contrast, phospho-H3S10-positive cells lacking SYCP3 signal, corresponding primarily to mitotically dividing spermatogonia, were largely TUNEL-negative (Figs. 1f-h). These results indicate that CHD1 depletion preferentially affects cells entering or progressing through meiotic divisions, while sparing the mitotic germ cell population.

To assess potential defects occurring in metaphase, we analyzed chromosome spreads from control and *Chd1^iMN^* testes. We observed a marked reduction in the proportion of cells at both metaphase I and metaphase II, with decreases of 75% and 68%, respectively, whereas mitotic metaphase figures were unchanged (Figs. 1i and 1j). Consistent with the strong loss of meiotic metaphase-stage cells, we additionally observed abnormal nuclei in *Chd1^iMN^* testes characterized by hyper-condensed chromatin, multinucleation, and micronucleation (Fig. 1k). These features are characteristic of meiotic catastrophe and suggest that most *Chd1^iMN^* meiocytes fail to successfully execute the prophase I-metaphase I transition ^55^. Among the small proportion of cells that reached metaphase I in *Chd1^iMN^* testes, only a minority displayed abnormal chromosome configurations (Figs. 1l and 1m). As further discussed in the sections below, this small population of *Chd1^iMN^* spermatocytes likely correspond to cells with incomplete CHD1 depletion.

Together, these findings demonstrate that CHD1, which peaks in expression during late stages of prophase I, is required for efficient progression through the prophase I-metaphase I transition during meiosis, with its loss leading to apoptosis at the onset of meiotic division.

### CHD1 is dispensable for meiotic prophase I progression

To determine whether the cell death phenotype observed in *Chd1^iMN^* meiocytes arises from defects during prophase I, we analyzed meiotic chromosome spreads stained for markers of prophase I progression. Co-staining for axial and transverse element components of the SC, SYCP3 and SYCP1, revealed that the majority of *Chd1^iMN^* spermatocytes undergo apparently normal synapsis (Fig. 2a). We note that a subset of pachytene stage cells (~10%) displayed subtle synaptic defects. These included separation of chromosomes within the sex body and ectopic synapsis between sex chromosomes and autosomes (Supplementary Figs. 2a-d). Despite these defects, quantification of prophase I substages using SYCP3 and H1t staining revealed no significant differences between control and *Chd1i^MN^* spermatocytes (Fig. 2b).

Moreover, no significant difference in prophase I substage distribution could be detected in STA-PUT-enriched spermatocyte fractions, indicating that this methodology can be used to directly compare control and *Chd1^iMN^* samples (Supplementary Figs. 2e-g). Together, these results indicate that synaptonemal complex assembly and meiotic progression through prophase I are broadly preserved upon CHD1 depletion, with only a minor fraction of pachytene cells exhibiting subtle synaptic defects, primarily involving the sex chromosomes.

### CHD1 supports chromosome compaction during meiotic prophase I

Careful examination of Hoechst-stained prophase-I chromosome spreads revealed that the area occupied by chromatin was increased in *Chd1^iMN^* spermatocytes compared to substage-matched controls (Fig. 2a). Quantification across biological replicates showed a 13-22% increase in chromatin area across all prophase-I substages, indicating abnormal chromosome compaction (Fig. 2c and Supplementary Fig. 2h). To determine whether this phenotype was also evident at the level of individual chromosomes, we performed whole-chromosome FISH using probes against chromosomes 5, 8, X, and Y. In each case, chromosome territories appeared expanded in *Chd1^iMN^* pachytene spermatocytes, with average increases of approximately 20-60% depending on the chromosome analyzed (Fig. 2d’f). The X chromosome showed the most pronounced expansion, consistent with its distinct chromatin environment ^56^. To exclude the possibility that these observations arose from differences in chromosome spreading, we measured nuclear area in live, spermatocyte-enriched populations obtained by STA-PUT fractionation (as in Supplementary Fig. 1b for wild type, validated for control and *Chd1^iMN^* genotypes in Supplementary Figs 2e-g). In these samples, we again observed a reproducible increase in DNA-stained area in *Chd1^iMN^* cells relative to controls, indicating that chromatin expansion is not an artifact of fixation or spreading (Figs. 2g-h).

We next considered the possibility that changes to chromatin compaction could be occurring at the level of nucleosomes. In order to test this, we performed super-resolution imaging of chromatin using an antibody recognizing a broad spectrum of Histone H3 variants (see Methods). In control spermatocytes, H3 signal appeared as densely distributed foci throughout the nucleus. In *Chd1^iMN^* nuclei, a similar pattern was observed, but with visibly reduced density of H3-positive foci (Fig. 2i). Quantification revealed an approximately 32% decrease in H3 signal density, closely matching the increase in nuclear area (Fig. 2j). Importantly, however, the total number of H3-positive foci per nucleus was not significantly changed (Fig. 2k).

To investigate whether chromatin compaction could be altered due to changes in the balance of any core, or linker histone variants, we acid-extracted and purified histones from control and *Chd1^iMN^* spermatocytes (F2 from STA-PUT sedimentation, as in Supplementary Figs. 2e-g) and performed semi-quantitative mass spectrometry analysis. Notably, no significant differences were detected in the levels of core histones or testis-specific histone variants between control and *Chd1^iMN^* samples (Figs. 2l and 2m and Supplementary Table 1).

Together, these observations indicate that CHD1 depletion causes global chromatin decompaction without evidence for reduced nucleosome abundance or altered histone composition, consistent with chromatin occupying a larger nuclear area while maintaining overall nucleosome content.

### CHD1 restrains chromatin accessibility at active gene promoters and sex chromosomes

To investigate whether changes in chromatin compaction triggered by CHD1 loss are associated with altered nucleosome positioning and chromatin accessibility, we performed ATAC-seq on spermatocyte-enriched populations. Analysis of DNA fragment size distributions revealed a reproducible shift in *Chd1^iMN^* samples, with a reduction in the proportion of sub-nucleosomal fragments and a corresponding increase in fragments associated with mono- and di-nucleosomes (Fig. 3a). This change was observed across biological replicates and was noticeably more pronounced when considering reads derived from the sex chromosomes (Supplementary Figs. 3a and 3b). To assess accessibility changes genome-wide, we compared ATAC-seq signal across 2 kb genomic bins between control and *Chd1^iMN^* samples. This analysis revealed a clear skew toward increased accessibility in *Chd1^iMN^*, with many bins deviating from the diagonal corresponding to equal signal between conditions (Fig. 3b). Notably, the bins showing increased accessibility segregated into two broad populations: one comprising regions with high baseline accessibility (in control samples), which increased further upon CHD1 depletion, and a second comprising regions with low baseline signal that showed pronounced gains specifically in *Chd1^iMN^* samples (Fig. 3b).

To define the genomic features underlying these two populations, we performed differential peak analysis and annotated significantly changed peaks (Supplementary Table 3). On autosomes, accessibility gains were dominated by promoter-proximal regions (Supplementary Fig. 3c, purple). In contrast, the sex chromosomes displayed a distinct pattern, with a larger fraction of significantly changed sites mapping to intergenic regions (Supplementary Fig. 3c, grey). Mapping these annotations back onto the genome-wide comparison showed that promoter-associated bins largely accounted for the high-baseline population, whereas bins derived from the sex chromosomes formed the second, distinct population (Fig. 3b, purple and green). Consistently, accessibility changes at transcription start sites were bimodal: a subset of promoters (~30%) increased by 2.2-fold, while the majority remained unchanged (Figs. 3c and 3e). By contrast, accessibility across the X and Y chromosomes increased more uniformly, with average fold changes of 2.2 and 1.8, respectively (Figs. 3d and 3f).

Given the established role of CHD1 in modulating transcription in embryonic stem cells ^42,44,57^, we asked whether the accessibility changes observed were reflected on broad transcriptional reprogramming. mRNA-seq analysis of STA-PUT-enriched spermatocytes revealed only modest expression changes upon CHD1 depletion, with no enrichment for meiosis-related pathways, and none of the modestly affected genes corresponded to factors with established functions in meiotic recombination, synapsis, or cell cycle progression (Figs. 3g-h; Supplementary Table 2). Although promoter accessibility was generally positively associated with transcriptional activity (Supplementary Fig. 3d, R=0.4) and accessibility change was restricted to the promoters of transcriptionally active genes (Supplementary Fig. 3e), promoter accessibility changes did not correlate with changes in steady-state mRNA abundance (R=−0.04; Supplementary Fig. 3f), indicating that increased promoter accessibility observed in *Chd1i^MN^* mutants is generally not accompanied by transcriptional upregulation or downregulation.

Finally, to assess whether the accessibility phenotypes observed in spermatocytes reflect a general consequence of CHD1 loss or instead arise within the meiotic chromatin context, we generated inducible knock-out (*Chd1^iKO^*) mouse embryonic fibroblasts (MEFs) where Chd1 deletion could be induced via addition of 4-OHT to culture media (Supplementary Fig. 3g) and performed ATAC-seq following CHD1 depletion. In contrast to spermatocytes, CHD1 loss in MEFs did not produce a global skew toward increased accessibility across 2 kb genomic bins, nor did it generate the distinct promoter versus sex chromosome-associated populations observed in meiotic cells (compare Supplementary Fig. 3h with Fig. 3b). Fragment size distributions and overall accessibility profiles were broadly similar between control and CHD1-depleted MEFs (Supplementary Fig. 3i), indicating that the pronounced accessibility gains seen in spermatocytes are not an inevitable consequence of CHD1 removal from somatic chromatin.

Together, these data reveal that CHD1 shapes chromatin accessibility in a region-selective manner: its depletion preferentially increases accessibility at active promoters and across broad intergenic domains on the sex chromosomes, while leaving the global transcript levels largely intact. Notably, these accessibility changes are not observed upon CHD1 loss in MEFs, consistent with a chromatin-regulatory function that is specific to, or particularly relevant in, meiotic cells.

### Increased chromatin accessibility is coupled to local DNA loop expansion

In meiotic prophase chromosomes, chromatin loops are anchored to the synaptonemal complex axis. Therefore, the distance of a genomic locus from the axis is a proxy for the extension of the loop it sits on. To understand whether local changes in chromatin accessibility translate to changes in loop expansion, we measured this distance by DNA FISH using sets of sequential fosmid probes spanning approximately 120kb (illustrated in Fig. 3i, adapted from ^58^), specifically targeting regions based on defined ATAC-seq profiles. We analyzed loci on chromosomes 2 and 17 that gained accessibility upon CHD1 depletion (Figs. 3j-l; Supplementary Figs. 3j-l), alongside control loci that showed no detectable accessibility change (Figs. 3m-o; Supplementary Figs. 3m-o). In control spermatocytes, both classes of loci were, on average, positioned at comparable distances from the SC axis. In *Chd1^iMN^* spermatocytes, however, loci that gained accessibility exhibited a reproducible 10-20% increase in fosmid-axis distance, consistent with local chromatin loop expansion. By contrast, loci with unchanged accessibility showed no detectable difference in axis distance between control and mutant cells (Figs. 3j-o; Supplementary Figs. 3j-o). We extended this analysis to the X chromosome, where ATAC-seq indicated a broad increase in accessibility not only at promoter-proximal regions but also at intergenic regions (Supplementary Fig. 3c, compare purple and grey bars). Consistent with this chromosome-wide signature, two X-linked regions - one promoter-proximal and one intergenic - displayed increased distance from the SC axis in *Chd1^iMN^* spermatocytes (Supplementary Figs. 3r and 3u, respectively). Notably, the changes here were more pronounced, with a 20-30% increase in both regions, suggesting more pervasive loop expansion on the X chromosome.

Together, these data link the ATAC-seq phenotype to the compaction defect: chromatin regions that gain accessibility upon CHD1 depletion also become positioned farther from the chromosome axis, whereas regions with stable accessibility maintain their original positioning. This coupling supports a model in which CHD1 restrains chromatin opening at specific genomic regions and, in doing so, modulates local loop extension and higher-order chromatin compaction during meiosis.

### Chromosome axis length is maintained upon CHD1 depletion

Because meiotic loop expansion can be accompanied by chromosome-axis shortening ^59,60^, we asked whether the increased loop extension observed in *Chd1^iMN^* spermatocytes reflected a broader change in axis organization. Measurements of SC length on FISH-identified chromosomes, using SYCP3-stained chromosome spreads, revealed no detectable difference between control and *Chd1^iMN^* spermatocytes (Fig. 2f). This was consistent across multiple chromosomes and nuclei, indicating that chromatin expansion and local loop extension occur without measurable shortening of the chromosome axis.

We next asked whether this conclusion held at higher spatial resolution and whether major axis-associated loop regulators remained properly recruited. STED imaging of SYCP3-labelled axes (green) revealed no systematic changes in axis continuity in *Chd1^iMN^* spermatocytes (Figs. 4a-f,j,l), supporting the diffraction-limited chromosome-spread measurements. Moreover, the meiotic cohesin subunits REC8 and RAD21L (magenta), key determinants of loop-axis organization, formed axis-aligned tracks that partly overlapped with SYCP3 in both control and mutant cells, with no overt differences in their signal density (Figs. 4a-b, d-e).

Together, these observations indicate that CHD1-dependent chromatin expansion and local loop extension occur without detectable axis shortening, disrupted SYCP3 continuity, or overt redistribution of REC8 and RAD21L. Thus, the loop-expansion phenotype is unlikely to reflect a global reorganization of the chromosome axis and instead points to altered packaging or extension of chromatin loops around an apparently preserved axial framework.

### CHD1 modulates the central region of the SC

While assessing SC architecture at super-resolution, we uncovered an unexpected reduction in the distance between SYCP3-marked chromosome axes in *Chd1^iMN^* spermatocytes. In control spermatocytes, SYCP3-labelled axes resolved as two parallel tracks corresponding to the lateral elements of the assembled SC, separated by approximately 193 nm (Figs. 4g-i, Supplementary Figs. 4a-e) as previously reported ^61-63^. In *Chd1^iMN^* spermatocytes, paired SYCP3 tracks retained their overall organization, but their spacing was markedly reduced to approximately 155 nm (Supplementary Fig. 4e). This narrowing was reproducible across biological replicates and remained relatively uniform along chromosome axes within individual nuclei. Consistent with this, the distance between paired SYCP1 C-terminal signals (SYCP1-C), which mark the inner edge of each lateral element ^63^, was similarly reduced, while their relative position with respect to SYCP3 was preserved (Figs. 4c,i, Supplementary Fig. 4c). Together, these findings indicate a global narrowing of the SYCP3/SYCP1-defined SC compartment rather than a locus-restricted effect or a disruption of lateral-element organization.

We next asked whether this narrowing extended to cohesin-associated components of the chromosome axis. As for SYCP3 and SYCP1-C, the distance between paired RAD21L signals was significantly reduced. For REC8, however, no statistically significant change in spacing was detected (Figs. 4a-b, g-h, Supplementary Figs. 4a and 4b). Thus, CHD1 depletion appears to affect available pools of cohesin complexes differently depending on their kleisin subunit. One possible interpretation of this observation is that the connection of RAD21L-cohesin to the axial elements is more spatially restricted than that of REC8-cohesin.

Finally, we asked whether narrowing of the SYCP3/SYCP1-defined SC compartment was accompanied by disruption or compression of the central region. To this end, we co-stained SYCP3 with the central element protein SYCE1 ^64^. In control nuclei, SYCE1 formed a linear track of approximately 60 nm in width, the peak of which is distanced from each SYCP3 axis by a gap of roughly 95 nm (Figs. 4j, 4k and Supplementary Fig. 4f). In *Chd1^iMN^* nuclei, SYCE1-track width was largely maintained, whereas the gap between SYCE1 and the flanking SYCP3 axes was reduced to approximately 71 nm (Supplementary Fig. 4f). Thus, SC narrowing does not appear to reflect compression/disruption of the SYCE1-marked central element itself, but rather reduced spacing between this central region and the SYCP3-defined lateral elements. Unexpectedly, however, CHD1 depletion made the SYCE1-marked central region appear more continuous. Control nuclei often showed heterogeneous SYCE1 tracks with small discontinuities, whereas such interruptions were less frequent in *Chd1^iMN^* nuclei (Fig. 4l). This suggests that, in addition to controlling the spatial relationship between the central region and lateral elements, CHD1 modulates the organization or continuity of central-region components.

### CHD1 depletion increases chromatin accessibility at meiotic recombination hotspots

Because meiotic DSB hotspots are characterized by an intrinsically accessible chromatin state ^37,65^, and yeast Chd1 is required for efficient crossing-over ^66^, we asked whether CHD1 loss alters accessibility at these sites after the main window of DSB formation. To this end, we queried our ATAC-seq dataset, generated from STA-PUT F2 fractions enriched primarily for pachytene spermatocytes, using established SPO11-oligo-defined hotspot annotations ^36^. Given that hotspot usage is heterogeneous, only a subset of hotspots is engaged in any given nucleus. To maximize sensitivity, we focused on the hottest fraction of hotspots (top 10% by SPO11-oligo density ^36^) and quantified fold changes in ATAC-seq signal centered on these sites. This analysis revealed a reproducible increase in accessibility at hotspots in *Chd1^iMN^* spermatocytes, with the fold-change distribution shifted toward higher signal relative to controls (Figs. 5a and 5b). Consistently, aggregate ATAC-seq profiles showed an enhanced accessibility signature in *Chd1^iMN^* samples compared to controls (Fig. 5c).

Together, these data indicate that CHD1 restrains chromatin accessibility not only at a subset of active promoters but also at meiotic recombination hotspots, supporting the idea that CHD1 contributes to the chromatin environment in which crossover precursors mature. We note that chromatin accessibility at meiotic hotspots has been proposed to reduce significantly as spermatocytes transition from leptotene/zygotene into pachytene ^67^. Since the experimental samples used were primarily comprised of pachytene spermatocytes, we propose that CHD1 might be required for regulating chromatin accessibility as recombination intermediates mature, which would be consistent with its expression peaking at mid-late prophase I (Fig. 1c).

### CHD1 is required to stabilize pro-crossover assemblies in mid pachytene

We next asked whether the accessibility changes observed at hotspots, and the broader architectural defects described above, are accompanied by defects in the accumulation of pro-crossover factors at late recombination nodules. We first examined HEI10, a pro-crossover factor that concentrates at late recombination nodules in mid pachytene and promotes subsequent recruitment of MutLγ components ^68,69^. In control spermatocytes, HEI10 formed discrete foci along synapsed chromosomes in mid pachytene. In *Chd1^iMN^* spermatocytes, HEI10 foci were significantly reduced in number, and the remaining foci were noticeably smaller and dimmer (Figs. 5d-f), indicating impaired stabilization and/or maturation of HEI10-positive nodules.

Given the known functional relationship between HEI10 accumulation and MutLγ recruitment ^69^, we next assessed MLH1 localization. In control spermatocytes, MLH1 formed a typical set of prominent foci in mid-to-late pachytene. In *Chd1^iMN^* spermatocytes, MLH1 foci were significantly reduced in number, and their intensity and size was also decreased (Figs. 5g-i), consistent with a failure to robustly establish crossover-fated intermediates.

To test whether MLH1 positioning within the SC is also altered, we used STED microscopy to visualize MLH1 relative to the central element marker TEX12 ^70^. In controls, MLH1 foci were typically positioned close to the center of the TEX12-defined SC interior, as previously described ^71,72^. In *Chd1^iMN^* spermatocytes, the remaining - generally smaller - MLH1 foci were more frequently offset relative to the maximal TEX12 signal (Figs. 5j,k and Supplementary Figs. 5a-c), suggesting that CHD1 depletion not only reduces pro-crossover factor accumulation but also perturbs the precise positioning of crossover-associated assemblies within the SC. We note that, as for SYCE1, TEX12 signal was more continuous in *Chd1^iMN^* spermatocytes, indicating that the role of Chd1 in modulating the nanoscale organisation of the central region of the SC is not specific to SYCE1.

Together, these observations identify a specific defect in the stabilization and organization of late recombination nodules upon CHD1 depletion, providing a link between CHD1-dependent chromatin/axis phenotypes and the process of crossover maturation.

### CHD1 is dispensable for DSB formation and early DSB repair, but required for the assembly of crossover-specific complexes

The reduction in HEI10/MLH1 foci could in principle arise indirectly from fewer DSBs or defective early recombination steps, as is the case for *Spo11* hypomorphic mutants ^73^. To test this, we examined markers of break formation and early repair progression. First, we assessed REC114, a component of the meiotic DSB machinery that forms chromosome-associated foci in leptotene/zygotene prior to and during break formation ^74,75^. REC114 foci numbers and overall chromosome association were comparable between control and *Chd1^iMN^* spermatocytes (Supplementary Figs. 6a and 6b).

We next stained for RAD51, which accumulates at resected DSBs during leptotene and zygotene and declines as strand invasion and downstream processing proceed. RAD51 foci counts were indistinguishable between genotypes in leptotene and zygotene, and RAD51 foci declined with similar kinetics upon entry into pachytene (Figs. 6a and 6c).

We also examined γ-H2AX dynamics as an additional readout of DSB signaling and repair progression ^76-78^. In both control and *Chd1^iMN^* spermatocytes, γ-H2AX showed the expected transition from broad chromatin staining in early prophase to enrichment within the sex body in pachytene (Supplementary Fig. 6c). Phosphorylated RNAPII Ser2 was similarly excluded from the sex body in both genotypes (Supplementary Fig. 6c), consistent with typical MSCI-associated compartmentalization ^79-81^. Notably, discrete autosomal γ-H2AX “s-foci” (SPO11-dependent) were less readily detected in *Chd1^iMN^* nuclei ^82,83^ (Supplementary Fig. 6d), suggesting that the fine-scale organization of γ-H2AX on autosomes is altered even when its global dynamics appear intact. Because overall γ-H2AX signal intensity was comparable between genotypes (Supplementary Fig. 6e), this reduced focal detectability may reflect signal dilution across an expanded chromatin/nuclear area, analogous to the decrease in H3 foci density in *Chd1^iMN^* spermatocytes (Figs. 2i-k).

To further probe early recombination intermediate stability beyond RAD51 removal, we analyzed RPA2, which marks single stranded DNA-containing intermediates after strand invasion and persists into pachytene at a subset of sites ^72^. While RPA2 foci formed in comparable numbers during zygotene, SC-associated RPA2 foci were significantly reduced throughout early-to-late pachytene in *Chd1^iMN^* spermatocytes (Figs. 6b, d). We observed similar behavior for MSH4, a component of MutSγ that stabilizes early recombination intermediates ^84,85^ (Figs. 6e, g). Finally, RNF212, which helps maintain MutSγ -marked intermediates into mid prophase ^86-88^, was also decreased during pachytene in the mutant (Figs. 6f, h).

Together, these data indicate that CHD1 is largely dispensable for DSB formation and early RAD51-marked repair steps, but is required from early pachytene onward to maintain a robust population of stable SC-associated recombination intermediates and to support their maturation into HEI10/MLH1-positive crossover precursors.

### CHD1 associates with synapsed chromosomes via a direct interaction with SC central element protein SYCE1

To determine whether CHD1 might act directly within the SC environment, we examined its localization on meiotic chromosome spreads by immunofluorescence microscopy. In control spermatocytes, CHD1 appeared as discrete puncta and short stretches specifically aligned with the SC scaffold (Fig. 7a). CHD1 foci were rare in late zygotene, increased sharply upon entry into pachytene, peaked in mid pachytene, and declined again in late pachytene/diplotene (Fig. 7b and Supplementary Fig. 7a). Importantly, the few CHD1 foci detected in late zygotene or diplotene were consistently restricted to regions where homolog axes were synapsed, suggesting that CHD1 association is synapsis-dependent. Consistent with this, CHD1 signal was largely absent from the sex body except within the pseudoautosomal region (PAR), the only segment of stable X-Y synapsis (white arrows: Fig. 7a).

Because the conditional *Chd1* knockout might be mosaic at the single-cell level, we verified the specificity of the immunostaining by examining *Chd1^iMN^* spreads, where the majority of nuclei lacked detectable CHD1 SC-associated signal (Fig. 7b and Supplementary Fig. 7a). Approximately 10% of nuclei assessed retained wild-type levels of CHD1 signal, consistent with incomplete depletion, plausibly explaining the small fraction of meiocytes that enter metaphase I in *Chd1^iMN^* mutants (Figs. 1i and 1j). We defined a cut-off point for CHD1 foci as an exclusion criterion for nuclei with poor CHD1 depletion (see black triangles in Fig. 7b). This has been used where possible in additional analyses of meiotic chromosome spreads from the *Chd1^iMN^* mutant (see Methods for further details).

We next asked whether the SC-associated localization of CHD1 is sex-specific. In spreads from embryonic (E16.5) oocytes, CHD1 likewise formed abundant SC-associated foci, with stage-dependent dynamics similar to those observed in spermatocytes (Supplementary Figs. 7b and 7c), indicating that the meiotic SC association of CHD1 is not restricted to male meiosis.

To test synapsis dependence directly, we examined CHD1 localization in *Sycp1^−/−^* spermatocytes, which form axial elements and align homologs but fail to assemble the mature SC central region ^21^. In *Sycp1^−/−^* mutants, CHD1 foci were not detected on chromosomes despite extensive aligned axes in pachytene-like spreads (Figs. 7c and 7d), indicating that CHD1 recruitment requires synapsis/central region assembly rather than axis formation per se.

To characterize the position of CHD1 within the SC at higher resolution, we performed STED microscopy. CHD1 puncta localized within the space between paired SYCP3 tracks, consistent with association near the synapsed interface (Figs. 7e and 7f). Co-staining with the central element protein SYCE1 further showed that SYCE1 forms a comparatively continuous central element signal, whereas CHD1 puncta occupy a subset of SYCE1-positive segments (Fig. 7g), suggesting that CHD1 is recruited by - but does not exactly mirror - central element localization. We further note that the width of the synaptonemal complex was not altered when comparing segments positive or negative for CHD1 foci (Fig. 7f). This finding, alongside our reports of mostly normal synapsis in *Chd1^iMN^* mutants (Fig. 2a), suggests that despite its synapsis-dependent localisation, CHD1 itself is not per se required for synapsis.

To investigate how CHD1 might be recruited to the SC, we sought to identify interacting proteins. CHD1 immunoprecipitation from adult testis extracts followed by mass spectrometry reproducibly enriched central element proteins, with SYCE1 as a prominent interactor, along with SYCE1L and SIX6OS1 (a known direct interaction partner of SYCE1 ^89,90^) (Fig. 7h; Supplementary Table 4). Interactions with central element components were also detected in immunoprecipitates from STA-PUT-enriched spermatocyte fractions, arguing that they occur specifically in prophase I (Supplementary Fig. 7d, Supplementary Table 5). Importantly, a direct CHD1-SYCE1 interaction was validated by in vitro pulldown assays using purified proteins (Fig. 7i), supporting the idea that CHD1 is recruited to synapsed chromosomes through a direct interaction with SYCE1.

Finally, we asked whether SYCE1 binding interferes with the enzymatic activity of CHD1 in a restriction enzyme accessibility-based nucleosome remodeling assay ^91^. As expected, purified CHD1 was able to shift end-positioned nucleosomes, increasing accessibility to the DpnII site over time (Supplementary Figs. 7e-g). Addition of SYCE1 did not cause a discernible difference in remodeling efficiency and kinetics. These data support a model in which CHD1 is recruited to the SC during meiotic prophase I and remains enzymatically active whilst present at the interface between recombining chromosomes.

### SC-associated CHD1 partially colocalizes with a subset of recombination markers

Given the requirement for CHD1 in maintaining stable pachytene recombination intermediates and promoting late nodule maturation, we asked whether CHD1 physically associates with recombination sites within the synapsed SC environment. A comparison of stage dynamics already suggested that CHD1 is unlikely to behave as a constitutive component of early intermediates: SC-associated CHD1 foci rise sharply from early to mid pachytene, whereas the abundance of SC-associated RPA2 foci declines over the same interval (Figs. 7j and 7k). Consistent with this temporal offset, co-staining of CHD1 with RPA2 under conventional resolution revealed a modest overall degree of overlap (Fig. 7j).

To define these spatial relationships more precisely, we performed STED imaging and quantified the overlap between CHD1 puncta and recombination markers. In early pachytene, when CHD1 and RPA2 foci were present at comparable overall densities, RPA2 foci exhibited heterogeneous morphologies along the SC. Approximately one-third of RPA2 foci showed partial overlap or close apposition with a CHD1 focus, independent of RPA2 focus size (Figs. 7l and 7m). In most cases, CHD1 puncta were positioned adjacent to one edge of an RPA2 focus or coincided with only a small subregion of larger RPA2 assemblies. This pattern suggests that CHD1 is frequently in close proximity to RPA2-marked recombination intermediates within the confined SC central compartment, rather than stably co-occupying these structures in a strict one-to-one manner. The size independence of this association further raises the possibility that CHD1 engages dynamic recombination intermediates coated with variable amounts of RPA2.

To better understand whether CHD1 might affect recombination structures in the absence of the synaptonemal complex and expand upon our prior observation that RPA2-marked intermediates show altered repair dynamics upon conditional *Chd1* deletion (Figs. 6b and 6d), we next generated double-knockout mice strains where *Chd1* was conditionally deleted in a *Sycp1−/−* background. As is the case in *Sycp1−/−* mice ^21 92^, both *control* and *Chd1^iMN^* mice with additional deletion of *Sycp1* showed numerous bridge-like RPA2 foci linking closely aligned, but non-synapsed homologs (Fig. 7n, compare left and right panels). After again confirming that, in the absence of synapsis, CHD1 foci do not associate with the synaptonemal complex (Fig. 7o), we quantified RPA-bridges in each genotype and found them to be unchanged (Fig. 7p).

As CHD1 depletion most strongly compromises late recombination nodules, we assessed CHD1 relative to MLH1 in mid-to-late pachytene. At these stages, a substantial fraction (~60%) of MLH1 foci were found to at least partially overlap with CHD1, but complete colocalization remained uncommon (Figs. 7q and 7r). Importantly, CHD1 occupies a larger fraction of the SC in mid pachytene than in earlier stages, so the apparent increase in CHD1-MLH1 association likely reflects both genuine proximity and increased CHD1 coverage along synapsed chromosomes.

Together, these data indicate that CHD1 resides within the same SC central region compartment that hosts maturing recombination intermediates, and is frequently positioned adjacent to, or overlapping with, recombination-associated assemblies. However, rather than an obligatory structural component of recombination nodules, CHD1 appears to act as a chromatin remodeler positioned at the interface between synapsed homologs, poised to modulate the local chromatin environment at a subset of recombination sites as they mature through pachytene. Given the rapid turnover and stage-specific maturation of recombination intermediates, limited steady-state colocalization in fixed spreads is also compatible with a transient or sequential relationship between CHD1 and RPA-containing recombination assemblies.

### CHD1 recruitment to the SC is modulated by the crossover maturation factors RNF212 and HEI10

Given the requirement for CHD1 in promoting the assembly of late recombination nodules, we asked whether CHD1 recruitment to the SC depends on meiotic recombination initiation or on formation of crossover-designated intermediates. In mutants defective for DSB formation (e.g., *Spo11^−/−^, TopoVIBL^−/−^*), synapsis is severely perturbed, but residual non-homologous synapsis can occur and is accompanied by central element assembly between improperly paired axes ^7,93,94^. In *Spo11^−/−^* mutants, CHD1 foci were detected specifically in the synapsed segments, despite the absence of programmed DSB formation (Supplementary Fig. 7h), indicating that synapsis/central element assembly is sufficient for CHD1 recruitment. We next examined CHD1 signal in mutants defective in crossover maturation (*Rnf212^−/−^*, *Hei10^mei4/mei4^*, and *Prr19^−/ −^* ^69,86,95^). In each case, CHD1 remained at least partly associated with synapsed axes (Supplementary Figs. 7h and 7j), although its abundance varied between genotypes. Notably, CHD1 signal was reduced in *Rnf212^−/−^* and *Hei10^mei4/mei4^* backgrounds but less affected in *Prr19^−/−^*,(Supplementary Figs. 7i and 7k) suggesting that SUMO/ubiquitin-dependent pro-crossover regulatory pathways can influence CHD1 accumulation without being strictly required for its recruitment.

## DISCUSSION

Our study identifies CHD1 as a key regulator of meiotic chromosome organization that supports homolog engagement and genetic exchange during meiotic prophase I. More specifically, our findings suggest that CHD1-dependent chromatin remodeling shapes chromosome organization across scales, helping to establish a chromosome state that is permissive for chromosome compaction, homolog synapsis and recombination nodule maturation.

A key implication of our work is that chromatin remodeling contributes to meiotic recombination beyond the control of DSB formation. In mammals, considerable attention has focused on how PRDM9 binding and the chromatin environment of recombination hotspots, including nucleosome positioning and histone modifications at these sites, define where meiotic DSBs occur ^33-35,38,96-100^. However, DSB formation is only the entry point into a prolonged recombination program and CHD1 is dispensable for these initial steps. Upon conditional deletion of *Chd1* in the germline, prominent defects emerge during pachytene, affecting the accumulation and/or maintenance of recombination factors with essential roles in crossover maturation. Thus, CHD1 appears to remodel chromatin to create an environment that permits the stabilization of crossover precursors, rather than determining where recombination is initiated (Fig. 8). This late recombination function is broadly consistent with our previous work in budding yeast, where Chd1 was identified as a meiosis-specific interactor of the MutLγ-Exo1 crossover machinery and shown to be required for efficient crossing-over. Importantly, both its ATPase activity and chromodomain were required for this function, arguing that crossover control depends on canonical chromatin remodeling ^66^. This evolutionary convergence suggests that nucleosome remodeling is functionally integrated into crossover maturation itself.

The chromatin defects caused by CHD1 depletion are not consistent with a simple loss of nucleosomes. Our data instead point to altered nucleosome organization and higher-order chromatin architecture, which then gives the appearance of chromatin decompaction nucleus-wide. Importantly, we observed a regional element to this decompaction. Accessibility increases are particularly prominent at active promoters, across broad regions of the sex chromosomes and at meiotic recombination hotspots. DNA FISH further indicates that loci gaining accessibility move farther from chromosome axes, whereas loci with stable accessibility do not. Thus, CHD1 restricts local chromatin expansion at selected genomic regions, helping maintain compact chromatin organization around meiotic chromosome axes. The known biochemical activity of CHD1 provides an attractive mechanistic framework for how loss of CHD1 might lead to chromatin decompaction. Higher-order chromatin organization has been shown to be highly sensitive to nucleosome architecture, including nucleosome positioning, spacing and linker DNA geometry ^42,101^. Relatively subtle changes in nucleosome spacing can alter the balance between intra- and intermolecular nucleosome interactions, thereby influencing chromatin compaction and phase separation ^102^. Likewise, classical reconstitution studies demonstrated that nucleosome repeat length and linker DNA geometry profoundly influence higher-order chromatin folding ^103,104^. We therefore propose a model whereby CHD1, in its capacity as an ATP-dependent chromatin remodeler, contributes towards meiotic chromatin loop compaction by modulating nucleosome positioning and fiber organization (Figs. 8a and 8b). A non-mutually exclusive possibility is that CHD1-mediated nucleosome positioning modulates the capacity of chromosome-organizing complexes, including condensins, to compact meiotic chromosomes. Both scenarios could help explain how CHD1 promotes chromatin compaction without modulating chromosome axis length.

The additional observation of an SC phenotype reveals a second level of chromosome organization influenced by CHD1. CHD1 depletion narrows the SYCP3/SYCP1-defined SC compartment. How then might a chromatin remodeler influence SC architecture? Chromatin loops emerge directly from chromosome axes and remain intimately associated with the SC throughout pachytene ^17,60,105,106^. If CHD1 establishes chromatin fibers with appropriate mechanical properties (Fig. 8a), altered chromatin organization could modify the physical constraints experienced by the SC. The coiled-coil architecture of SYCP1 and the organization of central-element proteins may provide sufficient mechanical compliance to accommodate these altered constraints without disrupting homolog synapsis ^107,108^. In this model, chromatin loops expand away from chromosome axes while the lateral-element compartment becomes compressed relative to the central region, explaining how chromosome decompaction and SC narrowing can coexist (Fig. 8b). Our findings therefore support a view of the SC as a chromatin-embedded structure whose nanoscale organization is shaped by the surrounding chromatin environment.

The localization pattern of CHD1 provides a direct mechanism for reshaping the chromatin environment according to the specialized needs of meiocytes undergoing meiotic recombination. CHD1 accumulates as puncta between synapsed homologs, interacts directly with SYCE1 and requires central-element assembly for robust chromosomal localization. This positions CHD1 within the same chromosomal compartment that hosts maturing recombination intermediates ^1^. Nevertheless, CHD1 is unlikely to represent an obligate structural component of recombination nodules themselves. Its overlap with RPA2 and MLH1 is partial and stage-dependent, consistent with a transient or locally restricted relationship. We therefore favor a model in which CHD1 remodels chromatin adjacent to selected recombination sites, thereby influencing the local chromosome environment in which recombination intermediates acquire late markers.

This model also reconciles the temporal dynamics of CHD1 function. CHD1-associated chromatin defects are already evident before CHD1 reaches maximal enrichment along synapsed chromosomes, suggesting that CHD1 may act through canonical chromatin-binding mechanisms earlier in prophase. H3K4me3-marked promoters and PRDM9-dependent hotspots are plausible early chromatin contexts for CHD1 action ^42,109,110^. However, hotspot-associated H3K4me3 declines by early pachytene ^111,112^, whereas CHD1 accumulation peaks later. SC-dependent recruitment through SYCE1 may therefore provide a second, meiosis-specific targeting mechanism that enriches or stabilizes CHD1 at synapsed chromosomes after the early hotspot-marking phase has waned. In this two-layered model, CHD1 first contributes to chromatin organization through conventional chromatin engagement and is later repositioned or stabilized within the SC environment to support chromosome architecture and late recombination-nodule assembly.

CHD1 accumulation at the SC is also influenced by recombination-nodule maturation factors. CHD1 remains detectable on synapsed chromosomes in Spo11 mutants, indicating that DSB formation is not strictly required for recruitment. However, CHD1 signal is reduced in *Rnf212* and *Hei10* mutants, suggesting that pro-recombination SUMO/ubiquitin pathways modulate its accumulation. These observations place CHD1 within the late recombination environment without making it simply downstream of recombination initiation. Whether reduced CHD1 accumulation contributes directly to the recombination defects of *Rnf212* and *Hei10* mutants remains an important question. More generally, these findings suggest that recombination regulators may influence not only HR proteins themselves, but also the chromatin-remodeling activities that shape the local environment in which recombination intermediates acquire late markers.

In summary, our findings expand the role of chromatin remodeling during meiosis. Rather than acting primarily through hotspot activation or transcriptional regulation, CHD1 organizes meiotic chromatin during the prolonged period in which recombination intermediates mature within synapsed chromosomes. We propose that CHD1 establishes nucleosome architecture that gives meiotic chromatin the physical properties required for chromosome compaction, and that meiotic progression may retarget this remodeling activity to recombination intermediates embedded in the central region of the SC. In this framework, the SC is not merely a scaffold for recombination proteins, but also a platform that directs local chromatin remodeling during late recombination-nodule assembly. More generally, our work suggests that chromosome architecture, chromatin mechanics and genetic exchange are coordinated through dynamic nucleosome remodeling.

## METHODS

### Mouse strains and breeding strategy

All mice used in this study were maintained on a C57BL/6J background unless otherwise indicated. Animals were housed under specific pathogen-free conditions at the Max Perutz Labs animal facility. Experiments performed in Vienna were conducted in accordance with protocols approved by the Austrian Ministry of Science and Communications/Austrian Ministry of Education, Science and Research (licence number 2021-0.478.372). Additional experiments involving *Spo11*^−/−^, *Hei10^mei4/mei4^* and *Rnf212*^−/−^ mice were performed in the laboratory of Neil Hunter at the University of California, Davis, USA. Unless otherwise stated, meiotic chromosome spreads from spermatocytes were prepared from adult males aged P40–P80, and oocyte spreads were prepared from E16.5 embryos.

Mouse lines generated or maintained in this study are available from the corresponding author upon reasonable request and subject to institutional and regulatory approval.

Cryopreserved embryos carrying the conditional *Chd1* allele, *Chd1tm1c(KOMP)Rsan*, were revived from the Mutant Mouse Resource & Research Center (MMRRC). This allele contains loxP sites flanking exon 16 of *Chd1* and has been described previously ^42,44^.

*Mvh-CreERT2* mice were received as a gift from Philipp Kaldis and have been described previously ^113^. *Chd1^+/flox^* mice were crossed with *Mvh-CreERT2* mice and subsequently intercrossed to generate mice homozygous for the floxed *Chd1* allele and carrying the *Mvh-CreERT2* transgene. These mice are referred to throughout the manuscript as *Chd1* inducible meiotic-null mice (*Chd1^iMN^*).

For inducible whole-body deletion, *Chd1flox/flox* mice were crossed with *Rosa26-CreERT2* mice, received as a gift from Martin Leeb (Max Perutz Labs, Vienna) and previously described ^114^. Intercrossing was used to generate *Chd1flox/flox; Rosa26-CreERT2* mice, referred to as *Chd1* inducible knockout mice (*Chd1^iKO^*). This line was used for derivation of inducible knockout MEFs.

*Sycp1*^+/−^ and *Prr19^+/−^* mice were received as gifts from Attila Toth (TU Dresden) and were bred to homozygosity in-house. Attempts to generate constitutive meiocyte-specific *Chd1* mutant lines using *Spo11-GFP-Cre* or *iStra8-Cre* resulted in only limited depletion of CHD1 protein and were not pursued further.

Genotyping was performed using genomic DNA isolated by HotSHOT lysis from neonatal toe biopsies collected at postnatal days 2–5 ^115^. Primer sequences and PCR conditions used for genotyping are provided in Supplementary Table 6.

### Tamoxifen-mediated induction of Cre recombinase in vivo

For induction of recombination in *Chd1^iMN^* mice, tamoxifen (Sigma, T5648) was dissolved in corn oil (Sigma, C8267) at 16.6 mg/mL and administered by intraperitoneal injection at 150 μl per dose, corresponding to 2.5 mg tamoxifen. Mice received one injection per day for six consecutive days, with the first injection designated day 0. This regimen was based on previously described *CreERT2* induction approaches ^116^.

### Mouse embryonic fibroblast isolation and culture

MEFs were isolated from E14.5 embryos and cultured as previously described ^117^. Embryos were mechanically dissociated and plated in DMEM supplemented with 10% fetal bovine serum and 1% penicillin–streptomycin. Cells were maintained at 37 °C in a humidified incubator with 5% CO_2_ and passaged upon reaching confluency. Only MEFs maintained for four passages or fewer were used for experiments.

### Inducible Chd1 deletion in MEFs

MEFs were derived from *Chd1^iKO^* embryos. To induce recombination, cells were treated with 4-hydroxytamoxifen (4-OHT; Merck, SML1666) at a final concentration of 1 μM for 24 h, consistent with previously reported conditions ^118^. Recombination efficiency was assessed by genotyping.

### Meiotic chromosome spread preparation

Meiotic chromosome spreads from spermatocytes were prepared from P40–P80 adult males using a previously described drying-down method with minor modifications ^119^.

Seminiferous tubules were dissected from euthanized mice and rinsed once in PBS (Gibco, 14190-094). For each pair of testes, seminiferous tubules were incubated in 1 ml hypotonic extraction buffer containing 30 mM Tris-HCl, 50 mM sucrose, 17 mM trisodium citrate dihydrate and 5 mM EDTA, adjusted to pH 8.2, for 30 min. During incubation, tubules were gently separated using forceps.

Tubules were subsequently transferred to 500 μl of 100 mM sucrose solution, pH 8.2, and incubated for 40 min. After 10 min, tubules were fragmented using sharp forceps and pipetted 20 times through a cut 1-ml pipette tip. After 30 min, the suspension was pipetted 20 times through an uncut 1-ml pipette tip. The resulting suspension was passed twice through a 100-μm cell strainer, once through a 70-μm cell strainer and once through a 40-μm cell strainer. The final volume was adjusted to 1 ml with sucrose solution.

Aliquots of 20–30 μl were applied to Superfrost Plus microscopy slides (Epredia, J1800AMNZ) pre-coated with fixative containing 1% paraformaldehyde and 0.15% Triton X-100, adjusted to pH 9.2 and filtered through a 0.2-μm membrane. Samples were distributed across the slide by tilting and incubated horizontally in a humid chamber at 37 °C for 2 h. Slides were then partially dried at room temperature for 10 min and either used immediately or stored at −80 °C.

Meiotic chromosome spreads from oocytes were prepared from ovaries isolated from E16.5 female embryos as described previously ^120^. The procedure was identical to that used for spermatocyte chromosome spreads, except that the volumes of hypotonic extraction buffer and sucrose solution were reduced to 200 μl and 60 μl, respectively.

### Immunofluorescence staining of meiotic chromosome spreads

Chromosome spreads were washed once with PBS and a hydrophobic barrier was drawn around the sample using an A-PAP pen (Merck, Z377821) without allowing the slide to dry. Slides were blocked for 30 min at room temperature in buffer containing 10% BSA, 3% milk powder and 0.15% Triton X-100 in distilled water. For staining with MSH4 antibodies, blocking buffer containing 0.5% BSA and 0.5% milk powder was used.

Primary antibodies were diluted in the corresponding blocking buffer and incubated for 16 h at 4 °C in a humid chamber. Slides were washed three times for 10 min in PBST (PBS containing 0.05% Tween-20), followed by incubation with secondary antibodies diluted in blocking buffer for 45 min at room temperature. Slides were subsequently washed three times for 10 min in PBST and rinsed in PBS.

DNA was counterstained with Hoechst 33342 (Invitrogen, H3570; 1:40,000 in distilled water) for 6 min at room temperature. Slides were rinsed twice in PBS and mounted using antifade aqueous mounting medium (Abcam, Ab104135).

Widefield images were acquired using a Zeiss Axio Imager M2 upright microscope equipped with an EC Plan-Neofluar 100×/1.3 Oil M27 objective and operated using ZEN Blue software. Antibodies and reagents used in this study are listed in Supplementary Table 6.

### STED super-resolution microscopy

For STED imaging, chromosome spreads were stained as described above with the following modifications. Secondary antibody incubation was extended to 4 h at room temperature. DNA was counterstained with SYBR Gold where indicated, and coverslips were mounted using ProLong Glass antifade medium and cured for at least 16 h before imaging.

Images were acquired using a Zeiss Axio Imager.A2 microscope equipped with a STEDyCON system and a 100×/1.46 oil objective. Images were deconvolved using Huygens Professional software. In selected experiments, SYCP3 was acquired as an additional confocal channel alongside two super-resolution channels.

### Analysis of SC continuity and width in STED images

Individual synaptonemal complexes were manually annotated using custom Fiji macros, and straightened fluorescence-intensity profiles were extracted from paired image channels. Profiles were subsequently analyzed in R using a custom pipeline implementing adaptive peak-window detection, two-Gaussian fitting, profile alignment and quality-control filtering.

Peak positions, inter-peak distances, signal widths and fluorescence intensities were calculated for each profile. Quality-control thresholds were derived from wild-type datasets and applied consistently across experimental conditions. Profiles passing quality control were aligned to the midpoint between the left and right axial-element peaks and averaged to generate chromosome-, nucleus- and genotype-level summary profiles.

### Analysis of MLH1 foci in STED images

STED images of MLH1 foci were analyzed using Huygens Professional software. The Huygens slicer tool was used to generate 600-nm line profiles bisecting the MLH1 focus of interest from the point furthest from the central-element signal marked by TEX12, generating fluorescence-intensity measurements at 20-nm intervals across the profile. Resulting line-trace data were exported and aligned to the maximal TEX12 signal. Owing to the asymmetric positioning of MLH1 signals relative to TEX12, profile directionality was standardized such that, for each line trace, the point of the MLH1 signal furthest from TEX12 was oriented to the left side of the profile. Profiles were subsequently averaged to generate nucleus- and genotype-level summary profiles.

### Immunofluorescence combined with whole-chromosome FISH

Chromosome spreads were blocked and stained with anti-SYCP3 antibody (Santa Cruz, Sc-74569; 1:1,000) for 30 min, followed by washing, secondary-antibody incubation for 45 min and further washing as described above.

Slides were denatured sequentially in: 2× SSC for 30 min at 70 °C followed by 20 min cooling to room temperature; 0.1× SSC for 1 min at room temperature; 0.07 N NaOH for 1 min at room temperature; 0.1× SSC for 1 min at 4 °C; 2× SSC for 1 min at 4 °C; 70% ethanol for 1 min at room temperature; and two successive 1-min incubations in 95% ethanol. SSC solutions were prepared by dilution of 20× SSC (Invitrogen, AM9763) and adjusted to pH 7.

Following ethanol dehydration, slides were air-dried and approximately 20 μl undiluted MetaSystems FISH probe, denatured immediately before use, was applied beneath an 18 × 18-mm coverslip. Probes were hybridized for 24 h at 37 °C in a humidified light-protected chamber. Probes were denatured according to the manufacturer’s instructions at 75 °C for 5 min, 10 °C for 30 s and 37 °C for 30 min.

Post-hybridization washes consisted of 0.4× SSC for 2 min at 72 °C followed by 2× SSC for 2 min at room temperature. Slides were counterstained with Hoechst 33342 and mounted as described above. Widefield images were acquired using a Zeiss Axio Imager M2.

### Immunofluorescence combined with fosmid DNA FISH

Fosmid staining was adapted from ^58^. Chromosome-spread slides were incubated in 1 M NaSCN for 30 min at 70 °C, washed in 1× SSC and digested with 100 μg/mL RNase A for 1 h at 37 °C. Slides were washed in 1× SSC, covered with 70% formamide/30% 2× SSC and denatured at 85 °C for 5 min before transfer to ice-cold 2× SSC to prevent renaturation.

Probes in hybridization buffer were applied beneath a coverslip, sealed with rubber cement and incubated for 12 h at 42 °C in a humid chamber. Slides were washed three times for 5 min in 0.05× SSC at 42 °C and subsequently processed for immunostaining.

Three consecutive fosmids were used to label each genomic region. For chromosome 2, the region with unchanged accessibility was labelled using WIBR1-2473G14, WIBR1-0132I08 and WIBR1-0744O09, whereas the region with increased accessibility was labelled using WIBR1-1100H23, WIBR1-0881K09 and WIBR1-2607E19. For chromosome 17, WIBR1-0501A19, WIBR1-2526E06 and WIBR1-0119I02 labelled the unchanged region, and WIBR1-1995J21, WIBR1-0437N20 and WIBR1-2512A07 labelled the increased-accessibility region. For chromosome X, WIBR1-1482I02, WIBR1-1993H01 and WIBR1-1291N07 labelled one region, and WIBR1-1612E01, WIBR1-2614B08 and WIBR1-2583A21 labelled the second region.

Fosmids were amplified in LB medium supplemented with 25 μg/mL chloramphenicol and purified using a Maxi-prep kit (Macherey Nagel). For labelling, 1 μg of each fosmid was used. Groups of three fosmids corresponding to each genomic region were labelled using the FISH Tag DNA Multicolor Kit, Alexa Fluor dye combination (Invitrogen, F32951), according to the manufacturer’s instructions.

For each slide, 40 ng labelled probe, 10 μg salmon sperm DNA (Invitrogen, 15632011) and 5 μg mouse Cot-1 DNA (Invitrogen, 18440016) were precipitated in 200 μl ethanol containing 0.096 M sodium acetate and washed with 70% ethanol. Pellets were resuspended in 5 μl 100% formamide at 37 °C, denatured for 7 min at 75 °C and incubated for 1 h at 37 °C. Subsequently, 4 μl 20× SSC and 6 μl 50% dextran were added.

Images were acquired using a Zeiss Axio Imager Z2 Apotome equipped with a 63× Plan-Apochromat 1.46 NA oil objective and an ORCA-Flash4 LT monochrome camera (Hamamatsu; 2048 × 2048 pixels; 6.5-μm pixel size).

### Analysis of fosmid FISH signal extension

FISH measurements were performed on non-deconvolved images using ImageJ. Fosmid-probe extension relative to the synaptonemal complex was measured using the freehand-line tool. For each contiguous probe signal, a line was traced from the point closest to the SYCP3-positive SC axis to the most distal point within the same signal domain. Where signal was clearly present on both sides of the SC, each side was measured independently.

FISH signal-extension measurements were analyzed using GraphPad Prism 10. Comparisons between genotypes were performed using two-sided Mann–Whitney U tests. Measurements were obtained from two independent mice per genotype.

### Histology

Testes were fixed in modified Davidson’s solution for either 10 h at room temperature or overnight at 4 °C and subsequently transferred to 70% ethanol. Samples were processed, paraffin embedded, sectioned at 5 μm and mounted on Superfrost Plus slides.

For hematoxylin and eosin staining, sections were deparaffinized, rehydrated and stained using an automated slide stainer. Coverslips were mounted with Eukitt mounting medium. Whole-slide images were acquired using an Olympus VS120 slide scanner equipped with a 40× oil-immersion objective.

### Immunofluorescence staining of tissue sections

Sections were deparaffinized and rehydrated by two sequential incubations in n-butyl acetate (Thermo Scientific, A19412.AP), two in 96% ethanol, one in 50% ethanol and one in distilled water, for 1 min each.

Antigen retrieval was performed in 60 mM trisodium citrate containing 0.05% Tween-20, adjusted to pH 6, for 45 min at 95 °C. Blocking, primary-antibody incubation, secondary-antibody incubation and nuclear counterstaining were subsequently performed as described above for meiotic chromosome spreads.

### TUNEL assay

Apoptotic cells were detected in deparaffinized and rehydrated tissue sections using the ApopTag Red In Situ Apoptosis Detection Kit (Merck, S7165) according to the manufacturer’s instructions. Following TUNEL labelling, sections were stained with the indicated antibodies and imaged using an Olympus VS120 slide scanner.

### STA-PUT fractionation

STA-PUT fractionation was performed with minor modifications to a previously described protocol ^45^. Seminiferous tubules were dissected from euthanized mice, rinsed in PBS and finely minced in 1 ml pre-warmed DMEM. Tissue was dissociated by 30 passages through a 1-ml pipette tip. DMEM was increased to 5 ml and supplemented with 0.5 mg/mL collagenase type VI and 12 μg/mL DNase I, followed by incubation for 15 min at 37 °C.

Tubule fragments were pelleted at 600 × g for 5 min, resuspended in 5 ml 0.25% trypsin in PBS and incubated for a further 20 min at 37 °C. 5mL of 0.5% BSA in DMEM was subsequently added. The resulting suspension was passed twice through a 100-μm cell strainer and centrifuged at 600 × g for 5 min. Cells were washed once in PBS.

Cells isolated from the testes of two mice were pooled and resuspended in 10 ml DMEM. The heterogeneous suspension was separated by gravity sedimentation through a 2–4% BSA gradient for 3.5 h. Sequential 10-ml fractions were collected, and fraction F2 was used for downstream experiments.

### Validation of STA-PUT fractions

The cellular composition of F2 fractions was assessed by preparing chromosome spreads from approximately 50,000 fractionated cells and staining for SYCP3 and TEX12. Relative abundances of meiotic substages were determined using established cytological criteria.

### Meiotic metaphase spreads

Seminiferous tubules were digested as described for STA-PUT sample preparation, without subsequent fractionation. Cells were resuspended in 100 μl PBS and brought gradually to 5 ml by dropwise addition of pre-warmed 75 mM KCl. Samples were incubated for 15 min at 37 °C. Five milliliters ice-cold methanol:glacial acetic acid (3:1) was added dropwise, and cells were pelleted at 160 × g for 5 min. Pellets were resuspended in 100 μl fixative, diluted dropwise with a further 5 ml fixative and incubated on ice for 15 min. Following two additional rounds of centrifugation and fixation, the final pellet was resuspended in 200 μl fixative.

Aliquots of 20 μl were dropped from approximately 10 cm onto Superfrost Plus microscopy slides. Slides were dried for 2 min on a metal block heated to 50 °C and stored at −20 °C.

For imaging, slides were stained with Hoechst 33342 (1:40,000) for 4 min at room temperature, rinsed twice in PBS and mounted with antifade aqueous mounting medium. Widefield images were acquired using a Zeiss Axio Imager M2. More than 100 tiled images were collected per sample-to-sample slide areas containing chromosome spreads without prior selection. Higher-magnification images of metaphase cells were acquired using an EC Plan-Neofluar 100×/1.3 Oil M27 objective.

### Live-cell imaging of STA-PUT-enriched spermatocytes

Following STA-PUT isolation, cell number and viability were determined using a Countess 3 automated cell counter (Thermo Fisher Scientific, A49891). Two million cells were washed in PBS, pelleted at 600 × g for 5 min and resuspended in 2 ml spermatocyte culture medium consisting of MEM-α supplemented with 10 mM sodium lactate, 5% FBS, 25 mM HEPES and 1% penicillin–streptomycin. Twelve-well plates were coated for 10 min with 2 mg/mL concanavalin A in PBS supplemented with 50 mM MnCl_2_ and washed twice with PBS. Cells were allowed to adhere for 10 min at 32 °C under 5% CO_2_. Hoechst 33342 was then added directly to a final dilution of 1:10,000. Cells were imaged immediately using a Zeiss Axio Observer Z1 inverted fluorescence microscope equipped with a 40× air objective (NA 0.75) and operated using ZEN Blue software. Samples were imaged through the cell-culture plate while maintained at 32 °C and 5% CO2.

### Acid extraction of histones

Histones were acid extracted from STA-PUT-enriched spermatocyte fractions using a previously described approach with minor modifications ^121^. F2 fractions containing 2 × 10^6 cells were washed in PBS and incubated for 30 min at 4 °C under continuous rotation in 5 ml hypotonic lysis buffer containing 10 mM Tris-HCl, 1 mM KCl, 1.5 mM MgCl_2_, 1 mM DTT and 1 μM Na_3_VO_4_ supplemented with cOmplete Protease Inhibitor. Nuclei were pelleted at 4,500 × g for 10 min at 4 °C. Following repeated mechanical homogenization using a disposable pestle, nuclei were resuspended in 400 μl 0.4 N H2SO_4_ and incubated overnight at 4 °C with continuous rotation.

After removal of nuclear debris, acid-extracted proteins were precipitated for 30 min on ice using trichloroacetic acid at a final concentration of 33%. Proteins were pelleted at 21,000 × g for 15 min at 4 °C, washed twice with ice-cold acetone, air-dried and resuspended in 10 μl biotechnology-grade water. 8-μl were snap-frozen for mass-spectrometry analysis. The remaining material was analyzed by SDS–PAGE and Coomassie staining to confirm histone enrichment.

### RNA-seq analysis

Total RNA was isolated from STA-PUT-enriched F2 fractions. One million cells were resuspended in 1 ml PBS. A 100-μl aliquot was removed and snap-frozen for subsequent analysis of CHD1 protein levels. The remaining cells were pelleted and resuspended in 500 μl TRI Reagent (Sigma-Aldrich, T9424).

RNA was extracted by sequential chloroform separation and isopropanol precipitation. Following DNase I treatment, RNA was further purified by phenol:chloroform:isoamyl alcohol extraction and ethanol precipitation and resuspended in TE buffer.

Stranded mRNA-seq libraries were prepared using poly(A) enrichment, fragmentation, cDNA synthesis, adapter ligation, indexing and PCR amplification.

RNA-seq data were processed using the nf-core/rnaseq pipeline implemented in Nextflow^122^. Adapter trimming was performed using Trim Galore!/Cutadapt ^123^. Reads mapping to abundant RNA species, including rRNA, were removed using Bowtie2 ^124^, and filtered reads were aligned to the mouse reference genome (Ensembl GRCm38/mm10) using STAR ^125^. Gene-level counts were generated using featureCounts ^126^. Differential gene-expression analysis was performed using DESeq2 ^127^, using at least three biological replicates per genotype. Genes with Benjamini–Hochberg-adjusted P values <0.01 (FDR <1%) were considered differentially expressed.

### ATAC-seq analysis

Two sets of ATAC-seq libraries were generated, from meiotic and somatic samples. Meiotic libraries were prepared from STA-PUT-purified spermatocyte F2 fractions, with three biological replicates per condition. Somatic libraries were generated from *Chd1^iKO^* MEFs treated with either 4-OHT or ethanol, with three replicate libraries per condition. All ATAC-seq datasets were processed using the same analysis pipeline.

For spermatocyte ATAC-seq, cell pellets were washed in PBS and 40,000 cells per sample were processed using the Active Motif ATAC-Seq Kit according to the manufacturer’s instructions. For MEF ATAC-seq, 4-OHT- or ethanol-treated *Chd1^iKO^* MEFs were trypsinized at approximately 70% confluency, washed in PBS, and 100,000 cells per sample were processed using the Active Motif ATAC-Seq Kit according to the manufacturer’s instructions. Libraries were sequenced on an Illumina NextSeq 550 using paired-end 75-bp reads. ATAC-seq data were processed using the nf-core/atacseq pipeline implemented in Nextflow ^122^. Adapter trimming and quality filtering were performed using Trim Galore!/Cutadapt ^123^. Reads were aligned to GRCm38/mm10 using BWA-MEM ^128^. Mitochondrial reads, PCR duplicates and low-quality alignments were removed using SAMtools ^129^ and Picard.

Peaks were called independently for each replicate using MACS2 v2.2.7.1 ^130^. Differential accessibility between control and *Chd1^iMN^* samples was assessed using DESeq2 ^127^. Differentially accessible regions (FDR <5%) were annotated to genomic features using HOMER v4.10 ^131^ and Ensembl annotations for GRCm38/mm10.

For fragment-length distributions, unnormalized alignment files were merged within each condition and fragment counts normalized to one million mapped fragments.

Genome-wide ATAC-seq coverage tracks were generated from merged alignment files using bamCoverage in deepTools v3.4.3 ^132^, with counts per million used for normalization. Coverage was calculated across 2-kb genomic bins and used for genome-wide comparisons between conditions.

Promoter annotations were obtained from Ensembl release 95 for GRCm38/mm10. Promoter ATAC-seq measurements were linked to RNA-seq expression data by gene ID. Coordinates of meiotic DSB hotspots and their corresponding SPO11-oligo counts were obtained from ^36^. Hotspots were ranked by SPO11-oligo coverage and the upper 10% were used for hotspot-centered analyses.

### Protein extraction and immunoblotting

Frozen tissues were homogenized in RIPA buffer containing 10 mM Tris-HCl, 140 mM NaCl, 1 mM EDTA, 1% Triton X-100, 0.1% SDS, 0.1% sodium deoxycholate, 1 mM Na3VO4 and 100 μM PMSF, supplemented with cOmplete Protease Inhibitor. Fibrous tissues, including spleen and thymus, were minced before homogenization.

Lysates were cleared by centrifugation at 21,000 × g for 15 min at 4 °C, passed twice through a 28-G needle and centrifuged again for 30 min. Protein concentrations were determined using Bio-Rad Protein Assay Dye Reagent against BSA standards.

Proteins were denatured in NuPAGE LDS sample buffer containing DTT for 5 min at 95 °C and separated by SDS–PAGE using either 3–8% Tris-acetate or 4–12% Bis-Tris gels. Proteins were transferred to 0.45-μm PVDF membranes using semi-dry or wet transfer.

Membranes were blocked for 1 h at room temperature in 4% milk in PBS containing 0.02% Tween-20 and incubated with primary antibodies for 16 h at 4 °C. Following washing, HRP-conjugated secondary antibodies were applied for 45 min at room temperature. Signals were detected using Clarity Western ECL or SuperSignal West Femto and imaged using a Bio-Rad ChemiDoc MP system.

### CHD1 immunoprecipitation

CHD1 immunoprecipitation and mass spectrometry were performed with modifications to a previously described procedure ^133^. Fresh whole testes or freshly isolated STA-PUT F2 cells were homogenized in EBN buffer containing 15 mM MgCl_2_, 20 mM EGTA, 80 mM β-glycerophosphate, 150 mM NaCl, 0.5% NP-40, 1 mM Na_3_VO_4_ and 100 μM PMSF supplemented with protease inhibitors. Lysates were clarified by sequential centrifugation and passage through a 28-G needle. Protein concentrations were determined using a Pierce BCA assay. For whole-testis immunoprecipitations, 4 mg total protein was used per sample; for F2 STA-PUT samples, 0.5 mg was used. Lysates were adjusted to 1 ml with EBN buffer and incubated with 1 μg anti-CHD1 or anti-HA control antibody for 1.5 h at 4 °C. Pre-blocked Protein A beads were then added and samples incubated for a further 1.5 h. Beads were washed once in EBN buffer and three times in EB buffer. A fraction of each sample was retained for immunoblot validation. Remaining beads were transferred to DNA LoBind tubes and processed for mass spectrometry. Protein A beads were pre-blocked for 1 h at 4 °C in 0.5 mg/mL BSA in EBN and washed twice before use.

### Preparation of CHD1 immunoprecipitation samples for mass spectrometry

Proteins bound to Protein A beads were eluted by three sequential incubations with 100 mM glycine, pH 2, and pooled eluates were neutralized using 1 M Tris-HCl, pH 8. Disulfide bonds were reduced using DTT for 30 min at room temperature and alkylated using iodoacetamide for 30 min in the dark. Excess iodoacetamide was quenched with DTT.

Proteins were digested overnight at 37 °C using sequencing-grade trypsin in 50 mM ammonium bicarbonate. Digestion was stopped by acidification with trifluoroacetic acid, and peptides were desalted using C18 StageTips ^134^. Reduction, alkylation and digestion conditions followed established proteomics workflows ^135^.

### LC-MS/MS analysis of CHD1 immunoprecipitations

Peptides were analyzed using an UltiMate 3000 RSLCnano system coupled to an Orbitrap Exploris 480 mass spectrometer equipped with a FAIMS Pro interface. Peptides were loaded onto a C18 trap column and separated using a 50-cm analytical column with a linear acetonitrile gradient in 0.1% formic acid at 230 nl/min. The analytical column was maintained at 30 °C.

The mass spectrometer was operated in data-dependent acquisition mode, with FAIMS compensation voltages alternating between −45 and −60 V. Survey scans were acquired over m/z 350–1,200 at 60,000 resolution. Selected precursor ions were fragmented by HCD and analyzed at 15,000 resolution with dynamic exclusion enabled. Acquisition followed established high-resolution proteomics approaches ^136^, with FAIMS used to improve proteome coverage and selectivity ^137^.

### Analysis of CHD1 immunoprecipitation mass spectrometry data

Raw files were separated by FAIMS compensation voltage and converted to mzXML format using FAIMS-MzXML-Generator. Data were analyzed using MaxQuant version 1.6.17.0 ^138,139^, against the UniProt mouse reference proteome (release 2020_01) supplemented with common contaminants.

Searches used full trypsin specificity with up to two missed cleavages. Carbamidomethylation of cysteine was specified as a fixed modification, with methionine oxidation and protein N-terminal acetylation as variable modifications. False-discovery rate was controlled at 1% at the peptide-spectrum-match and protein levels.

Label-free quantification was performed using MaxLFQ ^140^ with match-between-runs enabled. Output was further processed using Cassiopeia. Reverse hits, contaminants, proteins identified only by modified peptides, proteins supported by fewer than two razor peptides and proteins with insufficient quantitative values were excluded.

LFQ intensities were renormalized to the median abundance of a background-protein subset identified in control samples. Missing values were imputed using a downshifted and narrowed normal distribution as implemented in Perseus ^141^. Differential enrichment was assessed using LIMMA with Benjamini–Hochberg correction at a 5% false-discovery rate.

### Mass spectrometry analysis of acid-extracted histones

Acid-extracted histone samples were analyzed using a Vanquish Neo UHPLC system coupled to an Orbitrap Exploris 480 mass spectrometer equipped with a Nanospray Flex ion source. Peptides were loaded onto a PepMap Acclaim C18 trap column and separated on a 500-mm × 75-μm PepMap Acclaim C18 analytical column at 230 nl/min.

The gradient started at 0.5% mobile phase B (80% acetonitrile, 0.1% formic acid) and increased linearly to 47.5% B over 60 min, followed by an increase to 95% B, a 6-min hold and re-equilibration at starting conditions. The analytical column was maintained at 30 °C.

The Orbitrap Exploris 480 was operated in DDA mode. Full scans were acquired over m/z 350–1,200 at a resolution of 60,000. MS/MS spectra were acquired using HCD at a collision energy of 30%, isolation width of 1.2 m/z and Orbitrap resolution of 30,000. Precursors with charge states 2–6 were selected, and previously fragmented precursor ions were excluded for 10 s.

### Analysis of acid-extracted histone mass spectrometry data

RAW files were analyzed using Proteome Discoverer version 3.2.0.450 and MSAmanda version 3.0 ^142^. The peptide and fragment mass tolerance was ±10 ppm, with a maximum of two missed cleavages and tryptic specificity without proline restriction. Initial searches were performed against the UniProt mouse reference database supplemented with common contaminants and tagged proteins of interest.

Protein-level results were filtered to 1% FDR using Percolator ^143^. Proteins retained after the initial search were used to construct a reduced database for a second search. The second search additionally considered methionine oxidation; serine, threonine and tyrosine phosphorylation; lysine and protein N-terminal acetylation; arginine, lysine and protein N-terminal methylation; arginine and lysine dimethylation; lysine trimethylation; and N-terminal glutamine-to-pyroglutamate conversion as variable modifications. Modification-site localization was performed using ptmRS, based on phosphoRS ^144^. Results were filtered to 1% FDR at both PSM and protein level, with an additional minimum MSAmanda score of 150. Protein abundances were calculated using IMP-apQuant ^145^, by summing unique and razor peptides. Protein areas were normalized using iBAQ ^146^, followed by sum normalization between samples. Match-between-runs was applied where a peptide with a high-confidence peak area was identified by MS/MS in at least one run. Proteins were required to contain at least three quantified peptides for downstream analysis.

### Purification of recombinant CHD1

Full-length human CHD1 was expressed using a baculovirus system. The pFB-His6-MBP-3C-FLAG-HA-TEV-hCHD1 transfer vector was transformed into DH10EmBacY cells for bacmid generation, and recombinant colonies were identified by blue/white screening. Purified bacmid was verified by PCR and transfected into Sf9 cells using polyethylenimine to generate V0 and V1 viral stocks. For protein expression, 2 l Sf9 cells at 1.5 × 10^6 cells/mL were infected with V1 virus at a 1:200 ratio and incubated for 3 days at 27 °C. Cells were lysed by osmotic shock in 50 mM HEPES, pH 7.4, 0.2% Tween-20, 2 mM TCEP, 10 mM imidazole, 1 mM PMSF and EDTA-free protease inhibitors, followed by supplementation with 7% glycerol and 350 mM NaCl. Soluble extract was obtained by centrifugation at 150,000 × g for 30 min and incubated with Ni-NTA resin for 1 h. Resin was washed and protein eluted using 300 mM imidazole. Eluted protein was further purified by MBP affinity chromatography and Superdex 200 Increase 10/300 GL size-exclusion chromatography. Peak fractions were pooled, aliquoted, flash-frozen in liquid nitrogen and stored at −80 °C. For generation of untagged CHD1, pooled protein following MBP purification was incubated overnight at 4 °C with His-MBP-tagged TEV protease during sequential dialysis. The digest was passed over an MBPTrap HP column and CHD1-containing flow-through and wash fractions were pooled, concentrated, snap-frozen and stored at −80 °C.

### Purification of recombinant SYCE1

Human SYCE1 was expressed as an N-terminal His-MBP fusion containing a 3C cleavage site from a pOPINB-derived plasmid in E. coli BL21(DE3) RIL cells. Cultures were grown at 37 °C to OD600 = 0.6, induced with 0.5 mM IPTG and incubated overnight at 25 °C. Cells were lysed by sonication in 25 mM Tris, pH 8.0, 500 mM NaCl, 5% glycerol, 1 mM MgCl2 and 1 mM TCEP supplemented with EDTA-free protease inhibitor. Clarified lysates were applied to an MBPTrap HP column and eluted with 10 mM maltose. SYCE1-containing fractions were pooled, diluted to 200 mM NaCl, concentrated and further purified by size-exclusion chromatography using a HiLoad 16/600 Superdex 75 column. Peak fractions were pooled, concentrated, aliquoted, flash-frozen in liquid nitrogen and stored at −80 °C.

### SYCE1-CHD1 pull-down assay

MBP-SYCE1 and CHD1 were each used at 0.09 μM in 100 μl pull-down buffer containing 20 mM HEPES, pH 7.4, 50 mM NaCl, 5% glycerol and 1 mM TCEP. Proteins were incubated together for 30 min at 4 °C. Pre-equilibrated amylose resin was added and incubated for 1.5 h at 4 °C with gentle agitation. Resin was washed three times with pull-down buffer containing 0.02% NP-40, and bound proteins were eluted by boiling in reducing LDS sample buffer containing 12.5 mM DTT. Input, unbound and eluate fractions were analyzed by SDS–PAGE.

### Purification of recombinant histones and histone-octamer reconstitution

Recombinant human H2A, H2B, H3.1 and H4 were expressed individually in E. coli BL21(DE3)/RIL cells from pET21b(+) constructs and purified from inclusion bodies by cation-exchange chromatography as described previously ^147,148^. Cultures were grown at 37 °C to OD600 = 0.6 and induced with 0.2 mM IPTG for 3 h. Inclusion bodies were isolated, washed and solubilized overnight in 20 mM Tris-HCl, pH 7.5, 7 M guanidine hydrochloride and 10 mM DTT. Solubilized proteins were purified by cation-exchange chromatography using a HiTrap SP HP column, dialyzed against water and lyophilized. Histone octamers were assembled by mixing the four histones at equimolar ratios in unfolding buffer, followed by dialysis into 2 M NaCl refolding buffer and size-exclusion chromatography.

### Nucleosomal DNA preparation

DNA containing an end-positioned Widom 601 sequence was generated by PCR from an ST-601 template (EpiCypher) using a 5′ Cy5-labelled forward primer. PCR products were concentrated by ethanol precipitation, purified using 10% native PAGE, recovered by ethanol precipitation and dissolved in TE buffer.

### Nucleosome reconstitution

Nucleosomes were reconstituted by salt-gradient dialysis ^149^. Histone octamers and DNA were mixed at a 1:1 ratio in high-salt buffer containing 10 mM Tris-HCl, pH 7.5, 2 M NaCl, 1 mM EDTA, 0.05% IGEPAL and 1 mM DTT supplemented with 0.2 mg/mL BSA. Samples were dialyzed for 20 h against a linear gradient to low-salt buffer containing 50 mM NaCl.

### Nucleosome-remodelling assay

Restriction enzyme accessibility assays were performed using 20 nM nucleosomes and 40 nM CHD1 in buffer containing 10 mM Tris, pH 7.4, 40 mM NaCl, 4 mM MgCl2, 2 mM ATP and 1 mM DTT, together with 0.5 U/μL DpnII. Where indicated, 500 nM SYCE1 was added.

Reactions were incubated at 30 °C and stopped at the indicated time points by extraction with phenol:chloroform:isoamyl alcohol (25:24:1). DNA was resolved by 6% native PAGE and visualized using a Typhoon 5 phosphorimager. The fraction of cleaved DNA was quantified using GelAnalyzer v23.1.1. Experiments were performed independently at least three times.

### Image analysis

Image analysis was performed using ImageJ/Fiji, Huygens Professional, ZEN and custom macros or spreadsheet-based tools developed for this study. For metaphase-frequency analysis, tiled widefield images were imported into ImageJ and total nuclei identified using a custom macro. Metaphase-stage cells were identified by manual inspection and re-imaged at higher magnification to determine the number of Hoechst-positive chromosome bodies. For H3.4 measurements from STED images, SYBR Gold was used to define nuclear boundaries and custom ImageJ scripts were used to identify and quantify H3.4 signals within each nuclear region. For live-cell measurements, DIC images were used to define cell boundaries and Hoechst images to segment nuclei. For quantification of recombination-associated foci, SC-containing regions were defined and surrounding regions excluded. Foci were thresholded and identified as regions of interest using custom ImageJ macros. Focus number, area, morphology and intensity were extracted automatically. Where closely spaced foci merged following thresholding, foci were counted manually after excluding non-SC-associated signals. For paired whole-chromosome FISH measurements, FISH-signal boundaries and corresponding SYCP3 axes were manually traced in ImageJ. For nuclear and sex-body fluorescence measurements, regions of interest were defined for the whole nucleus and the γ-H2AX-positive sex body. Multiple intra- and extranuclear background measurements were used for background correction. For CHD1 intensity measurements normalized to SYCP3, whole-nuclear CHD1 and SYCP3 intensities were measured in ZEN 3.11 within Hoechst-defined nuclear regions. CHD1/SYCP3 ratios were calculated from exported data. Comparisons between genotypes were restricted to samples processed in parallel, stained on the same day and imaged using matched acquisition settings. Data generated in different laboratories were analyzed separately.

### Data inclusion and exclusion criteria

Because *Chd1* deletion in the inducible *Chd1^iMN^* model was not uniform across all nuclei, a subset of nuclei retained detectable CHD1 signal. To minimize confounding effects arising from incomplete depletion, pachytene-stage *Chd1^iMN^* nuclei containing more than 50 CHD1-positive foci were excluded from downstream analyses where CHD1 could be assessed. Numbers of excluded nuclei are reported in the corresponding figure legends. Where CHD1 could not be co-stained owing to antibody incompatibility, no CHD1-based exclusion criterion was applied. For all image-based analyses, only nuclei in which the structural features required for the corresponding measurement, including SC morphology or FISH-signal boundaries, could be clearly resolved were included.

### Statistical analysis

Unless otherwise stated, quantitative analyses were performed using data obtained from at least three independent biological replicates, with individual mice considered biological replicates. Multiple nuclei, chromosome spreads or cells measured within the same biological replicate were treated as technical observations unless otherwise specified. Exact sample sizes and the experimental unit used for each analysis are provided in the corresponding figure legends. Statistical analyses were performed using GraphPad Prism version 10.6.1. Pairwise comparisons were performed using two-sided Mann-Whitney U tests unless otherwise stated. Where multiple hypotheses were tested, P values were adjusted using the indicated multiple-comparisons procedure. Statistical tests specific to individual experiments are reported in the corresponding figure legends. Data are presented as indicated in each figure legend. Statistical significance was defined as P < 0.05.

## Supplementary Material

Supplement 1

## Figures and Tables

**Fig 1.**
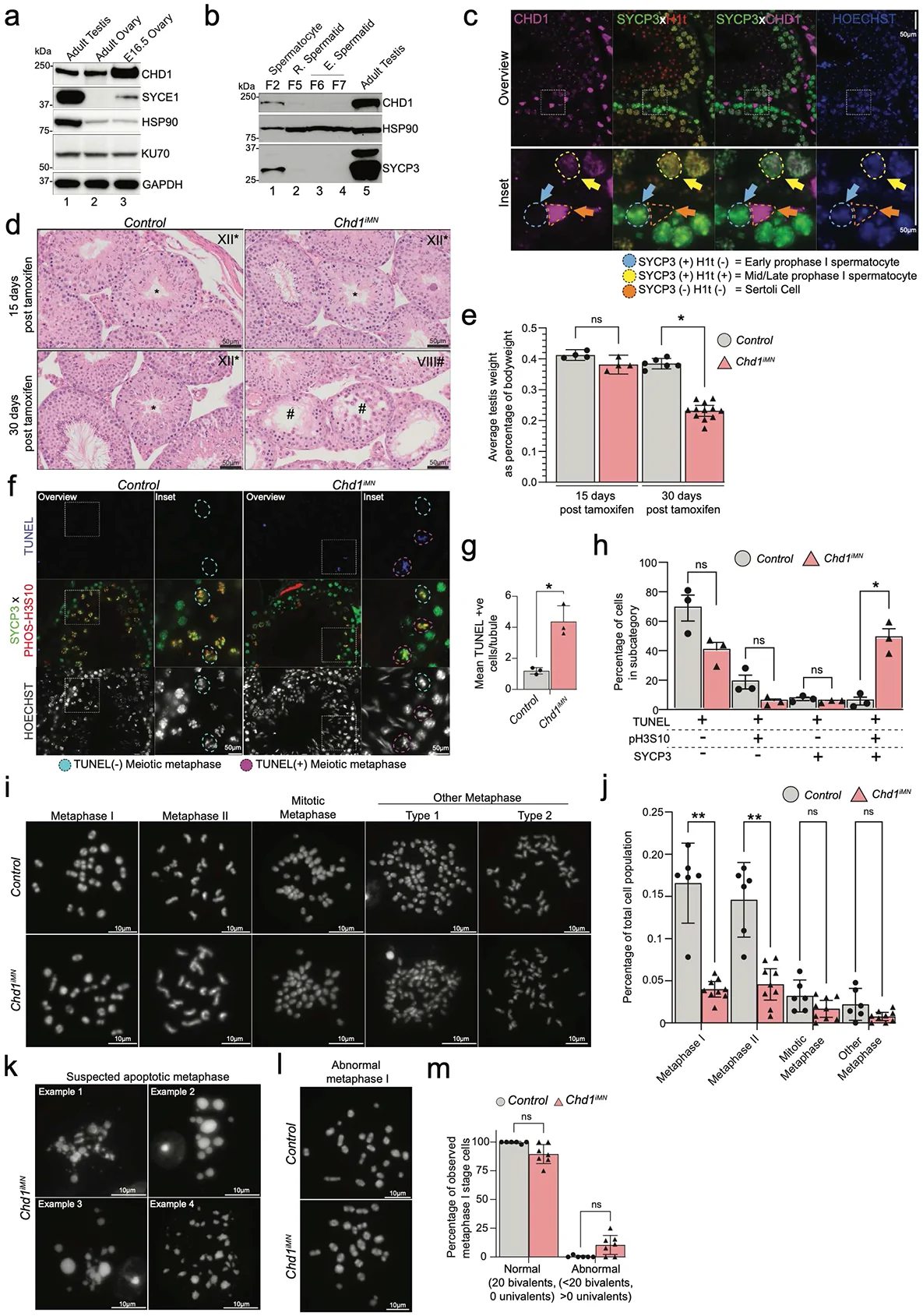
CHD1 exhibits temporally restricted expression in prophase I spermatocytes and is required for male gametogenesis **(a)** Immunoblot analysis of CHD1 in whole-tissue protein extracts prepared from testes of one 8-week-old adult male mouse, ovaries of one 8-week-old adult female mouse, or ovaries pooled from three embryonic day 16.5 female embryos. GAPDH and KU70 are shown as loading controls. HSP90 marks a protein highly expressed in whole-testis lysates, and SYCE1 indicates the presence of prophase I cells in the lysates. A total of 100 μg of protein was loaded per lane, and molecular weight marker positions are indicated. **(b)** Immunoblot analysis of CHD1 in protein extracts from testicular cell populations isolated by STA-PUT gravity sedimentation. Labels indicate the predominant cell type enriched in each of the fractions selected for analysis (F2, 5, 6 and 7). All fractions were obtained from the same starting sample, generated by pooling testes from four 8-week-old adult male mice before fractionation. The relative purity of each fraction is shown in Supplementary Fig. 1b. A whole-testis protein extract from one 8-week-old adult male mouse is included as an unfractionated reference sample. HSP90 is shown as a loading control, and SYCP3 indicates the presence of prophase I cells in the lysates. **(c)** Immunofluorescence staining of an adult wild-type testis section probed with anti-CHD1 antibody. CHD1 is shown in magenta. Enlarged inset panels show ringed cells staged according to nuclear morphology using Hoechst 33342 counterstaining, together with SYCP3, a marker of prophase I spermatocytes, and H1t, a marker of germ cells from mid-pachytene onwards. Images are representative of equivalent stainings performed in two biological replicates. Scale bars are as indicated. **(d)** Hematoxylin and eosin staining of adult control and *Chd1^iMN^* testis sections collected 15 or 30 days after tamoxifen injection. The seminiferous tubules marked with (*) are roughly staged as seminiferous stage XII as indicated in the top right-hand corner. (#) indicates seminiferous tubules observed in the *Chd1^iMN^* timepoint 30 days post tamoxifen treatment showing loss of germ cells at approximately seminiferous stage VIII. Images are representative of equivalent stainings performed in at least three biological replicates. Scale bars are as indicated. **(e)** Testis-to-body-weight ratios of adult control and *Chd1^iMN^* mice collected 15 or 30 days after tamoxifen injection. Data are shown as mean ± SD. Control, 15 days post tamoxifen, n = 4; *Chd1^iMN^*, 15 days post tamoxifen, n = 4; control, 30 days post tamoxifen, n = 6; *Chd1^iMN^*, 30 days post tamoxifen, n = 12 biological replicates. Kruskal–Wallis test with indicated comparisons: P = 0.5339, for 15 days post tamoxifen comparision; not significant, and P = 0.0045, for 30 days post tamoxifen comparision. **(f)** TUNEL staining of adult control and *Chd1^iMN^* testis sections collected 15 days after tamoxifen injection. TUNEL-positive cells are shown in blue. Enlarged inset panels show ringed cells staged according to nuclear morphology using Hoechst 33342 counterstaining, together with SYCP3, a marker of prophase I spermatocytes, and phospho-histone H3 Ser10, a marker of G2/M phase. Images are representative of equivalent stainings performed in three biological replicates. Scale bars are as indicated. **(g)** Quantification of the mean number of TUNEL-positive cells per seminiferous tubule in adult control and *Chd1^iMN^* testis sections stained as in (f). At least 30 seminiferous tubules were scored per mouse, and the mean number of TUNEL-positive cells per tubule was calculated for each mouse. Points represent individual mice, with three biological replicates per genotype. Data are shown as mean ± SD. Unpaired two-tailed Welch’s t test: P = 0.0289. **(h)** Quantification of the relative abundance of the indicated TUNEL-positive cell categories, expressed as a percentage of all TUNEL-positive cells identified in adult control and *Chd1^iMN^* testis sections stained as in (f). At least 30 seminiferous tubules were scored per mouse, and category proportions were calculated separately for each mouse. Points represent individual mice, with three biological replicates per genotype. Data are shown as mean ± SD. Multiple unpaired two-tailed Welch’s t tests with Holm correction: adjusted P = 0.1869, P = 0.1902, P = 0.2906, and P = 0.0416 for the indicated comparisons from left to right. **(i)** Hoechst 33342-stained metaphase spreads prepared from adult control and *Chd1^iMN^* testes. Labels indicate metaphase-stage classifications based on chromosome morphology. Two recurrent metaphase-like arrangements that could not be otherwise classified are labelled as “other metaphase”. Images are representative of equivalent preparations from at least six biological replicates. Scale bars are as indicated. **(j)** Quantification of the relative abundance of the metaphase-stage categories shown in (i), expressed as a percentage of the total number of cells identified in Hoechst 33342-stained metaphase spreads prepared from adult control and *Chd1^iMN^* testes. Measurements are from at least six biological replicates. For each biological replicate, percentages were calculated from at least 12,000 cells. Mann-Whitney U test: P < 0.001, P < 0.001, P < 0.001, P = 0.22 and P = 0.09 for the indicated comparisons from left to right. **(k)** Representative examples of condensed chromosomes with a characteristic apoptotic appearance identified in Hoechst 33342-stained metaphase spreads prepared from *Chd1^iMN^* testes. Scale bars are as indicated. **(l)** Representative examples of abnormal metaphase I-stage arrangements containing more than 20 Hoechst 33342-positive chromosome bodies, consistent with the presence of univalent chromosomes. Scale bars are as indicated. **(m)** Quantification of abnormal metaphase I cells, defined by the presence of more than 20 Hoechst 33342-stained chromosome bodies, expressed as a percentage of the total number of metaphase I cells identified in metaphase spreads prepared from adult control and *Chd1^iMN^* testes. Abnormal metaphase I-stage arrangements were classified as in (l). Measurements are from at least six biological replicates. At least four metaphase I cells were assessed per biological replicate, and at least 80 metaphase I cells were assessed per genotype overall. Mann–Whitney U test: P = 0.5981.

**Fig 2.**
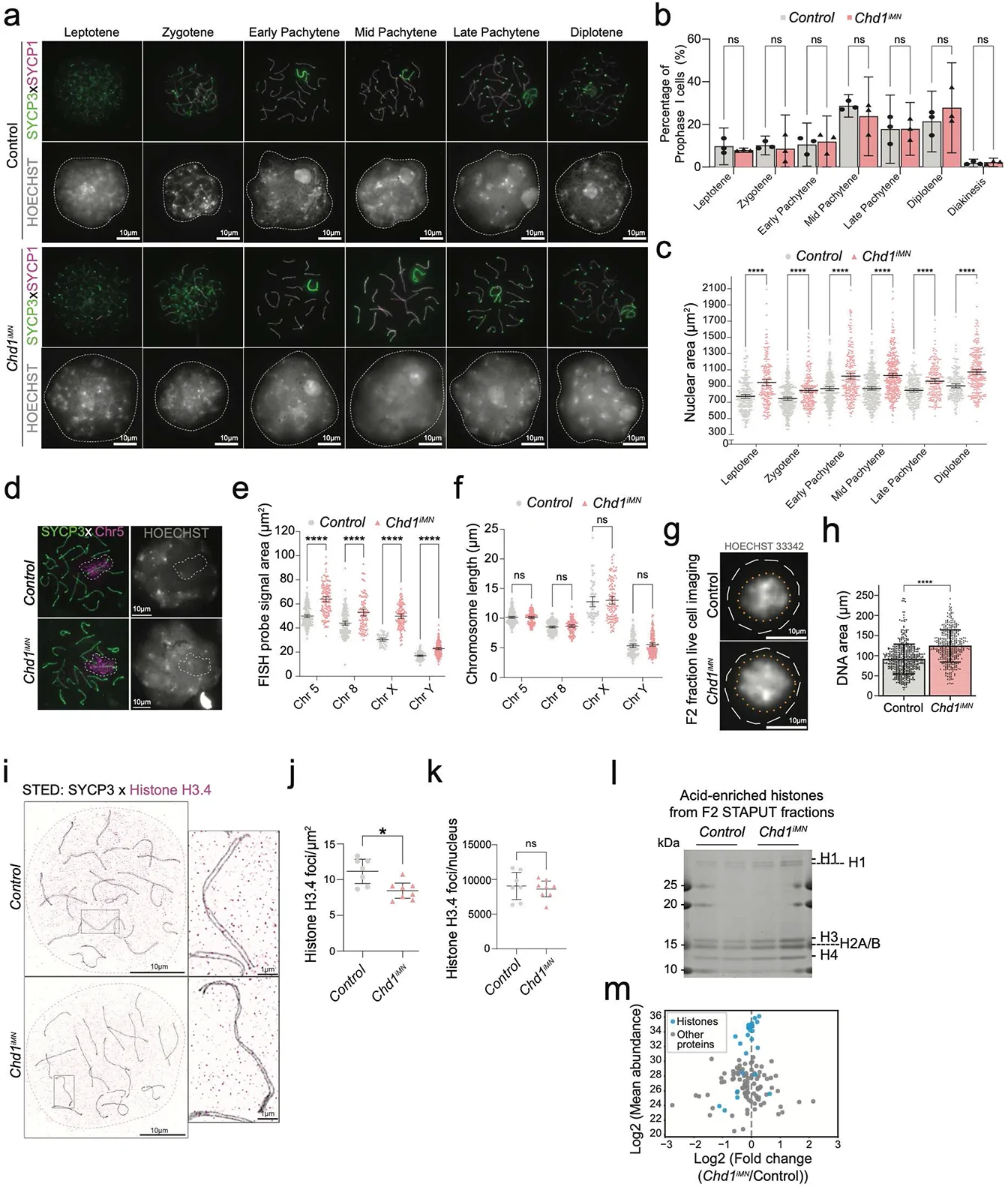
Chd1 loss causes chromatin decompaction while preserving SC length and chromatin composition **(a)** Nuclear surface spreads prepared from adult control and *Chd1*^*iMN*^ testes stained for SYCP3 and SYCP1 and counterstained with Hoechst 33342. SYCP3 is shown in green, SYCP1 in magenta and Hoechst 33342 in grey. Dotted lines in the Hoechst 33342 images indicate the perimeter of the nuclear signal. The area enclosed by this signal was quantified in (c). Images are representative of equivalent stainings performed in three biological replicates. Scale bars are as indicated. **(b)** Quantification of the relative abundance of prophase I substages in nuclear surface spreads prepared from adult control and *Chd1*^*iMN*^ testes, expressed as a percentage of all staged prophase I nuclei. Fields were selected without prior knowledge of prophase I substage, and all eligible nuclei within the selected fields were classified based on SYCP3 morphology and H1t staining. Percentages were calculated separately for each mouse. Measurements are from three biological replicates per genotype. Multiple two-sided Mann–Whitney U tests with Holm–Šídák correction: adjusted P = 0.997570, P = 0.997570, P = 0.972006, P = 0.972006, P > 0.999999, P = 0.997570, and P > 0.999999 for leptotene, zygotene, early pachytene, mid-pachytene, late pachytene, diplotene, and diakinesis, respectively. All comparisons were not significant. **(c)** Quantification of nuclear area across prophase I substages in nuclear surface spreads prepared from adult control and *Chd1*^*iMN*^ testes, based on the Hoechst 33342 signal outlined as in (a). Individual points represent nuclei pooled across at least eight biological replicates per genotype and are shown to illustrate the distribution of nucleus-level measurements. At least 190 nuclei were analyzed per substage and genotype overall, with at least 30 nuclei quantified per substage in each biological replicate. Horizontal lines indicate the mean ± SEM. Nucleus-level differences were assessed by two-way ANOVA using individual nuclei as observations, followed by multiple-comparisons testing; P<0.001 for all indicated genotype comparisons from left to right. This pooled analysis does not account for clustering of nuclei within biological replicates. Biological-replicate-level mean values were analyzed separately and are shown in Supplementary Fig 2h. **(d)** Pachytene-stage nuclear surface spreads prepared from adult control and *Chd1*^*iMN*^ testes stained for SYCP3 and hybridized with a whole-chromosome FISH probe against chromosome 5. SYCP3 is shown in green, the chromosome 5 FISH signal in magenta, and Hoechst 33342 in grey. Dotted lines indicate the perimeter of the FISH signal. The area enclosed by this signal was quantified in (e). Images are representative of equivalent stainings performed in at least two biological replicates. Scale bars are as indicated. **(e)** Quantification of whole-chromosome areas in pachytene-stage nuclei from adult control and *Chd1*^*iMN*^ testes, based on whole-chromosome FISH probes against chromosomes 5, 8, X and Y. For each FISH probe, measurements are from at least two biological replicates per genotype. For each biological replicate, at least 20 pachytene-stage nuclei were quantified. Total numbers of nuclei analyzed were: chromosome 5, control n = 175, *Chd1*^*iMN*^ n = 133; chromosome 8, control n = 159, *Chd1*^*iMN*^ n = 100; chromosome X, control n = 41, *Chd1*^*iMN*^ n = 116; chromosome Y, control n = 103, *Chd1*^*iMN*^ n = 170. Multiple Mann-Whitney U tests: P < 0.001 for all indicated comparisons from left to right. **(f)** Quantification of chromosome-axis length for individual bivalents positive for whole-chromosome FISH probes against chromosomes 5, 8, X and Y, based on SYCP3 signal length. Measurements were performed on the same nuclei analyzed in (e). Multiple Mann-Whitney U tests: P = 0.26, P = 0.50, P = 0.54 and P = 0.68 for the indicated comparisons from left to right; all comparisons were not significant. **(g)** Live-cell imaging of cells isolated by STA-PUT gravity sedimentation from testes pooled from four adult control or four adult *Chd1*^*iMN*^ mice. Representative images were acquired from culture plates seeded with one million cells. The imaged cells are enriched for spermatocytes; see Supplementary Figs. 2e-g for estimates of STA-PUT fraction purity. White dashed outlines indicate cell boundaries determined by DIC imaging. Hoechst 33342 was used as a live-cell dye to visualize nuclear area, indicated by orange dotted lines. Scale bars are as indicated. **(h)** Quantification of Hoechst 33342-positive nuclear area in spermatocyte-enriched F2 fraction cells isolated from adult control and *Chd1*^*iMN*^ testes, as in (g). Mann-Whitney U test: P < 0.001. Control, n = 499 cells; *Chd1*^*iMN*^, n = 391 cells. **(i)** STED imaging of nuclear surface spreads prepared from adult control and *Chd1*^*iMN*^ testes stained for SYCP3 and with an antibody raised against H3.4, which detects a broad spectrum of histone H3 variants. SYCP3 is shown in black and H3.4 in magenta. Dotted lines indicate the perimeter of the H3.4 signal. Images are representative of equivalent stainings performed in two biological replicates. Scale bars are as indicated. **(j)** Quantification of H3.4 focus density, calculated as the number of H3.4 foci per nucleus normalized to nuclear area. Measurements were obtained from one biological replicate per genotype, with nuclei treated as technical observations. Mann–Whitney U test: P = 0.0205. **(k)** Quantification of the number of H3.4 foci per nucleus in pachytene-stage nuclear surface spreads prepared from adult control and *Chd1*^*iMN*^ testes, as in (j). Measurements were obtained from at least seven nuclei from one biological replicate per genotype. Nuclei were treated as technical observations. Mann–Whitney U test: P = 0.8665. **(l)** Coomassie-stained gel showing acid-enriched histone protein preparations generated from spermatocyte-enriched F2 STA-PUT fractions. Lanes 1 and 2 show independent lysates, each prepared from testes pooled from four adult control mice. Lanes 3 and 4 show independent lysates, each prepared from testes pooled from four adult *Chd1*^*iMN*^ mice. The position of enriched core histone proteins is indicated, together with molecular weight marker positions. For each sample, 10% of the preparation was loaded per lane, corresponding to relative protein amounts isolated from approximately 100,000 cells. **(m)** Mass spectrometry analysis of acid-enriched histone protein preparations generated from spermatocyte-enriched F2 STA-PUT fractions, as in (l). Two independent control and two independent *Chd1*^*iMN*^ preparations were analyzed, each generated from testes pooled from four adult mice. For each sample, 80% of the preparation was used for analysis, corresponding to protein isolated from approximately 800,000 cells. Each point represents one detected protein. The x-axis shows the log2 fold change in mean protein abundance in *Chd1*^*iMN*^ relative to control, and the y-axis shows the log2 mean abundance across samples. Histone proteins are shown in blue and other detected proteins in grey. The dashed vertical line indicates no change between genotypes. Data are shown descriptively; no inferential statistical test was performed because only two independent preparations were available per genotype.

**Fig 3.**
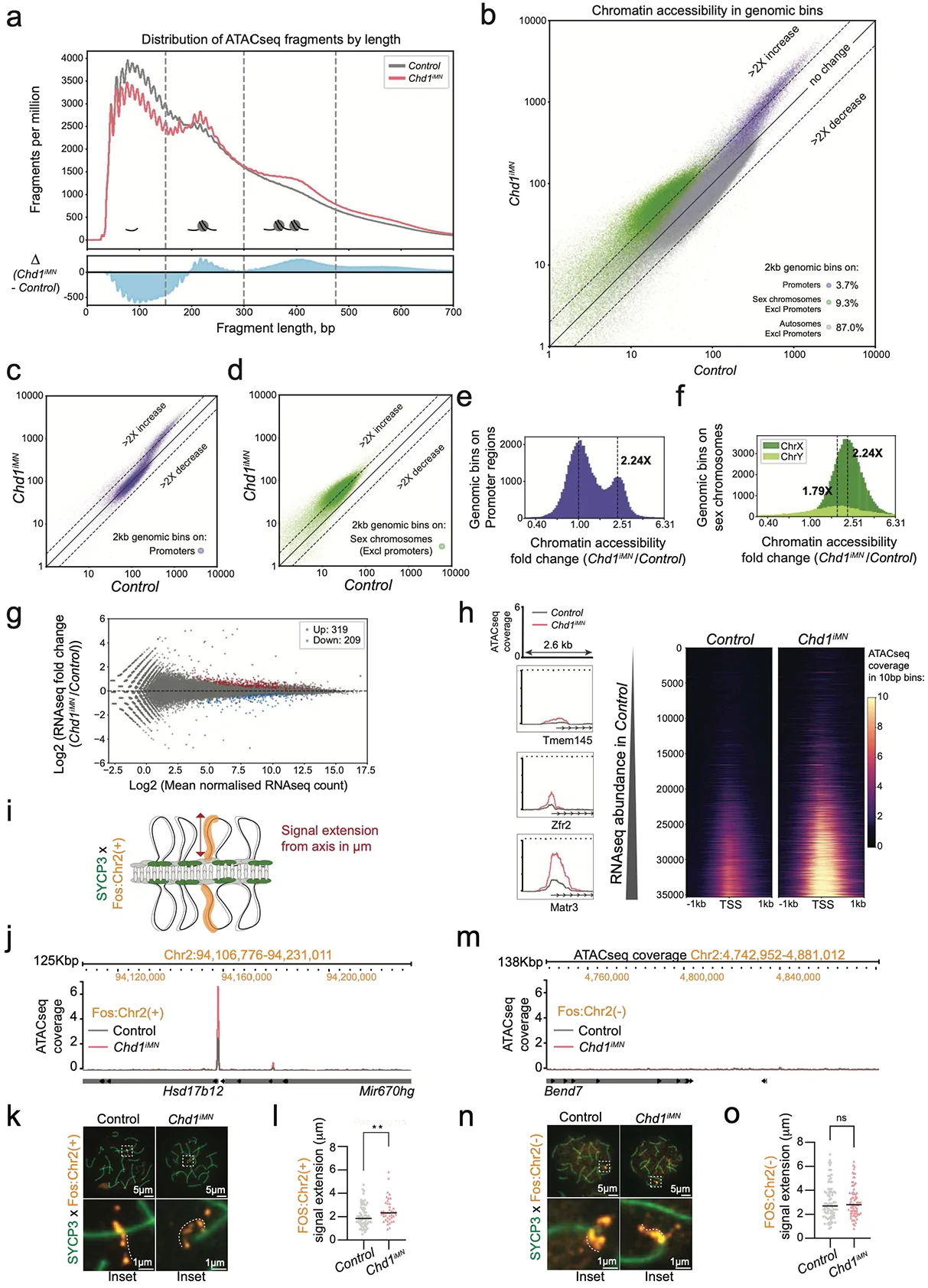
Chd1 loss alters chromatin accessibility primarily at highly transcribed promoters and is associated with local chromatin expansion **(a)** Distribution of ATAC-seq fragment lengths in control and *Chd1*^*iMN*^ spermatocytes. Sequencing reads from three biological replicates were aggregated within each genotype for visualization. Fragment counts were normalized to one million mapped fragments within each genotype. The lower blue histogram shows the difference in normalized fragment counts between *Chd1*^*iMN*^ and control. Fragments were classified as subnucleosomal (<150 bp), mononucleosomal (150–300 bp), or dinucleosomal (300–480 bp). **(b)** Scatterplot of aggregated ATAC-seq coverage across 2 kb genomic bins in control and *Chd1*^*iMN*^ spermatocytes. Sequencing reads from three biological replicates were aggregated within each genotype for visualization. Each point represents one genomic bin. The solid diagonal line indicates equal coverage between genotypes, and dashed lines indicate twofold differences. Promoter-overlapping bins are shown in purple, sex-chromosome bins in green, and remaining autosomal bins in grey. **(c)** Promoter-overlapping genomic bins from the analysis shown in (b). **(d)** Chromosome X and Y genomic bins from the analysis shown in (b). **(e)** Distribution of chromatin-accessibility changes at annotated promoters. ATAC-seq fold change was calculated as the ratio of aggregated ATAC-seq coverage in *Chd1*^*iMN*^ relative to control within 2 kb regions centered on annotated transcription start sites. The x-axis shows the log10-transformed fold change, with corresponding linear fold-change values indicated for clarity. The y-axis shows the number of promoters within each of 500 histogram bins. Dashed lines mark the principal distribution modes, corresponding approximately to unchanged accessibility and a 2.24-fold increase in accessibility in *Chd1*^*iMN*^. **(f)** Distribution of chromatin-accessibility changes across chromosomes X and Y. ATAC-seq fold change was calculated as the ratio of aggregated ATAC-seq coverage in *Chd1*^*iMN*^ relative to control across 2 kb genomic bins. The x-axis shows the log10-transformed fold change, with corresponding linear values indicated for clarity. The y-axis shows the number of genomic bins within each of 300 histogram bins, plotted separately for chromosomes X and Y. Dashed lines mark the principal distribution modes, corresponding approximately to 2.17-fold and 1.84-fold increases for chromosomes X and Y, respectively. **(g)** MA plot of differential gene-expression analysis comparing *Chd1*^*iMN*^ and control spermatocytes from three biological replicates per genotype, performed using DESeq2. Each point represents one gene. The x-axis shows the log2 mean normalized RNA-seq count across all samples, and the y-axis shows the log2 fold change in RNA abundance in *Chd1*^*iMN*^ relative to control. Genes with significantly increased or decreased RNA abundance in *Chd1*^*iMN*^ are shown in red and blue, respectively, based solely on a Benjamini–Hochberg-adjusted P value < 0.01. Non-significant genes are shown in grey. In total, 319 genes were significantly upregulated and 209 were significantly downregulated. **(h)** ATAC-seq signal at promoter regions stratified by RNA abundance in control spermatocytes. Genes were grouped and ordered according to RNA abundance in control samples, expressed as transcripts per million (TPM), and the same gene order was used for control and *Chd1*^*iMN*^ heatmaps. Genes with no detectable expression were excluded. A total of 35,317 genes were included and divided into four expression groups: low, 0.0001–0.01 TPM, n=1,663; mid-low, 0.01–1 TPM, n=16,947; mid-high, 1–100 TPM, n=15,010; and high, >100 TPM, n=1,697. Heatmaps show normalized ATAC-seq coverage across 2 kb regions centered on annotated transcription start sites, calculated in 10-bp bins. Sequencing reads from three biological replicates were merged within each genotype for visualization. Genes were oriented according to transcriptional direction such that upstream regions are shown to the left and downstream regions to the right. Tmem145, Zfr2, and Matr3 are shown as representative examples of low-, intermediate-, and high-expression genes, respectively. The 2.6-kb scale bars apply to the example browser tracks and are independent of the ±1-kb promoter window shown in the heatmaps. **(i)** Schematic illustration of the fosmid DNA-FISH approach used to measure chromatin-loop extension from the synaptonemal complex. The example shows three consecutive fosmids labelling one approximately 125Kb region on each homolog of chromosome 2. The genomic region spanned by these fosmids is shown in (j). Red arrows indicate measurements from the synaptonemal complex, labelled by SYCP3, to the distal edge of each fosmid signal. **(j)** ATAC-seq signal in control and *Chd1*^*iMN*^ spermatocytes across a chromosome 2 region spanned by fosmid probes. This region shows significantly increased ATAC-seq coverage in *Chd1*^*iMN*^ and is denoted by “+”. **(k)** Representative images of fosmid DNA-FISH staining for the chromosome 2 region shown in (j). Scale bars are as indicated. **(l)** Quantification of fosmid-signal extension from the SYCP3-labelled chromosome axis for the chromosome 2 regions shown in (j). For each nucleus, one extension measurement was obtained from the chromosome axis to the distal edge of the fosmid signal, as illustrated in (i), and expressed in μm. Two independent biological analyses were performed and both produced the same direction of effect. At least 43 nuclei were analyzed per genotype in both analyses. Mann–Whitney U tests were used to compare nucleus-level measurements between genotypes within each analysis. Mann–Whitney U test for depicted comparison: P = 0.0027. Control, n = 68 nuclei; *Chd1*^*iMN*^, n = 43 nuclei. **(m)** ATAC-seq signal in control and *Chd1*^*iMN*^ spermatocytes across a chromosome 2 region spanned by fosmid probes. This region showed no detectable change in ATAC-seq coverage between genotypes and is denoted by “-”. **(n)** Representative images of fosmid DNA-FISH staining for the chromosome 2 region shown in (m). SYCP3 marks the chromosome axis, and the fosmid-probe signal marks the labelled chromatin region. Scale bars are as indicated. **(o)** Quantification of fosmid-signal extension from the SYCP3-labelled chromosome axis for the chromosome 2 region shown in (m). For each nucleus, one extension measurement was obtained from the chromosome axis to the distal edge of the fosmid signal, as illustrated in (i), and expressed in μm. Two independent biological analyses were performed and both produced the same direction of effect. At least 70 nuclei were analyzed per genotype in both analyses. Mann–Whitney U tests were used to compare nucleus-level measurements between genotypes within each analysis. Mann–Whitney U test for depicted comparison: P = 0.6904, not significant.

**Fig 4.**
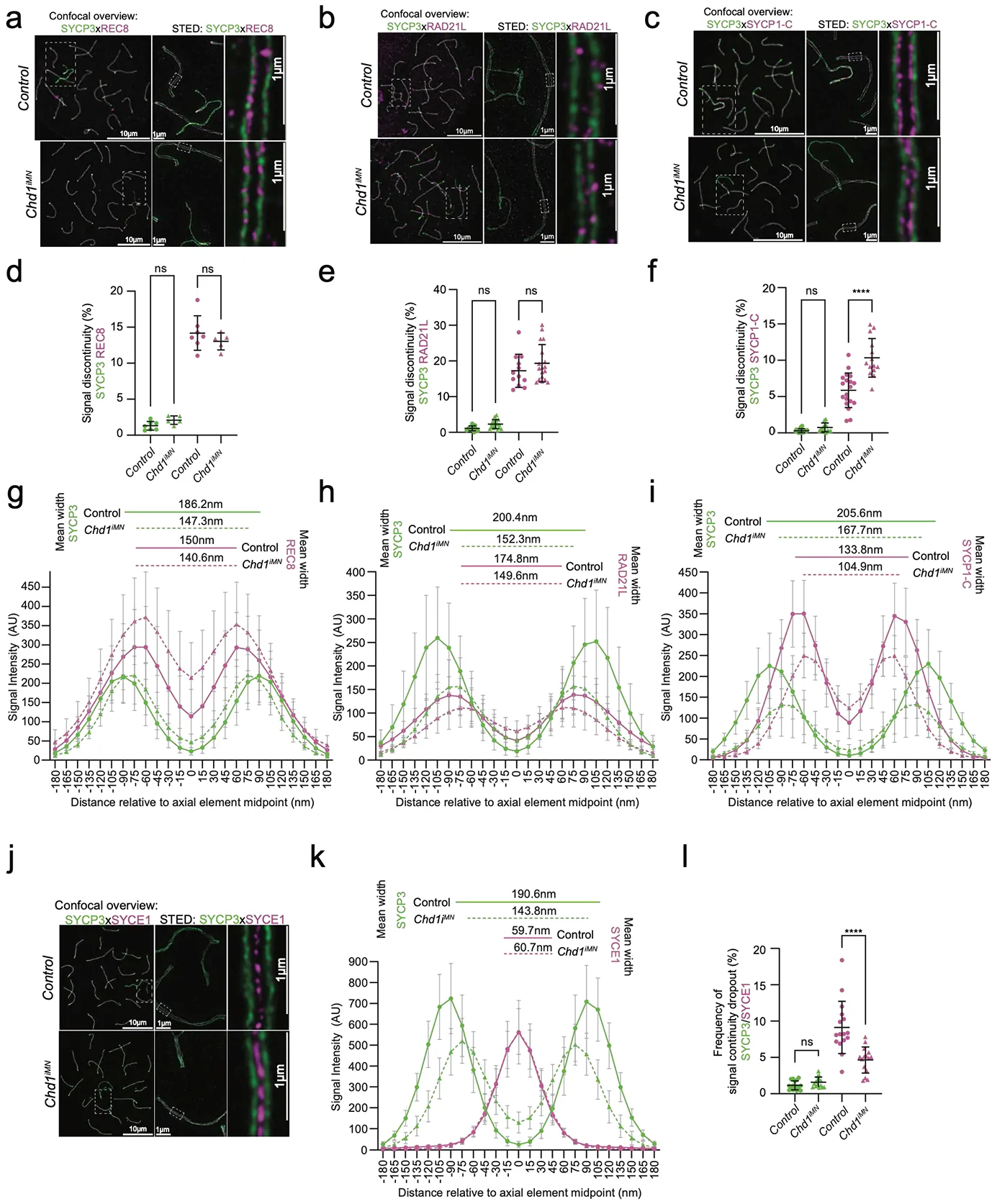
CHD1 loss narrows the synaptonemal complex and alters the nanoscale organization of cohesin and SC components **(a)** STED imaging of early pachytene-stage nuclear surface spreads prepared from one adult control and one *Chd1*^*iMN*^ biological replicate and stained for SYCP3 and REC8. SYCP3 is shown in green and REC8 in magenta. Multiple nuclei were examined for each genotype, and representative images are shown. Scale bars are as indicated. **(b)** As in (a), but stained for SYCP3 and RAD21L. SYCP3 is shown in green and RAD21L in magenta. Scale bars are as indicated. **(c)** STED imaging of pachytene nuclear surface spreads prepared from adult control and *Chd1*^*iMN*^ testes stained for SYCP3 and the C-terminus of SYCP1. SYCP3 is shown in green and SYCP1-C in magenta. Scale bars are as indicated. **(d)** Quantification of SYCP3 and REC8 signal discontinuity in early pachytene nuclei shown in (a). For each nucleus, multiple intensity profiles were averaged, and signal discontinuity was calculated as the percentage of measurements falling below 50% of the median signal intensity. SYCP3 and REC8 were measured in the same nuclei. Two-way repeated-measures ANOVA followed by multiple-comparisons testing: SYCP3, P = 0.9706; REC8, P = 0.3350. Control, n = 7 nuclei; *Chd1*^*iMN*^, n = 5 nuclei. Measurements were obtained from one biological replicate per genotype and therefore represent nucleus-level technical observations. **(e)** Quantification of SYCP3 and RAD21L signal discontinuity in early pachytene nuclei shown in (b), calculated as in (d). SYCP3 and RAD21L were measured in the same nuclei. Two-way repeated-measures ANOVA followed by multiple-comparisons testing: SYCP3, P = 0.9090; RAD21L, P = 0.4873. Control, n = 12 nuclei; *Chd1*^*iMN*^, n = 18 nuclei. Measurements were obtained from one biological replicate per genotype and therefore represent nucleus-level technical observations. **(f)** Quantification of SYCP3 and SYCP1-C signal discontinuity in pachytene nuclei shown in (c), calculated as in (d). SYCP3 and SYCP1-C were measured in the same nuclei. Two-way repeated-measures ANOVA followed by multiple-comparisons testing: SYCP3, P = 0.9199; SYCP1-C, P < 0.0001. Control, n = 31 nuclei; *Chd1*^*iMN*^, n = 31 nuclei. Measurements were obtained from one biological replicate per genotype and therefore represent nucleus-level technical observations. **(g)** Grand-average line-trace profiles of SYCP3 and REC8 signal intensity generated from images as in (a). Profiles were generated from aligned and averaged traces from at least 15 pachytene-stage nuclei from one biological replicate per genotype. For each nucleus, an averaged line trace was generated from at least 500 individual aligned traces bisecting parallel SYCP3 signals. Measurements were taken at 15-nm intervals along each trace. Traces were aligned such that the midpoint between the two SYCP3 axes was positioned at 0 nm. Individual traces were obtained only from regions in which the SYCP3 signals were parallel. Control data are shown as solid lines and *Chd1*^*iMN*^ data as dotted lines. See Methods for a detailed description of the analysis. **(h)** Grand-average line-trace profiles of SYCP3 and RAD21L signal intensity, generated as in (g) from images shown in (b). **(i)** Grand-average line-trace profiles of SYCP3 and SYCP1-C signal intensity, generated as in (g) from images shown in (c). **(j)** STED imaging of pachytene-stage nuclear surface spreads prepared from a separate adult control and *Chd1*^*iMN*^ biological replicate pair and stained for SYCP3 and SYCE1. SYCP3 is shown in green and SYCE1 in magenta. Multiple nuclei were examined for each genotype, and representative images are shown. Scale bars are as indicated. **(k)** Grand-average line-trace profiles of SYCP3 and SYCE1 signal intensity, generated as in (g) from images shown in (j). **(l)** Quantification of SYCP3 and SYCE1 signal discontinuity in pachytene nuclei shown in (j), calculated as in (d). SYCP3 and SYCE1 were measured in the same nuclei. Two-way repeated-measures ANOVA followed by multiple-comparisons testing: SYCP3, P = 0.9567; SYCE1, P < 0.0001. Control, n = 15 nuclei; *Chd1*^*iMN*^, n = 16 nuclei. Measurements were obtained from one biological replicate per genotype and therefore represent nucleus-level technical observations.

**Fig 5.**
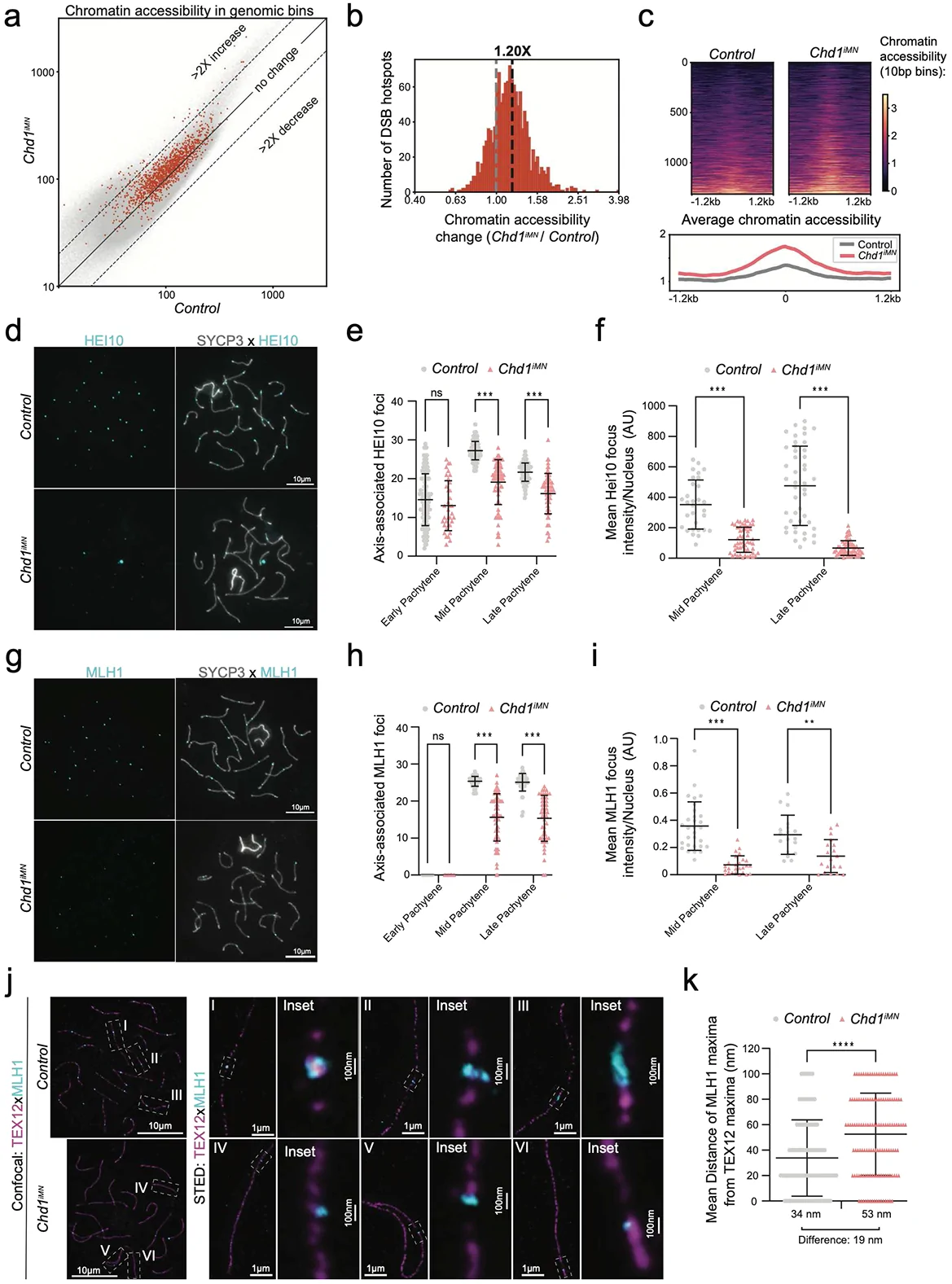
CHD1 regulates chromatin accessibility at recombination hotspots and enables HEI10 and MLH1 focus accumulation **(a)** Scatterplot of ATAC-seq coverage at the 10% hottest SPO11 DSB hotspots in control and *Chd1*^*iMN*^ spermatocytes. Three biological replicates were merged for each condition. Orange points represent 2 kb regions centered on the 10% most frequently used SPO11 DSB hotspots, n = 1,394. Grey points show the whole-genome cloud as in Fig. 3b, where the genome was divided into 2 kb bins to compare ATAC-seq coverage between control and *Chd1*^*iMN*^. Bins with unchanged chromatin accessibility between conditions lie along the diagonal, indicated by the solid black line. Bins with higher ATAC-seq coverage in *Chd1*^*iMN*^ are located above the diagonal, whereas bins with higher coverage in control are located below the diagonal. Dashed black lines indicate twofold increases or decreases in chromatin accessibility in *Chd1*^*iMN*^ relative to control. **(b)** Distribution of chromatin-accessibility changes at the 10% hottest SPO11 DSB hotspots. ATAC-seq fold change was calculated as the ratio of ATAC-seq coverage in *Chd1*^*iMN*^ relative to control within ±1 kb of annotated hotspot centers. Hotspots on sex chromosomes and hotspots overlapping promoter regions were excluded to avoid signal mixing. The x-axis shows fold change in log10 scale, with axis labels indicating the corresponding linear values. The y-axis indicates the number of hotspots in each of the 80 histogram bins, n = 1,314. The black dashed line marks the mean fold change, 1.20-fold, and the grey dashed line indicates no change. **(c)** ATAC-seq coverage at the 10% hottest SPO11 DSB hotspots, ranked by SPO11-oligo counts reported by Lange et al. Sex-chromosome hotspots and hotspots overlapping annotated promoters were excluded, leaving 1,314 hotspots. Heatmaps show CPM-normalized ATAC-seq coverage across ±1.2 kb of hotspot centres, generated from alignment files in which three biological replicates were merged within each genotype. The same hotspot order was used for control and *Chd1*^*iMN*^ heatmaps. Lower panel, mean CPM-normalized ATAC-seq coverage across all included hotspots, calculated in 10-bp bins over the same ±1.2 kb interval. **(d)** Pachytene-stage nuclear surface spreads prepared from adult control and *Chd1*^*iMN*^ testes stained for SYCP3 and HEI10. SYCP3 is shown in grey and HEI10 in cyan. Images are representative of equivalent stainings performed in three biological replicates. Scale bars are as indicated. **(e)** Quantification of the average number of HEI10 foci per nucleus across early, mid and late pachytene substages in control and *Chd1*^*iMN*^ samples, as in (d). A minimum of 30 nuclei were analyzed per genotype and substage across at least three independent biological replicates. Mann–Whitney U test: P = 0.21, not significant; P < 0.001; and P < 0.001 for the indicated comparisons from left to right. **(f)** Quantification of average HEI10 focus intensity per nucleus across early, mid and late pachytene substages in control and *Chd1*^*iMN*^ samples, as in (d). Measurements are from at least three biological replicates. A minimum of 30 nuclei were analyzed per genotype and substage across at least three independent biological replicates. For each nucleus, intensity measurements were taken for all foci passing a defined threshold; see Methods for details. Mann–Whitney U test: P < 0.001 for all indicated comparisons from left to right. **(g)** Pachytene-stage nuclear surface spreads prepared from adult control and *Chd1*^*iMN*^ testes stained for SYCP3 and MLH1, as in (d). SYCP3 is shown in grey and MLH1 in cyan. Images are representative of equivalent stainings performed in three biological replicates. Scale bars are as indicated. **(h)** Quantification of the average number of MLH1 foci per nucleus across early, mid and late pachytene substages in control and *Chd1*^*iMN*^ samples, as in (g). A minimum of 30 nuclei were analyzed per genotype and substage across at least three independent biological replicates. Mann-Whitney U test: P > 0.99, not significant; P < 0.001; and P < 0.001 for the indicated comparisons from left to right. For this analysis, based on additional staining for CHD1, 10 *Chd1*^*iMN*^ datapoints in total were found to exceed the cut off for CHD1 positivity (1 early pachytene nucleus, 5 mid pachytene nuclei and 4 late pachytene nuclei). See Methods for a more detailed explanation of exclusion criteria. **(i)** Quantification of average MLH1 focus intensity per nucleus across early, mid and late pachytene substages in control and *Chd1*^*iMN*^ samples, as in (g). A minimum of 30 nuclei were analyzed per genotype and substage across at least three independent biological replicates. For each nucleus, intensity measurements were taken for all foci passing a defined threshold; see Methods for details. Mann–Whitney U test: P < 0.001 for mid pachytene comparison and P = 0.0004 for late pachytene comparison. Excluded datapoints as in (h). **(j)** Confocal and STED imaging of nuclear surface spreads prepared from adult control and *Chd1*^*iMN*^ testes stained for TEX12 and MLH1. TEX12 is shown in magenta and MLH1 in cyan. Scale bars indicate the labelled distances. Images are representative of equivalent stainings performed in three biological replicates. Scale bars are as indicated. **(k)** Quantification of the distance between MLH1 focus maxima and TEX12 signal maxima in pachytene nuclei imaged as in (j). Distances were measured from 600-nm line traces drawn through each MLH1 focus and the corresponding TEX12 signal, as described in Methods. One distance measurement was obtained per MLH1 focus. Control, n = 197 MLH1 foci; *Chd1*^*iMN*^, n = 131 MLH1 foci, obtained from at least nine nuclei from one biological replicate per genotype. Mann–Whitney U test, P < 0.001.

**Fig 6.**
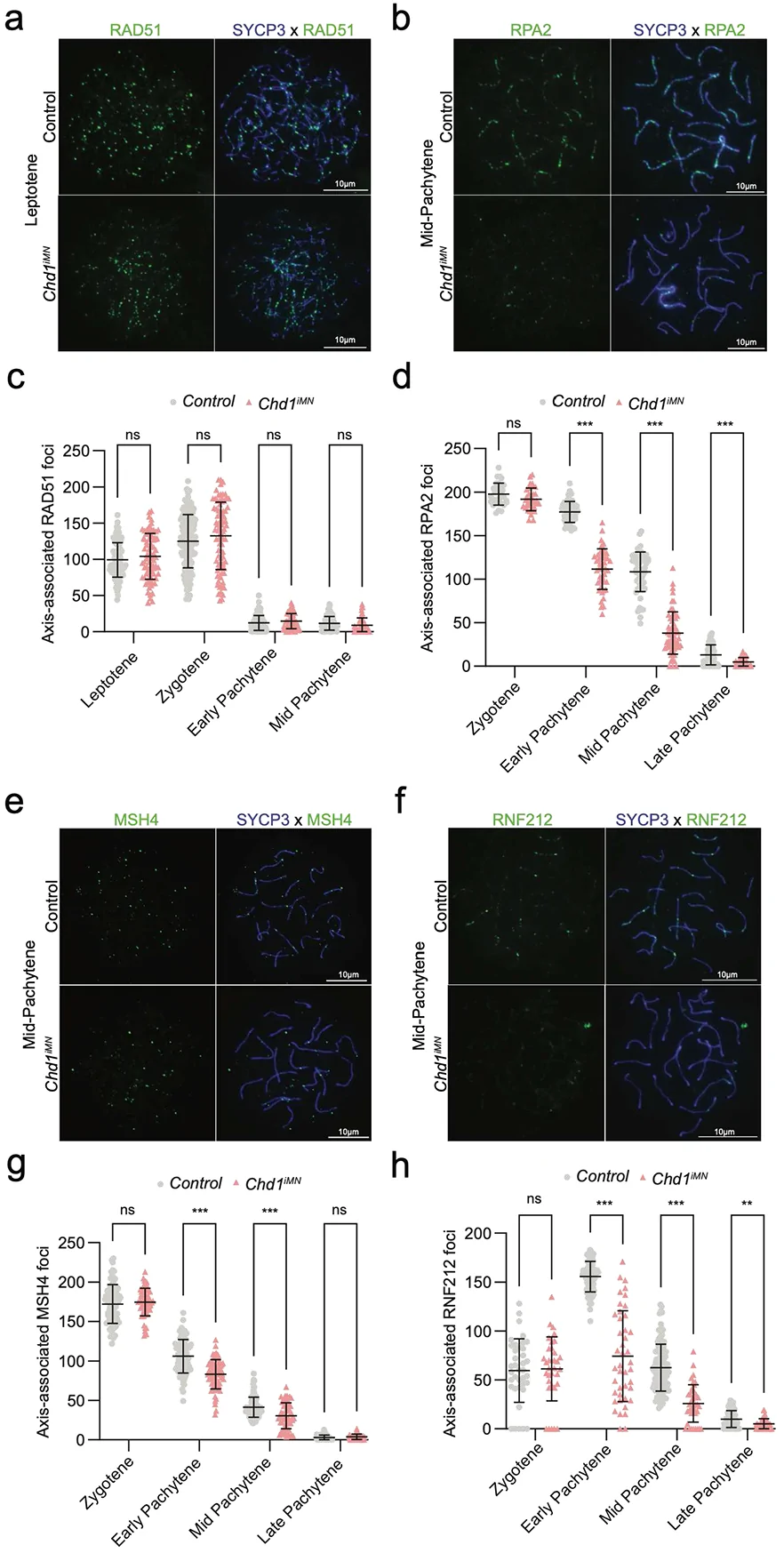
CHD1 is required for the maintenance of SC-associated crossover-promoting recombination factors **(a)** Representative nuclear surface spreads prepared from adult control and *Chd1*^*iMN*^ testes stained for SYCP3 and RAD51. SYCP3 is shown in blue and RAD51 in green. Images are stage-matched between genotypes, and the prophase I substage is indicated. Images are representative of equivalent stainings performed in three biological replicates. Scale bars are as indicated. **(b)** As in (a), but stained for SYCP3 and RPA2. SYCP3 is shown in blue and RPA2 in green. Scale bars are as indicated. **(c)** Quantification of the average number of SC-associated RAD51 foci per nucleus across prophase I substages in control and *Chd1*^*iMN*^ samples, as in (a). A minimum of 30 nuclei were analyzed per genotype and substage across at least three independent biological replicates. Mann-Whitney U test: P = 0.26, P = 0.14, P = 0.14 and P = 0.20 for the indicated comparisons from left to right; all comparisons were not significant. **(d)** Quantification of the average number of SC-associated RPA2 foci per nucleus across prophase I substages in control and *Chd1*^*iMN*^ samples, as in (b). A minimum of 30 nuclei were analyzed per genotype and substage across at least three independent biological replicates. Mann–Whitney U test: P = 0.07, not significant; P < 0.001; P < 0.001; P < 0.001; and P < 0.001 for the indicated comparisons from left to right. For this analysis, based on additional staining for CHD1, 9 *Chd1*^*iMN*^ datapoints in total were found to exceed the cut off for CHD1 positivity (1 early pachytene nucleus, 4 mid pachytene nuclei and 4 late pachytene nuclei). See Methods for a more detailed explanation of exclusion criteria. **(e)** As in (a), but stained for SYCP3 and MSH4. SYCP3 is shown in blue and MSH4 in green. Scale bars are as indicated. **(f)** As in (a), but stained for SYCP3 and RNF212. SYCP3 is shown in blue and RNF212 in green. Scale bars are as indicated. **(g)** Quantification of the average number of SC-associated MSH4 foci per nucleus across prophase I substages in control and *Chd1*^*iMN*^ samples, as in (e). A minimum of 30 nuclei were analyzed per genotype and substage across at least three independent biological replicates. Mann-Whitney U test: P = 0.4, not significant; P < 0.001; P < 0.001; and P = 0.11, not significant, for the indicated comparisons from left to right. **(h)** Quantification of the average number of SC-associated RNF212 foci per nucleus across prophase I substages in control and *Chd1*^*iMN*^ samples, as in (f). A minimum of 30 nuclei were analyzed per genotype and substage across at least three independent biological replicates. Mann-Whitney U test: P = 0.68, not significant; P < 0.001; P < 0.001; and P < 0.002 for the indicated comparisons from left to right. For this analysis, based on additional staining for CHD1, 12 *Chd1*^*iMN*^ datapoints in total were found to exceed the cut off for CHD1 positivity (4 early pachytene nuclei, 5 mid pachytene nuclei and 3 late pachytene nuclei). See Methods for a more detailed explanation of exclusion criteria.

**Fig 7.**
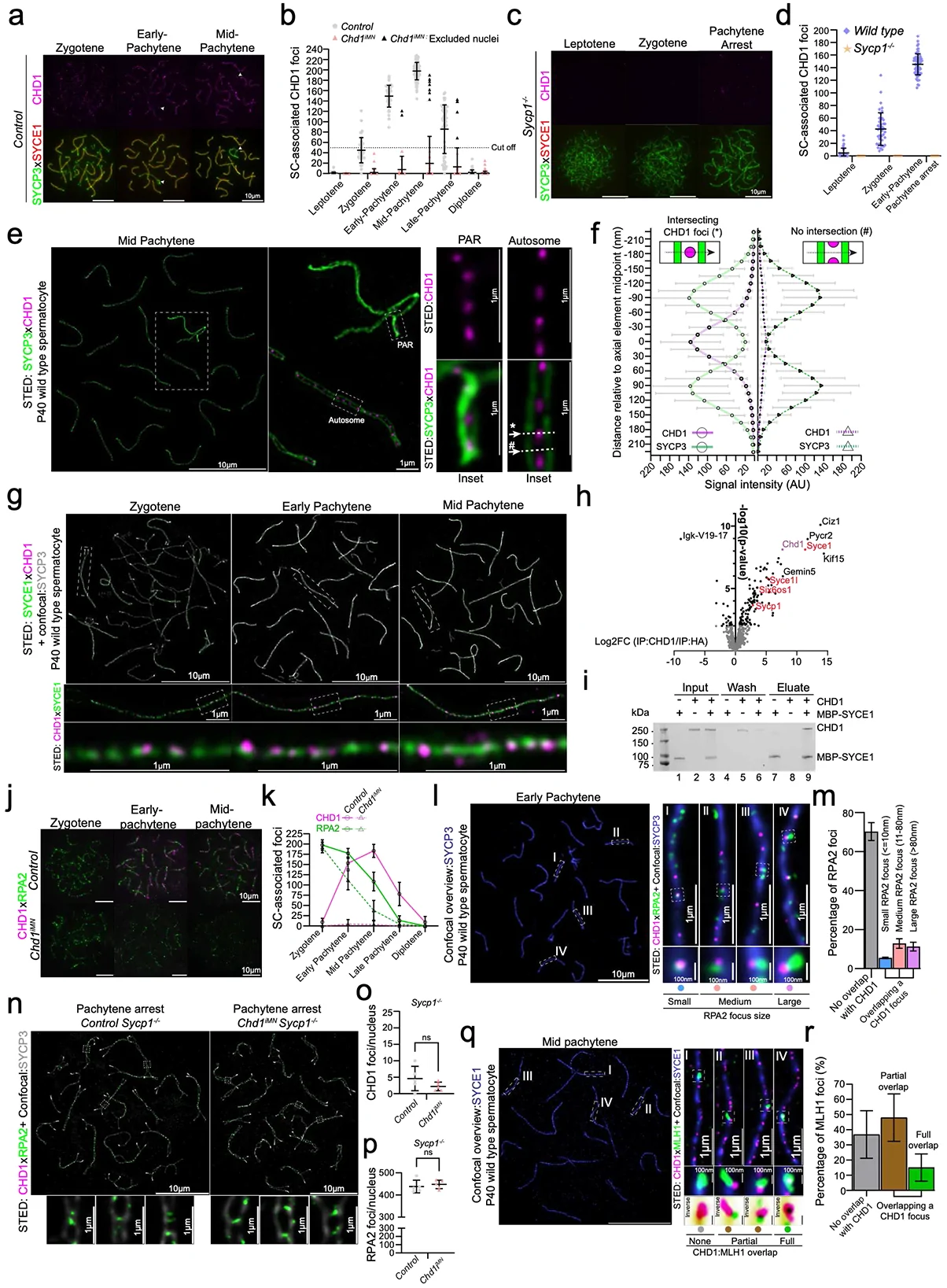
CHD1 localizes to the synaptonemal complex through interaction with the central element protein SYCE1 **(a)** Nuclear surface spreads prepared from adult control testes stained for CHD1, SYCP3 and SYCE1 across selected prophase I substages. CHD1 is shown in magenta, SYCP3 in green and SYCE1 in red. Images are representative of equivalent stainings performed in three biological replicates. Arrowheads indicate the pseudoautosomal region (PAR) of the sex chromosomes. Scale bars are as indicated. **(b)** Quantification of the number of SC-associated CHD1 foci per nucleus across prophase I substages in control and *Chd1*^*iMN*^ samples. Measurements were obtained from at least three independent biological replicates, with at least 30 nuclei quantified per substage. The dotted line indicates the predefined cutoff of >50 CHD1 foci. *Chd1*^*iMN*^ nuclei exceeding this cutoff are shown as black triangles and were classified as having incomplete CHD1 depletion. In total, 14 nuclei exceeded the cutoff: 2 early-pachytene nuclei, 7 mid-pachytene nuclei, and 5 late-pachytene nuclei. See Methods for the exclusion criteria. Data are shown descriptively; no statistical comparison was performed. (c) Nuclear surface spreads prepared from adult *Sycp1*^−/−^ testes stained for CHD1, SYCP3 and SYCE1 across selected prophase I substages, as in (a). CHD1 is shown in magenta, SYCP3 in green and SYCE1 in red. Images are representative of equivalent stainings performed in three biological replicates. Scale bars are as indicated. **(d)** Quantification of the average number of SC-associated CHD1 foci per nucleus across prophase I substages in adult wild-type and *Sycp1*^−/−^ samples, as in (c). Measurements are from at least two biological replicates. For each biological replicate, at least 30 nuclei were quantified per substage. **(e)** STED imaging of nuclear surface spreads prepared from adult wild-type testes stained for CHD1 and SYCP3. CHD1 is shown in magenta and SYCP3 in green. Insets highlight the pseudoautosomal region of the X and Y chromosomes and a representative autosome. Scale bars indicate the labelled distances. Images are representative of equivalent stainings performed in two biological replicates. Scale bars are as indicated. **(f)** Grand-average line-trace profiles of CHD1 and SYCP3 signal intensity. Profiles were generated from aligned and averaged traces from at least eight pachytene-stage nuclei from one adult wild-type biological replicate. For each nucleus, an averaged line trace was generated from at least 200 individual aligned traces bisecting parallel SYCP3 signals. Measurements were taken at 15-nm intervals along each trace. Traces were aligned such that the midpoint between the two SYCP3 axes was positioned at 0 nm. Individual traces were obtained only from regions in which the SYCP3 signals were parallel. Left, profiles from regions containing a CHD1 focus between the paired SYCP3 axes; right, profiles from regions lacking an intervening CHD1 focus. Data are shown descriptively. See Methods for a detailed description of the analysis. **(g)** Combined STED and confocal imaging of zygotene-, early-pachytene-, and mid-pachytene-stage nuclear surface spreads prepared from adult wild-type testes and stained for CHD1, SYCE1, and SYCP3. CHD1 is shown in magenta, SYCE1 in green, and SYCP3 in grey. CHD1 and SYCE1 were imaged by STED microscopy, whereas SYCP3 was imaged by confocal microscopy. Images are representative of equivalent stainings performed in two independent biological replicates. Scale bars are as indicated. **(h)** Volcano plot of proteins enriched in CHD1 immunoprecipitates relative to HA control immunoprecipitates from spermatocyte-enriched samples. Three independent biological replicates were analyzed per immunoprecipitation condition. Label-free protein intensities were quantified using MaxLFQ, normalized to a background protein subset identified in control samples, and missing values were imputed using a downshifted and narrowed normal distribution. Differential enrichment were analyzed using limma with Benjamini–Hochberg correction at a 5% false-discovery rate. Each point represents one detected protein. The x-axis shows the log2 fold change in protein abundance in CHD1 IP relative to HA IP, and the y-axis shows the −log10 Benjamini–Hochberg-adjusted P value. Proteins with less than twofold enrichment are shown in grey, whereas proteins with at least twofold enrichment are shown in black. CHD1 is shown in magenta, and selected synaptonemal complex proteins are shown in red and labelled. **(i)** In vitro pull-down assay using purified full-length human CHD1 and full-length MBP-tagged human SYCE1. CHD1 and MBP-SYCE1 were incubated together, captured using amylose resin, washed, and eluted under reducing conditions. Input, wash, and eluate fractions were analyzed by SDS-PAGE and Coomassie staining. CHD1 was recovered in the eluate when incubated with MBP-SYCE1, consistent with a direct interaction between the two proteins. Lane 1, MBP-SYCE1 input; lane 2, CHD1 input; lane 3, combined CHD1 and MBP-SYCE1 input; lanes 4–6, corresponding wash fractions; and lanes 7–9, corresponding eluate fractions. **(j)** Nuclear surface spreads prepared from adult control and *Chd1*^*iMN*^ testes stained for CHD1 and RPA2 across selected prophase I substages. CHD1 is shown in magenta and RPA2 in green. Images are representative of equivalent stainings performed in three biological replicates. Scale bars are as indicated. **(k)** Quantification of the average number of SC-associated CHD1 and RPA2 foci per nucleus across prophase I substages in control and *Chd1*^*iMN*^ samples, as in (j). Measurements are from at least three biological replicates. For each biological replicate, at least 30 nuclei were quantified per substage. Control data are shown as solid lines and *Chd1*^*iMN*^ data as dotted lines. For this analysis, based on additional staining for CHD1, 9 *Chd1*^*iMN*^ datapoints in total were found to exceed the cut off for CHD1 positivity (1 early pachytene nucleus, 4 mid pachytene nuclei and 4 late pachytene nuclei). See Methods for a more detailed explanation of exclusion criteria. **(l)** STED and confocal imaging of an early pachytene-stage nuclear surface spread prepared from adult wild-type testes stained for CHD1, RPA2 and SYCP3. CHD1 is shown in magenta, RPA2 in green and SYCP3 in blue. CHD1 and RPA2 were imaged by STED microscopy, and SYCP3 by confocal microscopy. Scale bars indicate the labelled distances. Insets highlight representative RPA2 foci of different sizes that overlap with CHD1. Images are representative of equivalent stainings performed in two biological replicates. Scale bars are as indicated. **(m)** Quantification of the percentage of RPA2 foci overlapping with a CHD1 focus in early pachytene-stage nuclei, measured by STED microscopy as in (l). Grey bars indicate RPA2 foci with no CHD1 overlap. Blue, red and purple bars indicate CHD1-overlapping RPA2 foci within the indicated size ranges. Representative examples are shown in (l). Co-localization was assessed for all RPA2 and CHD1 foci passing a defined threshold; see Methods for details. Measurements were performed on three nuclei from one biological replicate. In total, 608 RPA2 foci were analyzed. **(n)** STED and confocal imaging of pachytene-arrested nuclear surface spreads prepared from adult control *Sycp1*^−/−^ testes and adult *Chd1*^*iMN*^ *Sycp1*^−/−^ testes. Spreads were stained for CHD1, RPA2 and SYCP3. CHD1 is shown in magenta, RPA2 in green and SYCP3 in grey. CHD1 and RPA2 were imaged by STED microscopy, and SYCP3 by confocal microscopy. Scale bars indicate the labelled distances. Insets highlight persistent RPA2 bridges linking parallel SYCP3 axes in both genotypes. Images are representative of equivalent stainings performed in two biological replicates. Scale bars are as indicated. **(o)** Quantification of the number of SC-associated CHD1 foci per nucleus in pachytene-arrested control *Sycp1*^−/−^ and *Chd1*^*iMN*^ *Sycp1*^−/−^ samples, as in (n). Measurements were performed on at least five nuclei from one biological replicate per genotype. **(p)** Quantification of the number of SC-associated RPA2 foci per nucleus in pachytene-arrested control *Sycp1*^−/−^ and *Chd1*^*iMN*^ *Sycp1*^−/−^ samples, as in (n). Measurements were performed on at least five nuclei from one biological replicate per genotype. **(q)** STED and confocal imaging of a mid-to-late pachytene-stage nuclear surface spread prepared from adult wild-type testes stained for CHD1, MLH1 and SYCE1. CHD1 is shown in magenta, MLH1 in green and SYCE1 in blue. CHD1 and MLH1 were imaged by STED microscopy, and SYCE1 by confocal microscopy. Scale bars indicate the labelled distances. Insets highlight representative MLH1 foci with different degrees of overlap with CHD1. Images are representative of equivalent stainings performed in two biological replicates. Scale bars are as indicated. **(r)** Quantification of the percentage of MLH1 foci overlapping with a CHD1 focus in mid-to-late pachytene-stage nuclei, measured by STED microscopy as in (q). Grey bars indicate MLH1 foci with no CHD1 overlap, brown bars indicate partial overlap, and green bars indicate full overlap. Representative examples are shown in (q). Co-localization was assessed for all MLH1 and CHD1 foci passing a defined threshold; see Methods for details. Measurements were performed on eight nuclei from one biological replicate. In total, 172 MLH1 foci were analyzed.

**Fig 8.**
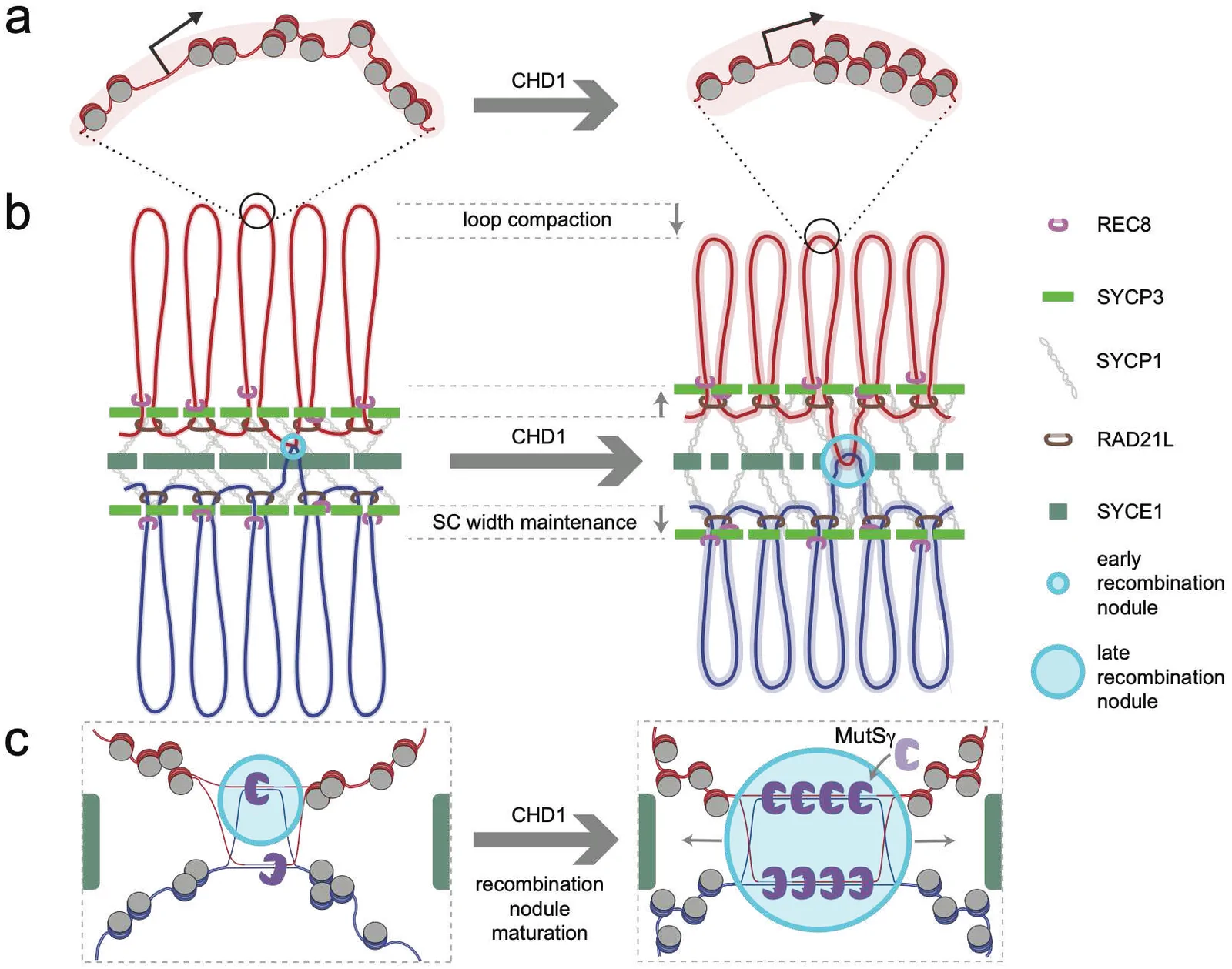
CHD1 coordinates meiotic chromatin organization to enable chromosome compaction, synapsis and crossing over. Working model: **(a)** At the nucleosome scale, CHD1 promotes local chromatin organization by modulating nucleosome positioning, in particular at actively transcribed regions. The pink shade is used to highlight the impact of nucleosome positioning on global chromatin compaction. **(b)** At the chromosome scale, CHD1 supports compact chromatin loops and maintains synaptonemal complex architecture, including appropriate spacing between axes (light green) and central-element components (dark green). The SC central region positioning of maturing recombination nodules (blue oval) is promoted by CHD1 function. **(c)** CHD1-mediated nucleosome remodeling creates a chromatin environment that supports stabilization of late-acting crossover factors and recombination nodule maturation, thereby promoting crossover formation. Note: The function of CHD1 depicted in (a) is likely to occur before synapsis and recombination nodule formation/maturation (b and c).
