# Supplementary material for: Chromatin remodeling coordinates chromosome compaction, synaptonemal complex architecture and meiotic crossing-over
Source: Res Sq. 2026 Sep 10:rs.3.rs-10849354. Preprint. [Version 1] doi: 10.21203/rs.3.rs-10849354/v1 (PMC13622809; doi:10.21203/rs.3.rs-10849354/v1)
Supplement: Supplementary file 1 [file NIHPPrs10849354v1-supplement-1.pdf]

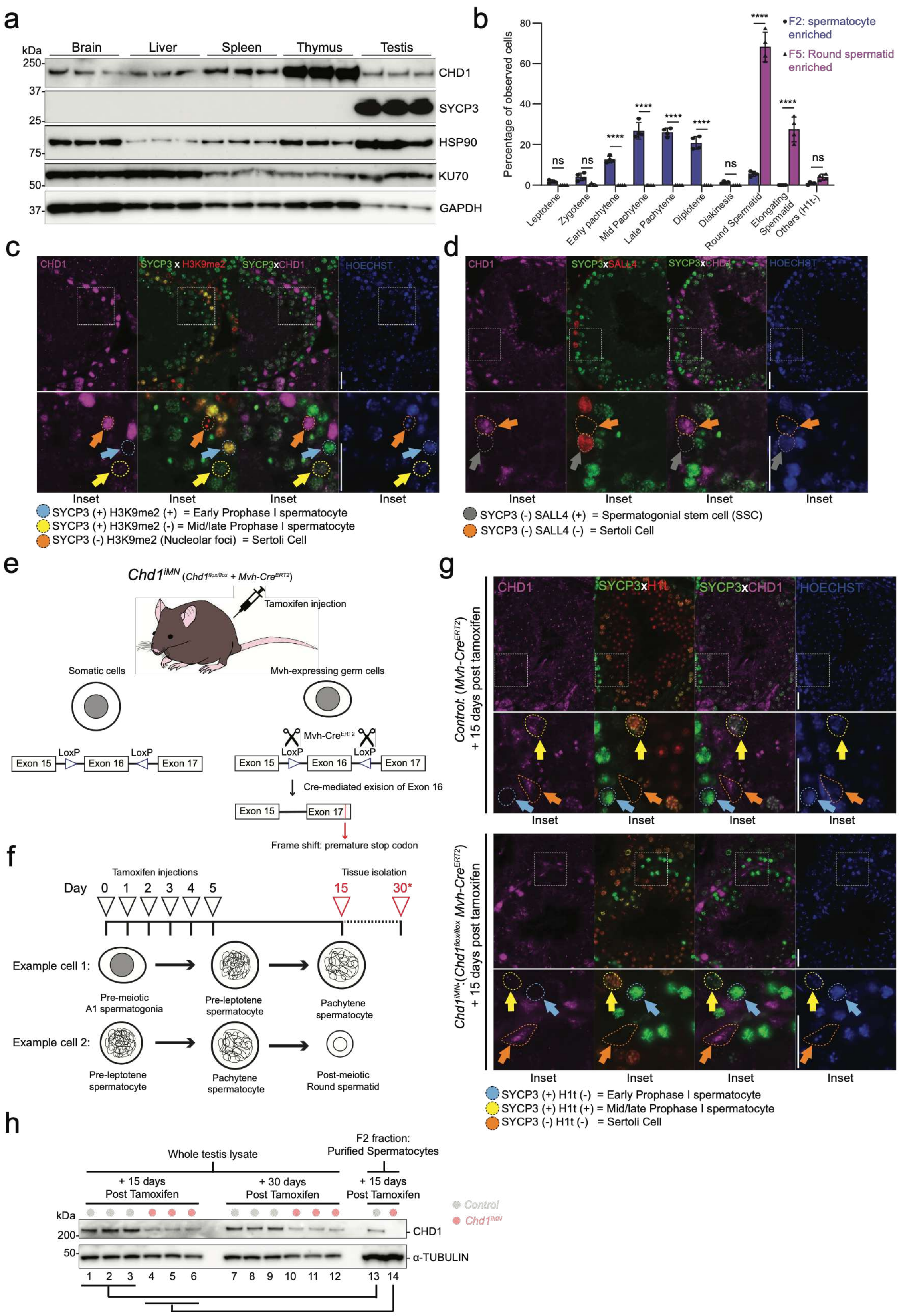
a
kDa
Brain
Liver
Spleen
Thymus
Testis
250-
37-
25-
75-
50-
37-
CHD1
SYCP3
HSP90
KU70
GAPDH
b
Percentage of observed cells
F2: spermatocyte enriched
F5: Round spermatid enriched
Leptotene
Zygotene
Early pachytene
Mid Pachytene
Late Pachytene
Diplotene
Diakinesis
Round Spermatid
Elongating Spermatid
Others (H1t-)
c
CHD1
SYCP3 x H3K9me2
SYCP3xCHD1
HOECHST
Inset
SYCP3 (+) H3K9me2 (+) = Early Prophase I spermatocyte
SYCP3 (+) H3K9me2 (-) = Mid/late Prophase I spermatocyte
SYCP3 (-) H3K9me2 (Nucleolar foci) = Sertoli Cell
d
CHD1
SYCP3xSALL4
SYCP3xCHD1
HOECHST
Inset
SYCP3 (-) SALL4 (+) = Spermatogonial stem cell (SSC)
SYCP3 (-) SALL4 (-) = Sertoli Cell
e
Chd1iMN (Chd1flox/flox + Mvh-CreERT2)
Tamoxifen injection
Somatic cells
Mvh-expressing germ cells
Exon 15
LoxP
Exon 16
Exon 17
Mvh-CreERT2
Cre-mediated exision of Exon 16
Frame shift: premature stop codon
f
Tamoxifen injections
Tissue isolation
Day 0 1 2 3 4 5 15 30*
Example cell 1:
Pre-meiotic A1 spermatogonia
Pre-leptotene spermatocyte
Pachytene spermatocyte
Example cell 2:
Pre-leptotene spermatocyte
Pachytene spermatocyte
Post-meiotic Round spermatid
g
Control: (Mvh-CreERT2) + 15 days post tamoxifen
Chd1iMN: (Chd1flox/flox Mvh-CreERT2) + 15 days post tamoxifen
CHD1
SYCP3xH1t
SYCP3xCHD1
HOECHST
Inset
SYCP3 (+) H1t (-) = Early Prophase I spermatocyte
SYCP3 (+) H1t (+) = Mid/late Prophase I spermatocyte
SYCP3 (-) H1t (-) = Sertoli Cell
h
Whole testis lysate
F2 fraction: Purified Spermatocytes
+ 15 days Post Tamoxifen
+ 30 days Post Tamoxifen
Control
Chd1iMN
kDa
200-
50-
CHD1
α-TUBULIN
1 2 3 4 5 6 7 8 9 10 11 12 13 14

Supplementary Fig. 1. CHD1 is expressed across somatic tissues and can be conditionally depleted in meiotic cells following tamoxifen treatment in *Chd1*^{iMN} mice

(a) Immunoblot analysis of CHD1 in whole-tissue protein extracts prepared from various tissues isolated from three separate adult wild-type male mice. GAPDH and KU70 are shown as loading controls. HSP90 marks a protein highly expressed in whole-testis lysates, and SYCP3 indicates the presence of prophase I cells in the lysates. A total of 100 µg of protein was loaded per lane for each of the three biological replicates, molecular weight marker positions are indicated. **(b)** Quantification of cell populations present in F2 and F5 fractions obtained by STA-PUT fractionation of cells isolated from wild-type testes. Values are shown as percentages of all cells identified in meiotic chromosome-spread preparations. Each matched F2–F5 pair represents one independent fractionation experiment, in which testes from four mice were pooled as input material. Four independent fractionation experiments were analyzed. At least 100 images were acquired per fraction and biological replicate. Statistical comparisons were performed using two-way repeated-measures ANOVA, with cell population and STA-PUT fraction as factors and fractionation experiment as the matched unit, followed by Šídák's multiple-comparisons test comparing F2 and F5 within each cell population. Adjusted P values from left to right were 0.1666, 0.2021, 0.0058, 0.0094, 0.0013, 0.0089, 0.1640, 0.0032, 0.0287, and 0.1389. **(c)** Immunofluorescence staining of an adult wild-type testis section for CHD1, SYCP3, and H3K9me2. CHD1 is shown in magenta, SYCP3 in green, H3K9me2 in red, and Hoechst 33342 in blue. Enlarged insets show selected cells identified by Hoechst 33342 nuclear morphology together with SYCP3, a marker of prophase I spermatocytes, and H3K9me2, which is enriched in early prophase I spermatocytes. Images are representative of equivalent stainings performed in two independent biological replicates. Scale bars are as indicated. **(d)** As in (c), but stained for CHD1, SYCP3 and SALL4, a marker of undifferentiated spermatogonial stem cells. CHD1 is shown in magenta, SYCP3 in green, SALL4 in red and Hoechst 33342 in blue. Images are representative of equivalent stainings performed in two biological replicates. Scale bars are as indicated. **(e)** Schematic representation of inducible *Chd1* deletion in Mvh-expressing germ cells in *Chd1*^{iMN} mice following tamoxifen treatment. **(f)** Schematic representation of the tamoxifen-dosing regimen used to induce Chd1 deletion. Black arrows indicate tamoxifen injections, and red arrows indicate experimental endpoints 15 and 30 days after the first injection. The expected developmental progression of two example germ cells is illustrated. In the first example, Chd1 deletion is initiated in a premeiotic spermatogonial cell that progresses to the pachytene stage over the 15-day interval. In the second example, deletion is initiated in a preleptotene spermatocyte that completes meiotic divisions and develops into a round spermatid. **(g)** Immunofluorescence staining of adult control and *Chd1*^{iMN} testis sections for CHD1, SYCP3, and H1t to assess CHD1 depletion. CHD1 is shown in magenta, SYCP3 in green, H1t in red, and Hoechst 33342 in blue. Enlarged insets show selected cells identified by Hoechst 33342 nuclear morphology together with SYCP3, a marker of prophase I spermatocytes, and H1t, a marker of germ cells from mid-pachytene onward. Yellow outlines indicate mid-to-late prophase I cells, which show reduced CHD1 signal in *Chd1*^{iMN} sections relative to control. Orange outlines indicate Sertoli cells, which retain strong CHD1 signal because they do not express Mvh and are therefore not targeted by the conditional deletion. Images are representative of equivalent stainings performed in two independent biological replicates. Scale bars are as indicated. **(h)** Immunoblot analysis of CHD1 in whole-testis protein extracts from control and *Chd1*^{iMN} mice collected 15 or 30 days after tamoxifen injection. Lanes 1–3, three control mice 15 days after tamoxifen injection; lanes 4–6, three *Chd1*^{iMN} mice 15 days after tamoxifen injection; lanes 7–9, three control mice 30 days after tamoxifen injection; lanes 10–12, three *Chd1*^{iMN} mice 30 days after tamoxifen injection. Lanes 13 and 14 show protein extracts from cells isolated by STA-PUT gravity sedimentation. In lane 13, fractionated cells originate from a subset of testis cells pooled from the three biological replicates shown in lanes 1–3. In lane 14, fractionated cells originate from a subset of testis cells pooled from the three biological replicates shown in lanes 4–6. Lanes 13 and 14 therefore represent CHD1 expression in spermatocytes present in the whole testes shown in lanes 1–3 and lanes 4–6, respectively. In *Chd1*^{iMN} whole-testis lysates shown in lanes 4–6 and lanes 10–12, residual CHD1 signal is expected to originate from non-Mvh-expressing cells that do not delete *Chd1*. α-tubulin is shown as a loading control. A total of 40 µg of protein was loaded per lane, and molecular weight marker positions are indicated.

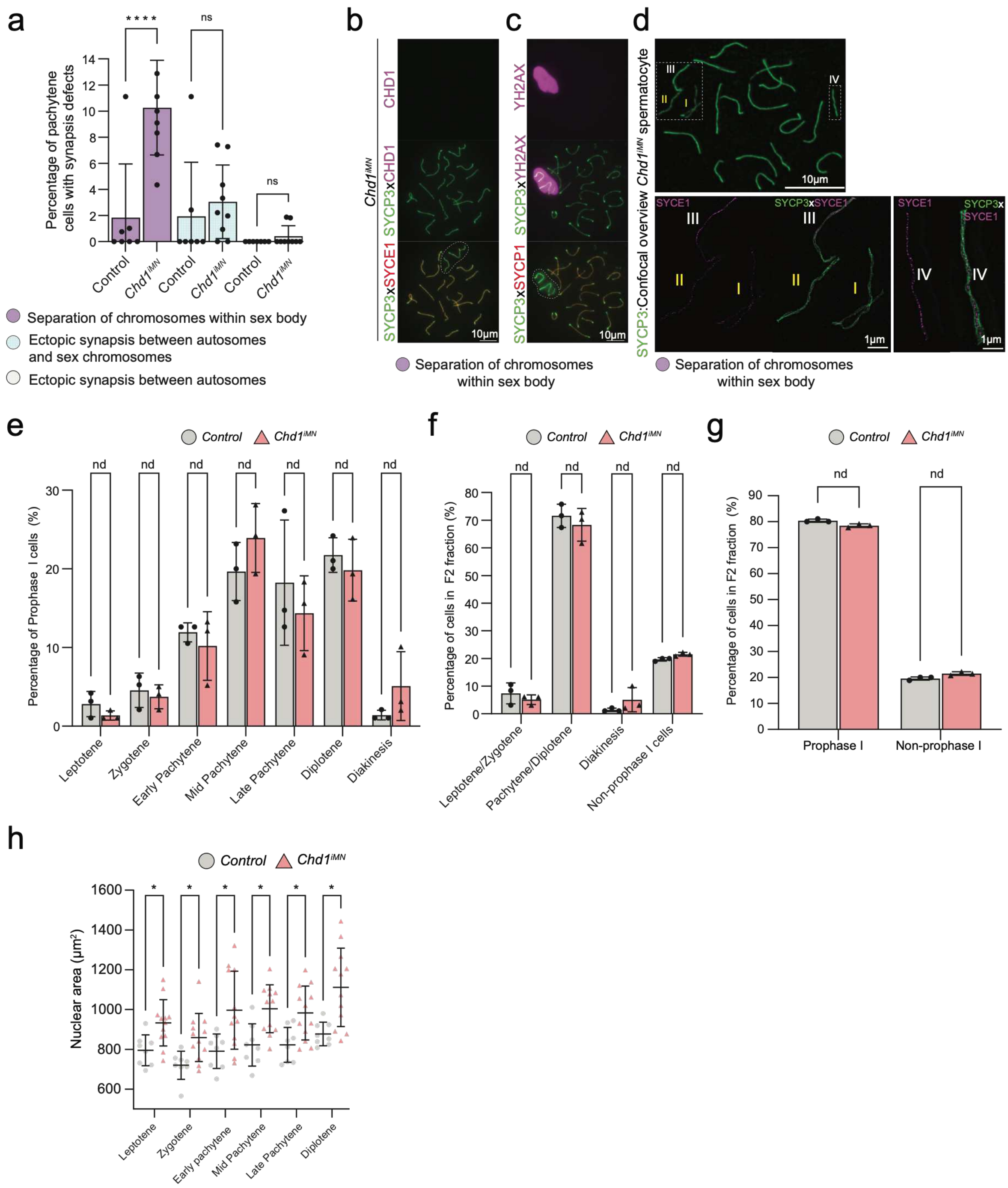

Supplementary Fig. 2. Synapsis defects primarily affecting sex chromosomes occur in a subset of pachytene-stage *Chd1^iMN^* spermatocytes

(a) Quantification of synaptic defects in pachytene-stage nuclear surface spreads prepared from adult control and *Chd1*^{iMN} testes. Bars show the relative frequencies of three defect classes: separation of chromosomes within the sex body, shown in pink; ectopic synapsis between sex chromosomes and autosomes, shown in cyan; and ectopic synapsis between autosomes, shown in grey. At least 40 pachytene nuclei were analyzed per biological replicate. Data are from 6 control and 9 *Chd1*^{iMN} biological replicates, and biological-replicate frequencies were used for statistical analysis. Ordinary two-way ANOVA: genotype × defect-class interaction, $F(2, 40) = 9.280$, $P = 0.0005$; genotype, $F(1, 40) = 23.25$, $P < 0.0001$; defect class, $F(2, 40) = 19.99$, $P < 0.0001$. Šídák's multiple-comparisons test comparing genotypes within each defect class: adjusted $P < 0.0001$, $P = 0.1820$, and $P = 0.9879$, respectively. **(b)** Representative nuclear surface spreads with separation of chromosomes within the sex body prepared from adult *Chd1*^{iMN} testes and stained for SYCP3, CHD1 and SYCE1. SYCP3 is shown in green, CHD1 in magenta and SYCE1 in red. Dotted outlines indicate the sex body, defined by distinct SYCP3 and SYCP1 staining together with Hoechst 33342 morphology. Scale bars are as indicated. **(c)** Representative nuclear surface spreads with separation of chromosomes within the sex body prepared from adult *Chd1*^{iMN} testes and stained for SYCP3, γ-H2AX and SYCP1. SYCP3 is shown in green, γ-H2AX in magenta and SYCP1 in red. Dotted outlines indicate the sex body, defined by γ-H2AX staining together with Hoechst 33342

morphology. Scale bars are as indicated. **(d)** STED and confocal imaging of a mid-to-late pachytene-stage nuclear surface spread with suspected separation of chromosomes prepared from adult *Chd1*^{iMN} testes and stained for SYCP3 and SYCE1. Confocal overview shows a nucleus with 21 SYCP3 positive segments indicating the presence of an additional chromosome segment. STED images are shown below. Chromosomes labelled I and II are interpreted as putative separated sex chromosomes based on their negativity for SYCE1. Chromosomes labelled III and IV are interpreted as correctly synapsed autosomes by their positivity for SYCE1 within the central region. Scale bars are as indicated. **(e)** Quantification of the relative abundance of prophase I cell types in spermatocyte-enriched F2 STA-PUT fractions from control and *Chd1*^{iMN} mice 15 days after tamoxifen treatment. Values are shown as percentages of all cells identified. Each data point represents one independent STA-PUT fractionation experiment, in which testes from four mice were pooled as input material. Three independent fractionation experiments were analyzed per genotype and treated as biological replicates. At least 100 images were acquired per biological replicate. Biological-replicate percentages were analyzed using multiple unpaired Welch's t tests with two-stage step-up Benjamini–Krieger–Yekutieli false-discovery-rate correction at Q = 1%. Adjusted q = 0.6271 for all seven indicated genotype comparisons; none passed the predefined FDR threshold. **(f)** Quantification of selected prophase I and non-prophase I cell populations in spermatocyte-enriched F2 STA-PUT fractions, as in (e). Biological-replicate percentages were analyzed using multiple unpaired Welch's t tests with two-stage step-up Benjamini–Krieger–Yekutieli false-discovery-rate correction at Q=1%. Adjusted q values were 0.4852, 0.4852, 0.4482, and 0.1046 for the indicated genotype comparisons from left to right; none passed the predefined FDR threshold. **(g)** Quantification of total prophase I and non-prophase I cell populations in spermatocyte-enriched F2 STA-PUT fractions, as in (e). Biological-replicate percentages were used for statistical analysis. Multiple unpaired Welch's t tests with two-stage step-up Benjamini–Krieger–Yekutieli false-discovery-rate correction at Q = 1%: q = 0.0261 for both comparisons; neither comparison passed the predefined FDR threshold. **(h)** Quantification of nuclear area across prophase I substages in nuclear surface spreads prepared from adult control and *Chd1*^{iMN} testes, based on the Hoechst 33342 signal outlined as in Fig. 2A. Multiple nuclei were quantified for each mouse and substage, and mouse-level mean nuclear areas were used for statistical analysis. Measurements are from 8 control and 12 *Chd1*^{iMN} biological replicates. Mean nuclear area was increased by 1.17- to 1.27-fold in *Chd1*^{iMN} spermatocytes across prophase I substages, corresponding to increases of 17.4%–26.8%. Two-way repeated-measures ANOVA with Geisser–Greenhouse correction: genotype, $F(1, 18) = 13.34$, $P = 0.0018$; prophase I substage, $F(3.425, 61.64) = 13.01$, $P < 0.0001$; genotype × substage interaction, $F(3.425, 61.64) = 1.077$, $P = 0.3705$. Šídák's multiple-comparisons test: adjusted P = 0.0299, 0.0264, 0.0329, 0.0154, 0.0288, and 0.0103 for the indicated genotype comparisons from left to right.

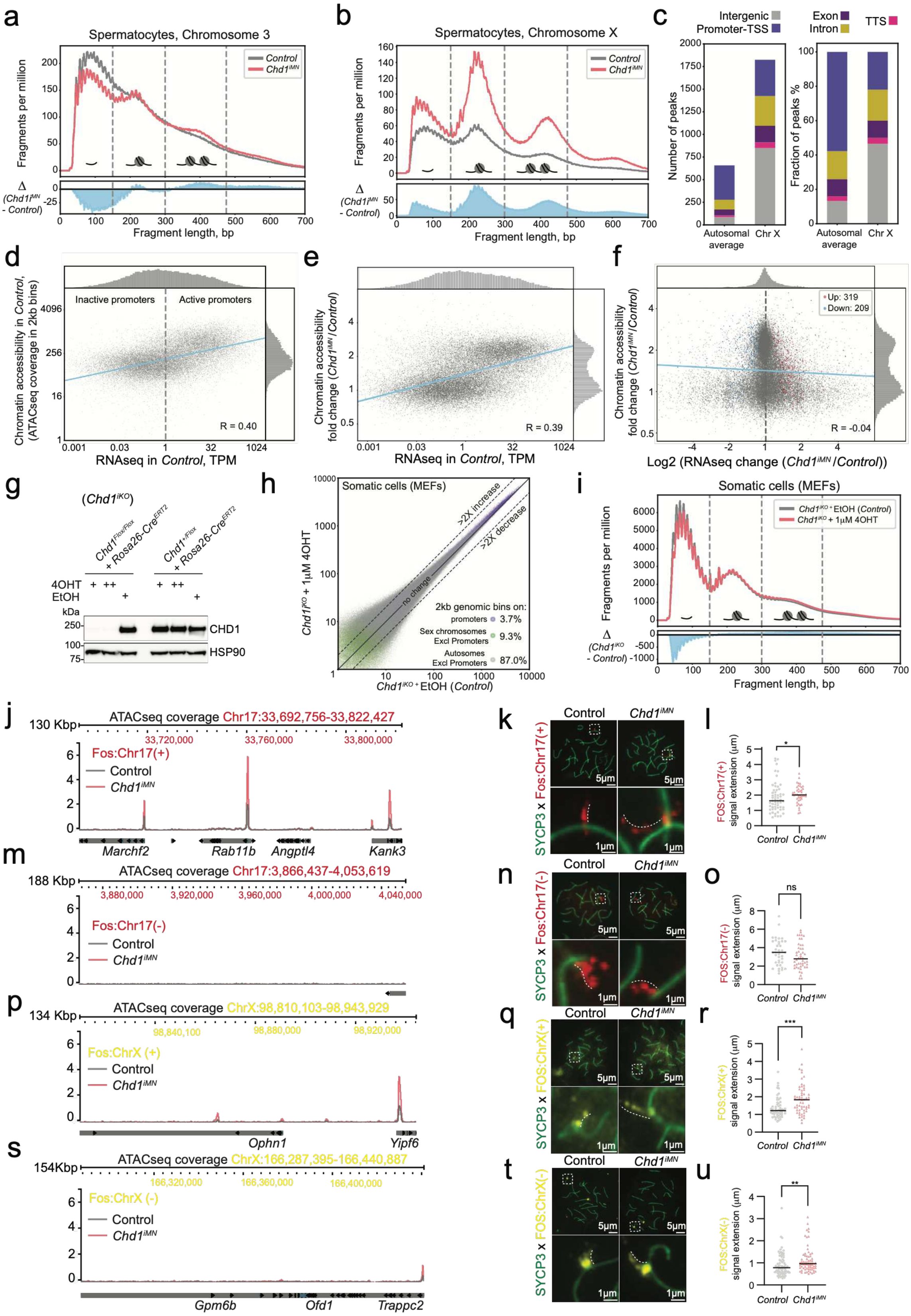

Supplementary Fig. 3. Chd1 deletion alters chromatin accessibility in meiocytes and is associated with changes in chromatin-loop extension from the synaptonemal complex

(a) ATAC-seq fragment-length distribution on chromosome 3 in control and *Chd1*^{iMN} spermatocytes, representing the typical autosomal pattern. Sequencing reads from three biological replicates were aggregated within each genotype for visualization. The y-axis shows fragment counts normalized to one million mapped fragments within each genotype. The lower blue histogram shows the difference in normalized fragment counts between *Chd1*^{iMN} and control. Fragments were classified as subnucleosomal (<150 bp), mononucleosomal (150–300 bp), or dinucleosomal (300–480 bp). **(b)** ATAC-seq fragment-length distribution on chromosome X in control and *Chd1*^{iMN} spermatocytes, plotted as in (a). **(c)** Distribution of differentially changed ATAC-seq peaks between *Chd1*^{iMN} and control spermatocytes across genomic features. Differential ATAC-seq peaks were defined at FDR < 0.05. Left, number of peaks assigned to each genomic feature across biological replicates, shown separately for all autosomes average and for chromosome X. Right, fraction of peaks assigned to each genomic feature, shown separately for all autosomes average and for chromosome X. TSS abbreviation denotes transcription start sites. Three biological replicates per genotype were analyzed. Autosomal and chromosome X values were not normalized for chromosome length and therefore should not be interpreted as directly comparable measures of peak density. **(d)** Relationship between promoter chromatin accessibility and RNA abundance of the corresponding genes in control spermatocytes. RNA abundance in control samples, expressed as transcripts per million (TPM) after removal of zero values, is shown on the x-axis. ATAC-seq coverage at corresponding promoters, calculated in 2 kb bins centered on annotated transcription start sites, is shown on the y-axis. Both axes are plotted on a log2 scale, with linear values indicated for clarity. Marginal histograms show the distributions of transcript abundance and promoter accessibility. The solid blue line indicates the least-squares regression fit, and r=0.40 indicates the Pearson correlation coefficient. **(e)** Relationship between RNA abundance in control spermatocytes and the change in promoter accessibility following Chd1 deletion. RNA abundance in control samples, expressed as transcripts per million (TPM) after removal of zero values, is shown on the x-axis. The y-axis shows the fold change in ATAC-seq coverage at corresponding promoters in *Chd1*^{iMN} relative to control, calculated in 2 kb bins centred on annotated transcription start sites. Both axes are plotted on a log2 scale, with linear values indicated for clarity. Marginal histograms show the distributions of transcript abundance and promoter-accessibility changes. The solid blue line indicates the least-squares regression fit, and r=0.39 indicates the Pearson correlation coefficient. **(f)** Relationship between changes in promoter accessibility and changes in RNA abundance of the corresponding genes. RNA-seq fold change in *Chd1*^{iMN} relative to control is shown on the x-axis. ATAC-seq coverage change at the corresponding promoters, calculated as *Chd1*^{iMN} relative to control, is shown on the y-axis. Promoter accessibility was calculated in 2 kb bins centred on annotated transcription start sites, as in (d). Both axes are plotted on a log2 scale, with linear values shown for clarity. Marginal histograms show the distributions of transcript-abundance changes and promoter-accessibility changes. The solid blue line indicates the least-squares regression fit, and r = −0.04 indicates the Pearson correlation coefficient. Pearson's r was calculated using the log2-transformed values shown. Blue and red points correspond to genes with differentially changed RNA abundance marked in Fig 3g. **(g)** Immunoblot analysis of CHD1 in MEF protein lysates prepared from *Chd1*$^{flox/flox}$ Rosa-CreERT2 cells and *Chd1*$^{+/flox}$ Rosa-CreERT2 cells. Lanes 1–3 show *Chd1*$^{flox/flox}$ Rosa-CreERT2 cells, referred to as *Chd1*^{iKO}. Lanes 4–6 show *Chd1*$^{+/flox}$ Rosa-CreERT2 cells. Cells in lanes 1 and 4 were treated with 1 µM 4-hydroxy tamoxifen (4-OHT) for 24 h before isolation. Cells in lanes 2 and 5 were treated with 5 µM 4-OHT for 24 h before isolation. Cells in lanes 3 and 6 were treated with ethanol (ETOH) as a vehicle-only control for 24 h before isolation. 4-OHT is expected to delete both floxed *Chd1* alleles in lanes 1 and 2, leading to loss of CHD1 expression. HSP90 is shown as a loading control. A total of 50 µg of protein was loaded per lane, and molecular weight marker positions are indicated. **(h)** Scatterplot of ATAC-seq coverage across corresponding 2 kb genomic bins in Chd1iKO MEFs treated with ethanol or 4-OHT. Three biological replicates were merged within each condition for visualization. Each point represents the same genomic location in both conditions. Genomic bins are displayed from low to high ATAC-seq coverage. Bins with unchanged chromatin accessibility lie along the diagonal, indicated by the solid black line. Bins with higher ATAC-seq coverage in 4-OHT-treated cells are located above the diagonal, whereas bins with higher coverage in ethanol-treated cells are located below the diagonal. Dashed black lines indicate twofold increases or decreases in accessibility in 4-OHT-treated cells relative to ethanol-treated cells. Genomic bins overlapping annotated promoters are shown in purple. Bins from chromosomes X and Y are shown in green, excluding promoter-overlapping bins. Remaining autosomal bins are shown in grey. **(i)** ATAC-seq fragment-length distributions in ethanol- and 4-OHT-treated Chd1iKO MEFs, plotted as in (a). Sequencing reads from three biological replicates were aggregated within each condition for visualization. **(j)** ATAC-seq signal in control and *Chd1*^{iMN} spermatocytes across a chromosome 17 region spanned by fosmid probes. This region shows significantly increased ATAC-seq coverage in *Chd1*^{iMN} and is denoted by "+". **(k)** Representative images of fosmid DNA-FISH staining for the chromosome 17 region shown in (j). Scale bars are as indicated. **(l)** Quantification of fosmid-signal extension from the SYCP3-labelled chromosome axis for the chromosome 17 region shown in (j). For each nucleus, one extension measurement was obtained from the chromosome axis to the distal edge of the fosmid signal, as illustrated in (Fig. 3i), and expressed in µm. Two independent biological analyses were performed and both produced the same direction of effect. At least 45 nuclei were analyzed per genotype in both analyses. Mann–Whitney U tests were used to compare nucleus-level measurements between genotypes within each analysis. Mann–Whitney U test for depicted comparison: P = 0.0260. Control, n = 45 nuclei; *Chd1*^{iMN}, n = 48 nuclei. **(m)** ATAC-seq signal in control and *Chd1*^{iMN} spermatocytes across a chromosome 17 region spanned by fosmid probes. This region shows no significant change in ATAC-seq coverage in *Chd1*^{iMN} and is denoted by "-". **(n)** Representative images of fosmid DNA-FISH staining for the chromosome 17 region shown in (m). Scale bars are as indicated. **(o)** Quantification of fosmid-signal extension from the SYCP3-labelled chromosome axis for the chromosome 17 region shown in (m). For each nucleus, one extension measurement was obtained from the chromosome axis to the distal edge of the fosmid signal, as illustrated in (Fig 3i), and expressed in µm. Two independent biological analyses were performed and both produced the same direction of effect. At least 38 nuclei were analyzed per genotype in both analyses. Mann–Whitney U tests were used to compare nucleus-level measurements between genotypes within each analysis. Mann–Whitney U test for depicted comparison: P = 0.2187. Control, n = 38 nuclei; *Chd1*^{iMN}, n = 48 nuclei. **(p)** ATAC-seq signal in control and *Chd1*^{iMN} spermatocytes across a chromosome X region spanned by fosmid probes. This region shows significantly increased ATAC-seq coverage in *Chd1*^{iMN} and is denoted by "+". **(q)** Representative images of fosmid DNA-FISH staining for the chromosome X region shown in (p). Scale bars are as indicated. **(r)** Quantification of fosmid-signal extension from the SYCP3-labelled chromosome axis for the chromosome X region shown in (p). For each nucleus, one extension measurement was obtained from the chromosome axis to the distal edge of the fosmid signal, as illustrated in (Fig. 3i), and expressed in µm. Two independent biological analyses were performed and both produced the same direction of effect. At least 57 nuclei were analyzed per genotype in both analyses. Mann–Whitney U tests were used to compare nucleus-level measurements between genotypes within each analysis. Mann–Whitney U test for depicted comparison: P = 0.0001. Control, n = 59 nuclei; *Chd1*^{iMN}, n = 57 nuclei. **(s)** ATAC-seq signal in control and *Chd1*^{iMN} spermatocytes across a chromosome X region spanned by fosmid probes. This region shows no significant change in ATAC-seq coverage in *Chd1*^{iMN} and is denoted by "-". **(t)** Representative images of fosmid DNA-FISH staining for the chromosome X region shown in (s). Scale bars are as indicated. **(u)** Quantification of fosmid-signal extension from the SYCP3-labelled chromosome axis for the chromosome X region shown in (s). For each nucleus, one extension measurement was obtained from the chromosome axis to

the distal edge of the fosmid signal, as illustrated in (Fig. 3i), and expressed in µm. Two independent biological analyses were performed and both produced the same direction of effect. At least 71 nuclei were analyzed per genotype in both analyses. Mann–Whitney U tests were used to compare nucleus-level measurements between genotypes within each analysis. Mann–Whitney U test for depicted comparison: P = 0.0017. Control, n = 83 nuclei; *Chd1*^{iMN}, n = 71 nuclei.

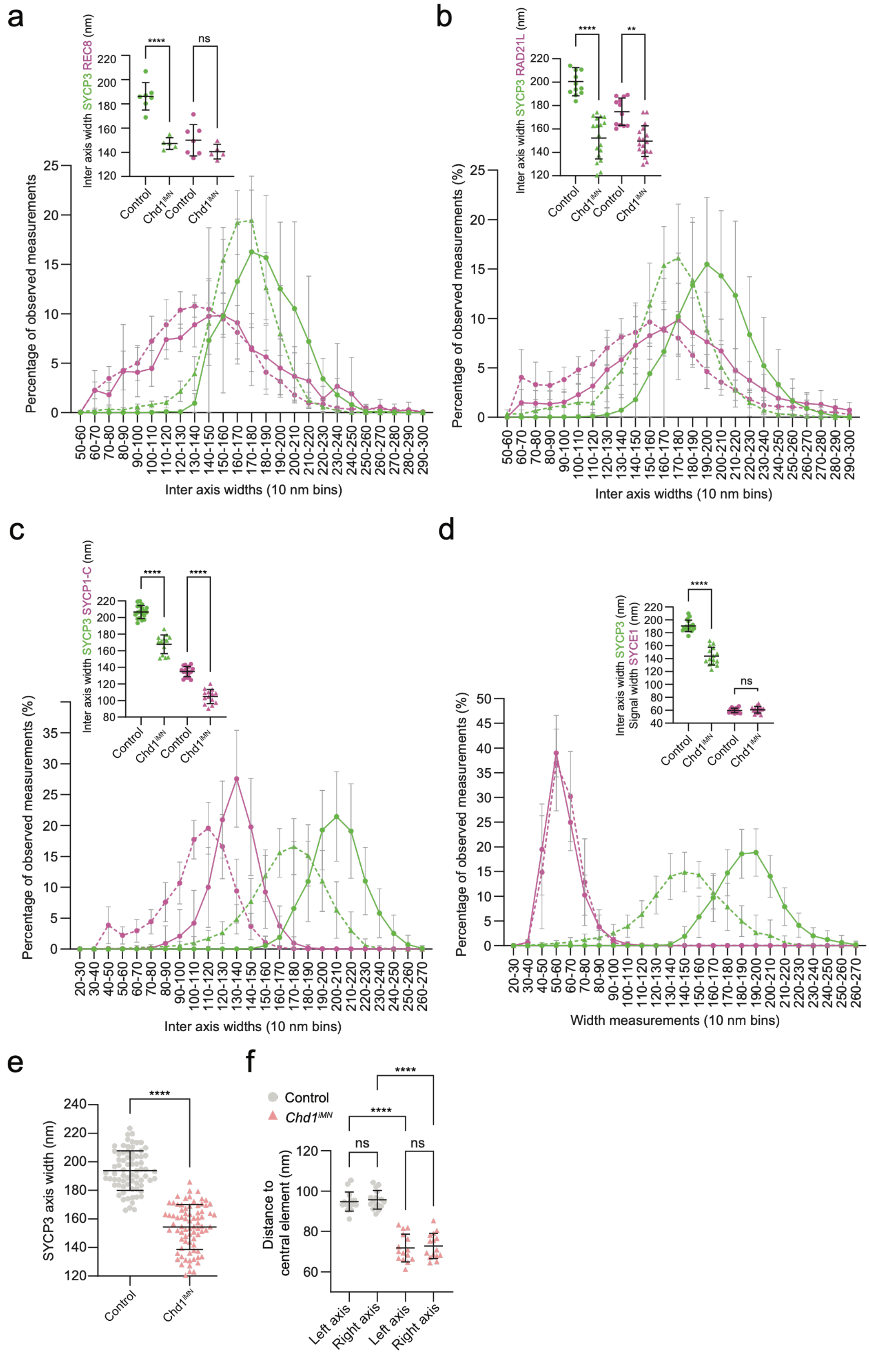

2169

2170 **Supplementary Fig.4. Synaptonemal complex morphology is altered following**

2171 **conditional Chd1 deletion**

(a) Upper panel, quantification of distances between paired SYCP3 or REC8 signal maxima in pachytene-stage nuclei. Measurements were derived from aligned, nucleus-averaged line-trace profiles generated from at least five nuclei from one biological replicate per genotype, as described in Fig. 4g. SYCP3: two-way ANOVA, P < 0.0001; control, n = 7 nuclei; *Chd1^{iMN}*, n = 5 nuclei. REC8: two-way ANOVA, P = 0.0993; control, n = 7 nuclei; *Chd1^{iMN}*, n = 5 nuclei. Lower panel, frequency distributions of REC8 and SYCP3 inter-signal distances using 10-nm bins. Measurements represent nucleus-level technical observations from one biological replicate per genotype. **(b)** Upper panel, quantification of distances between paired SYCP3 or RAD21L signal maxima in early pachytene-stage nuclei, analyzed as in (a). SYCP3: two-way ANOVA, P < 0.0001; control, n = 12 nuclei; *Chd1^{iMN}*, n = 18 nuclei. RAD21L: two-way ANOVA, P = 0.0016; control, n = 12 nuclei; *Chd1^{iMN}*, n = 18 nuclei. Lower panel, frequency distributions of RAD21L and SYCP3 inter-signal distances using 10-nm bins. Measurements represent nucleus-level technical observations from one biological replicate per genotype. **(c)** Upper panel, quantification of distances between paired SYCP3 signals and paired SYCP1 C-terminal signals in pachytene-stage nuclei, analyzed as in (a). SYCP3: two-way ANOVA, P < 0.0001; control, n = 31 nuclei; *Chd1^{iMN}*, n = 31 nuclei. SYCP1-C: two-way ANOVA, P < 0.0001; control, n = 31 nuclei; *Chd1^{iMN}*, n = 31 nuclei. Lower panel, frequency distributions of SYCP1-C and SYCP3 inter-signal distances using 10-nm bins. Measurements represent nucleus-level technical observations from one biological replicate per genotype. **(d)** Upper panel, quantification of distances between paired SYCP3 signal maxima and of SYCE1 signal width in pachytene-stage nuclei. SYCP3: two-way ANOVA, P < 0.0001; control, n = 15 nuclei; *Chd1^{iMN}*, n = 16 nuclei. SYCE1: two-way ANOVA, P = 0.9788; control, n = 15 nuclei; *Chd1^{iMN}*, n = 16 nuclei. Lower panel, frequency distributions of SYCP3 inter-signal distances and SYCE1 signal widths using 10-nm bins. Measurements represent nucleus-level technical observations from one biological replicate per genotype. **(e)** Summary of the mean distance between paired SYCP3 signal maxima in pachytene-stage nuclei analyzed in (a-d). The combined dataset comprises 72 control and 77 *Chd1^{iMN}* nuclei from four independent biological replicate pairs, with each pair corresponding to a different co-staining experiment. Mann–Whitney U test comparing nucleus-level technical observations, P < 0.0001. This analysis does not account for clustering of nuclei within biological replicate. **(f)** Quantification of the distance from the SYCE1 central-element signal maximum to the left or right SYCP3 axial-element signal maximum in control and *Chd1^{iMN}* pachytene nuclei. Measurements were derived from aligned, nucleus-averaged line-trace profiles generated from 15 control and 16 *Chd1^{iMN}* nuclei from one biological replicate per genotype. Left- and right-side distances were measured within the same nuclei. Two-way repeated-measures ANOVA, with genotype as the between-nucleus factor and side as the within-nucleus factor, followed by multiple-comparisons testing: control left versus control right, P = 0.9176; *Chd1^{iMN}* left versus *Chd1^{iMN}* right, P = 0.8996; control left versus *Chd1^{iMN}* left, P < 0.0001; and control right versus *Chd1^{iMN}* right, P < 0.0001. Measurements represent nucleus-level technical observations from one biological replicate per genotype.

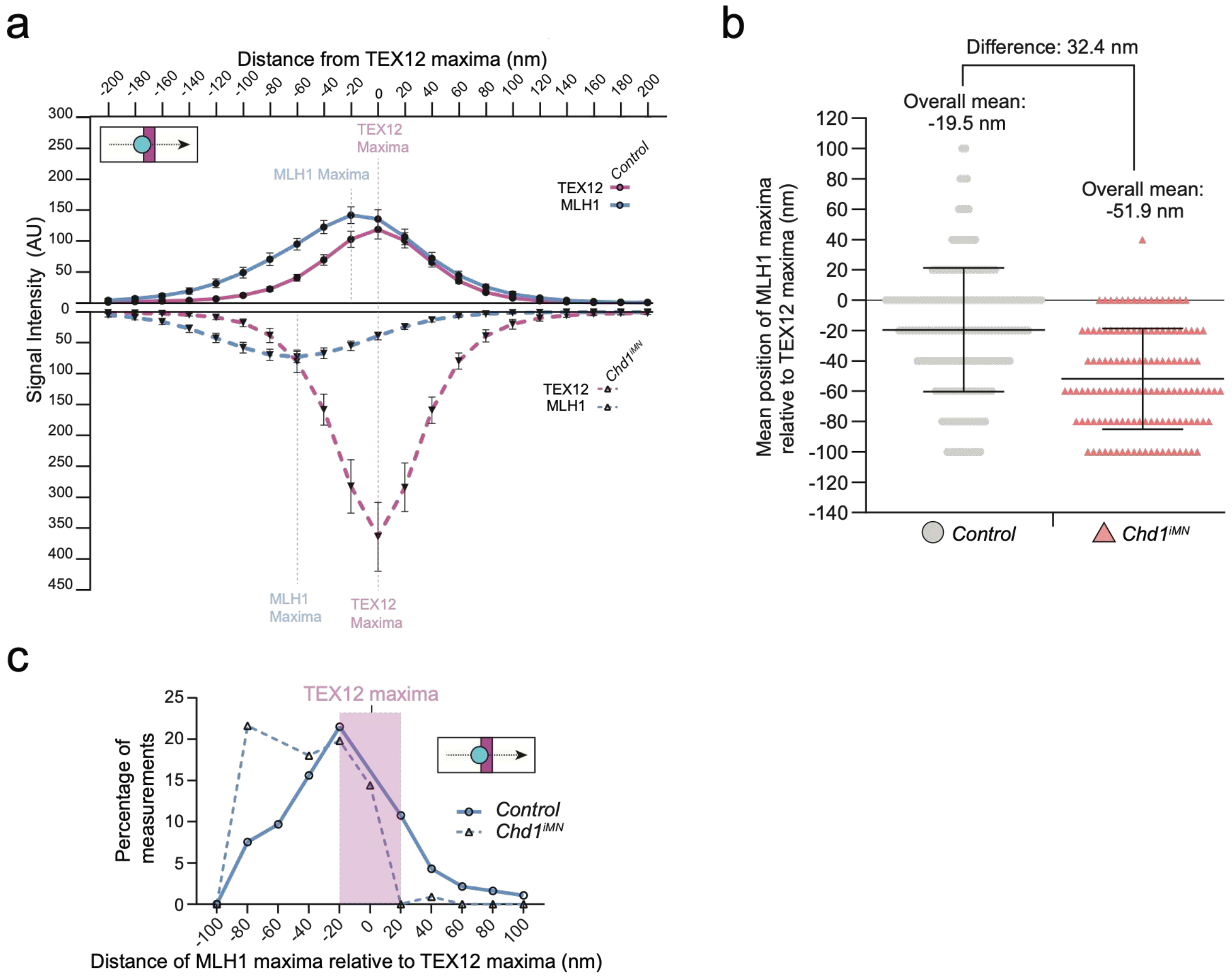

Supplementary Fig.5 . MLH1 positioning relative to TEX12 is altered in *Chd1*^{iMN} mutants

(a) Mean TEX12 and MLH1 signal-intensity profiles measured from STED images of pachytene-stage nuclear surface spreads prepared from adult control and *Chd1*^{iMN} testes, as shown in Fig. 5j. Line traces were drawn through individual MLH1 foci and the corresponding TEX12 signal. Traces were aligned such that the TEX12 intensity maximum was positioned at 0 nm. TEX12 is shown in magenta and MLH1 in blue. **(b)** Positions of individual MLH1 signal maxima relative to the corresponding TEX12 signal maximum for the line traces analyzed in (a). One position measurement was obtained per MLH1 focus. Control, n = 197 traces; *Chd1*^{iMN}, n = 131 traces, obtained from at least nine nuclei from one biological replicate per genotype. Mann–Whitney U test, $P < 0.001$. Measurements represent focus-level technical observations from one biological replicate per genotype. **(c)** Relative-frequency distribution of distances between MLH1 and TEX12 signal maxima measured in (b). The pink box is provided as a visual guide indicating the position of the TEX12 signal.

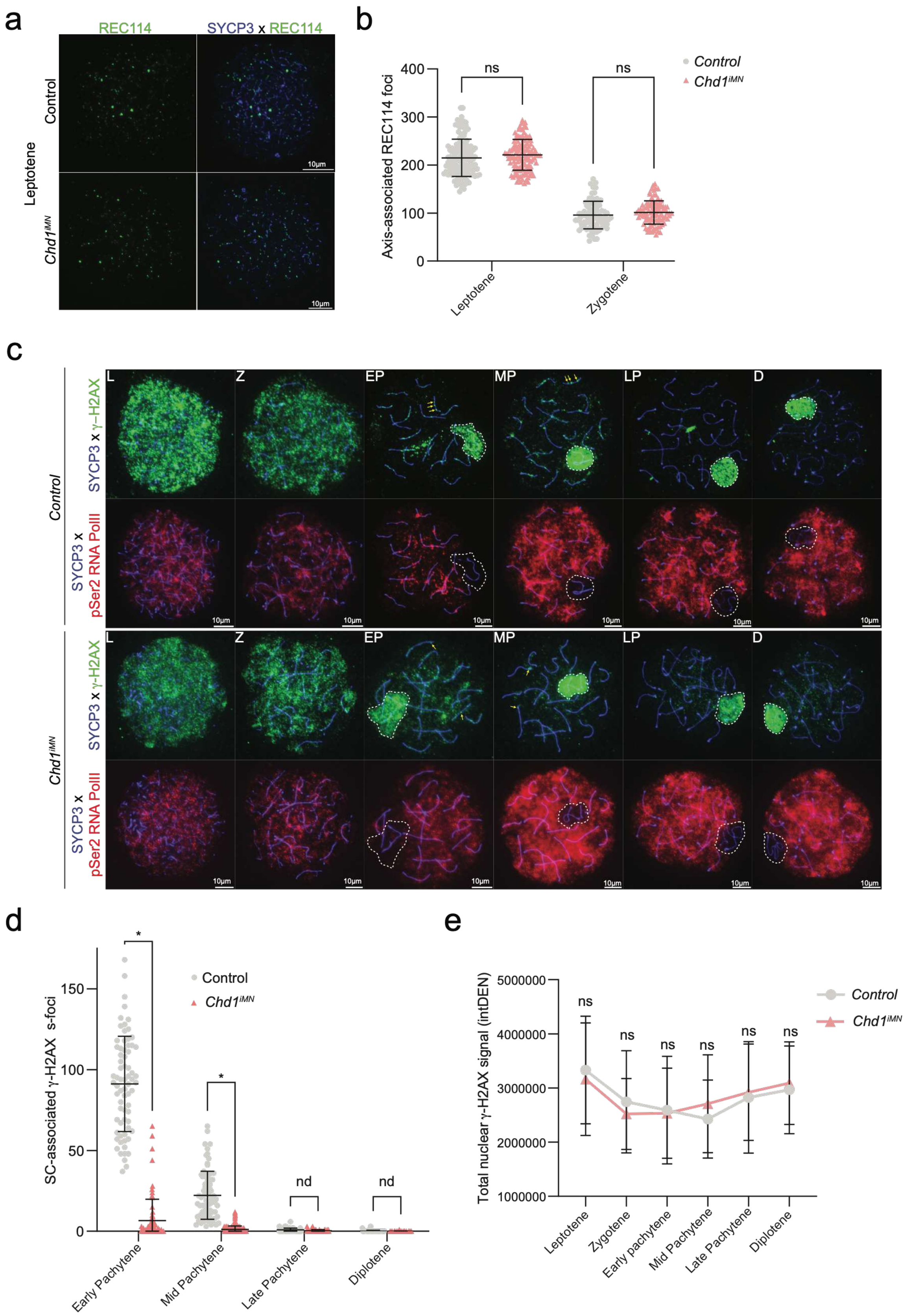

Supplementary Fig.6 . Double-strand break formation and global γ-H2AX accumulation is broadly preserved following conditional *Chd1* deletion

(a) Representative nuclear surface spreads prepared from adult control and *Chd1*^{iMN} testes stained for SYCP3 and REC114. SYCP3 is shown in blue and REC114 in green. Scale bars are as indicated. **(b)** Quantification of SC-associated REC114 foci in leptotene and zygotene, as in (a). Measurements are from at least three biological replicates. For each biological replicate, at least 20 nuclei were quantified per substage. Mann-Whitney U test: $P = 0.101$ and $P = 0.0599$ for the indicated comparisons from left to right; both comparisons were not significant. **(c)** Representative nuclear surface spreads prepared from adult control and *Chd1*^{iMN} testes stained for SYCP3, γ-H2AX and phospho-Ser2 RNA polymerase II. SYCP3 is shown in blue, γ-H2AX in green and phospho-Ser2 RNA polymerase II in red. L= Leptotene, Z= Zygotene, EP= Early Pachytene, MP= Mid Pachytene, LP = Late Pachytene, DIP=Diplotene. Scale bars are as indicated. **(d)** Quantification of SC-associated γ-H2AX foci across selected prophase I stages, as in (c). Measurements are from at least three biological replicates. For each biological replicate, at least 15 nuclei were quantified per substage. Mann–Whitney U test: $P < 0.001$, $P < 0.001$, $P = 0.006$ and $P > 0.999$ for the indicated comparisons from left to right. **(e)** Quantification of total nuclear γ-H2AX signal, measured as integrated density, across selected prophase I stages, as in (c). Measurements are from at least three biological replicates. For each biological replicate, at least 30 nuclei were quantified per substage. Mann–Whitney U test: $P = 0.9443$, $P = 0.7155$, $P = 0.9989$, $P = 0.0821$, $P = 0.9891$ and $P = 0.9820$ for the indicated comparisons from left to right; all comparisons were not significant.

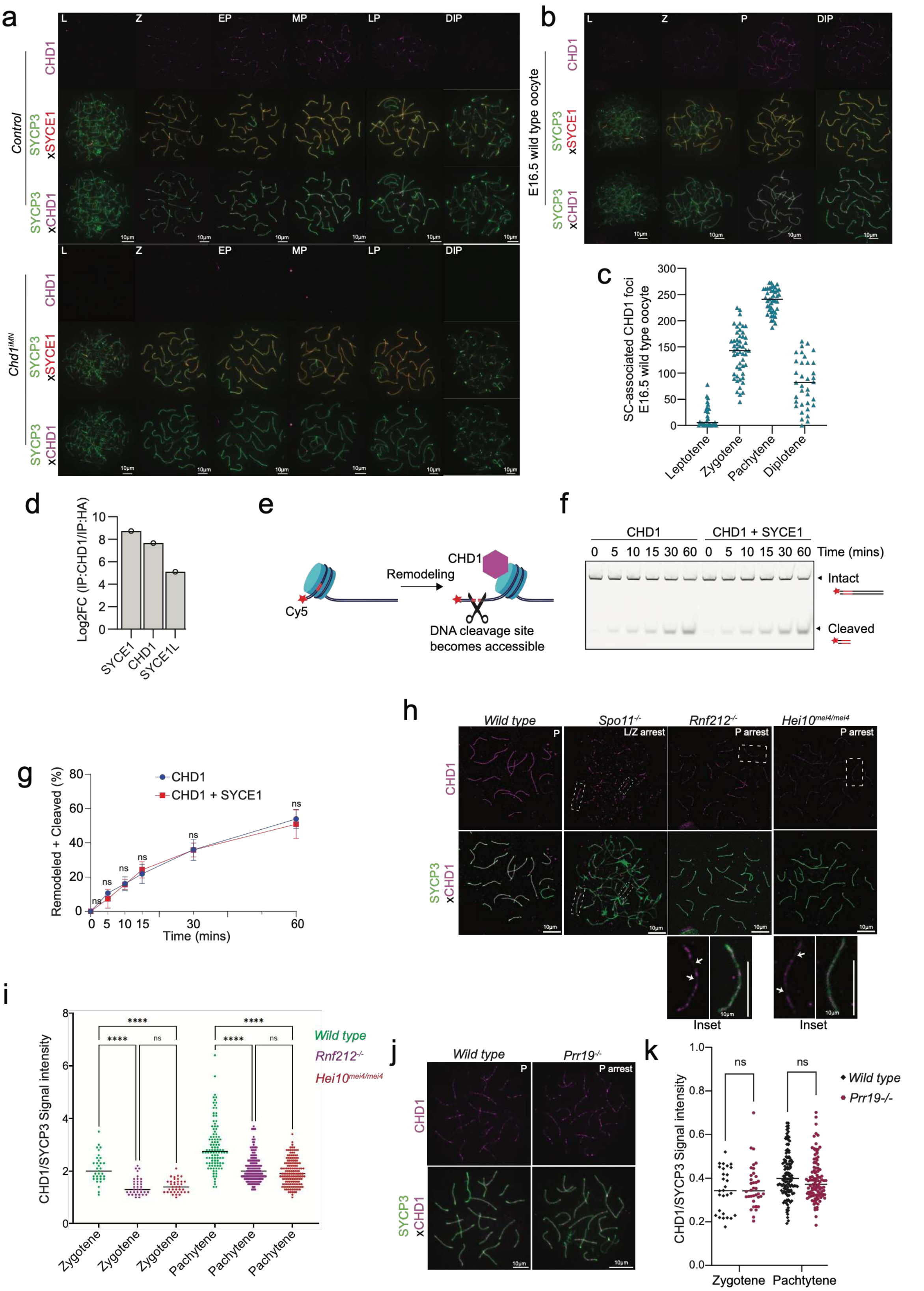

Supplementary Fig.7 . CHD1 localization to the SC is not sex-specific, is independent of SPO11-mediated DSBs and is modulated by RNF212 and HEI10

(a) Representative nuclear surface spreads prepared from adult control and *Chd1*^{iMN} testes across the indicated prophase I substages and stained for SYCP3, CHD1, and SYCE1. SYCP3 is shown in green, CHD1 in magenta, and SYCE1 in red. Images are representative of equivalent stainings performed in at least three independent biological replicates per genotype. L= Leptotene, Z= Zygotene, EP= Early Pachytene, MP= Mid Pachytene, LP = Late Pachytene, DIP=Diplotene. Scale bars are as indicated. **(b)** Nuclear surface spreads prepared from wild-type embryonic ovaries at embryonic day 16.5 and stained for CHD1, SYCP3, and SYCE1 across the indicated prophase I substages. CHD1 is shown in magenta, SYCP3 in green, and SYCE1 in red. Images are representative of equivalent stainings performed in three independent biological replicates. Labels as in (a). Scale bars are as indicated. **(c)** Quantification of SC-associated CHD1 foci across the indicated prophase I substages in embryonic ovarian spreads, as in (b). Measurements were obtained from two independent biological replicates, with at least 10 nuclei analyzed per substage and biological replicate. Data are shown descriptively; no statistical comparison was performed. **(d)** Exploratory mass spectrometry analysis of CHD1 and HA control immunoprecipitates generated from spermatocyte-enriched STA-PUT fractions prepared from wild-type testes. One CHD1 immunoprecipitation and one HA control immunoprecipitation were analyzed, each generated from 0.5 mg total protein lysate. Data show the log2 fold enrichment of CHD1 and selected central element proteins in the CHD1 immunoprecipitate relative to the HA control immunoprecipitate. No inferential statistical analysis was performed because only one biological replicate was analyzed per condition. **(e)** Schematic of the nucleosome-remodelling assay used to measure DNA accessibility to DpnII. End-positioned mononucleosomes assembled on Cy5-labelled DNA were incubated with recombinant full-length human CHD1 and DpnII in the presence or absence of excess recombinant full-length human SYCE1. Reactions were stopped at the indicated time points, deproteinized, and analyzed by native PAGE. **(f)** Representative native PAGE analysis of the nucleosome-remodelling assay described in (e). Bands corresponding to uncleaved nucleosomes and cleaved/remodelled products are indicated. The experiment was repeated independently at least three times. **(g)** Quantification of the cleaved/remodelled product as a percentage of total lane signal from assays performed as in (f). Data are shown as mean ± SD from at least three independent remodelling experiments. **(h)** Representative nuclear surface spreads prepared from adult wild-type, *Spo11*$^{-/-}$, *Rnf212*$^{-/-}$ and *Hei10*$^{mei4/mei4}$ testes and stained for SYCP3 and CHD1. SYCP3 is shown in green and CHD1 in magenta. The wild-type image is representative of mid-pachytene (P), the *Spo11*$^{-/-}$ image of leptotene/zygotene arrest (L/Z Arrest), and the *Rnf212*$^{-/-}$ and *Hei10*$^{mei4/mei4}$ images of pachytene arrest (P Arrest). Images are representative of equivalent stainings performed in three biological replicates. Scale bars are as indicated. **(i)** Quantification of total nuclear CHD1 signal relative to total nuclear SYCP3 signal across genotypes and prophase I substages, as shown in (h). Measurements are from at least three biological replicates. For each biological replicate, at least 30 nuclei were quantified per substage. Mann-Whitney U test for the indicated comparisons: ****, $P < 0.0001$; n.s., non-significant. **(j)** Representative nuclear surface spreads prepared from adult wild-type and *Prr19*$^{-/-}$ testes and stained for SYCP3 and CHD1. SYCP3 is shown in green and CHD1 in magenta. The wild-type image is representative of early pachytene, and the *Prr19*$^{-/-}$ image of pachytene arrest. Images are representative of equivalent stainings performed in three biological replicates. Labels as in (h). Scale bars are as indicated. **(k)** Quantification of total nuclear CHD1 signal relative to total nuclear SYCP3 signal in wild-type and *Prr19*$^{-/-}$ samples, as in (i). Measurements are from at least three biological replicates. For each biological replicate, at least 30 nuclei were quantified per substage. Mann-Whitney U test: $P = 0.84$ and $P = 0.40$ for the indicated comparisons from left to right; both comparisons were not significant.

Supplementary Files

This is a list of supplementary files associated with this preprint. Click to download.

- SupplementaryTable1.xlsx
- SupplementaryTable2.xlsx
- SupplementaryTable3.xlsx
- SupplementaryTable4.xlsx
- SupplementaryTable5.xlsx
- SupplementaryTable6.xlsx
